# Tetracationic Bis‐Triarylborane 1,3‐Butadiyne as a Combined Fluorimetric and Raman Probe for Simultaneous and Selective Sensing of Various DNA, RNA, and Proteins

**DOI:** 10.1002/chem.201905328

**Published:** 2020-04-24

**Authors:** Hashem Amini, Željka Ban, Matthias Ferger, Sabine Lorenzen, Florian Rauch, Alexandra Friedrich, Ivo Crnolatac, Adriana Kenđel, Snežana Miljanić, Ivo Piantanida, Todd B. Marder

**Affiliations:** ^1^ Institut für Anorganische Chemie and Institute for Sustainable Chemistry & Catalysis with Boron Julius-Maximilians-Universität Würzburg Würzburg 97074 Germany; ^2^ Laboratory for Study of Interactions of Biomacromolecules Division of Organic Chemistry & Biochemistry Ruđer Bošković Institute Zagreb HR-10000 Croatia; ^3^ Division of Analytical Chemistry Department of Chemistry Faculty of Science University of Zagreb Zagreb HR-10000 Croatia

**Keywords:** borane, circular dichroism, DNA/RNA sensor, fluorescent probe, protein sensor, Raman probe

## Abstract

A bis‐triarylborane tetracation (4‐Ar_2_B‐3,5‐Me_2_C_6_H_2_)‐C≡C−C≡C‐(3,5‐Me_2_C_6_H_2_‐4‐BAr_2_ [Ar=(2,6‐Me_2_‐4‐NMe_3_‐C_6_H_2_)^+^] (**2^4+^**) shows distinctly different behaviour in its fluorimetric response than that of our recently published bis‐triarylborane 5‐(4‐Ar_2_B‐3,5‐Me_2_C_6_H_2_)‐2,2′‐(C_4_H_2_S)_2_–5′‐(3,5‐Me_2_C_6_H_2_‐4‐BAr_2_) (**3^4+^**). Single‐crystal X‐ray diffraction data on the neutral bis‐triarylborane precursor **2** **N** confirm its rod‐like dumbbell structure, which is shown to be important for DNA/RNA targeting and also for BSA protein binding. Fluorimetric titrations with DNA/RNA/BSA revealed the very strong affinity of **2^4+^** and indicated the importance of the properties of the linker connecting the two triarylboranes. Using the butadiyne rather than a bithiophene linker resulted in an opposite emission effect (quenching vs. enhancement), and **2^4+^** bound to BSA 100 times stronger than **3^4+^**. Moreover, **2^4+^** interacted strongly with ss‐RNA, and circular dichroism (CD) results suggest ss‐RNA chain‐wrapping around the rod‐like bis‐triarylborane dumbbell structure like a thread around a spindle, a very unusual mode of binding of ss‐RNA with small molecules. Furthermore, **2^4+^** yielded strong Raman/SERS signals, allowing DNA or protein detection at ca. 10 nm concentrations. The above observations, combined with low cytotoxicity, efficient human cell uptake and organelle‐selective accumulation make such compounds intriguing novel lead structures for bio‐oriented, dual fluorescence/Raman‐based applications.

## Graphical abstract

**Got the link?** Linking two dicationic triarylborane units by Raman‐responsive 1,3‐butadiyne yielded a triple‐action probe: fluorimetric and SERS sensing of ds‐DNA, RNA and proteins, as well as circular dichroism sensing of ss‐RNA (see figure).

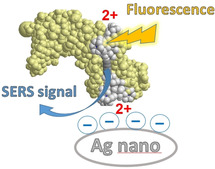

## Introduction

The use of fluorescent labels to image specific small molecules in live cells is an essential tool in biology, medicinal chemistry, and many other related fields of research. However, common fluorescent dyes also have many drawbacks. Due to the considerable size and aromatic nature, and also often being H‐bond donors/acceptors, they may alter the biological activity, cellular localization, and dynamics of the targeted bio‐molecules.^1^ Moreover, even the most advanced fluorescence microscopy techniques are limited to a maximum simultaneous resolution of six different colors.^2^ This is additionally complicated^3^ by unavoidable cross‐talk in organic dyes, energy transfer between quantum dots^4^ and the limited number of suitable features for straightforward decoding in rare‐earth nanocrystals and metal nanoparticles.^5^

Therefore, further development in the increasingly complex bioimaging field essentially requires new fluorophore structures for better bio‐target diversification. Even more useful would be new optical probes based on intrinsically different methods at least approaching similar sensitivities of common fluorophores. Such an aim recently led to the development of vibrational spectroscopy methods, particularly Raman bioimaging microscopy, due to its compatibility with aqueous biological samples.2d

Phenyl end‐capped polyynes have been employed as alkyne tags for Raman visualization of mobile, small molecules in cells^6^ as well as for surface‐enhanced Raman spectroscopy (SERS) multiplex cellular imaging,^7^ showing very promising applications in supermultiplexed optical imaging and barcoding.2d However, these polyyne‐probes relied exclusively on their Raman response, and thus were not combined with other sensing techniques.

Although fluorescence, under certain experimental conditions, interferes with Raman measurements, by causing significant background signal,^8^ for some chromophores it is possible to obtain a satisfying compromise. In recent years, the combination of Raman and fluorescence spectroscopy has emerged as a way to circumvent some of the intrinsic problems of Raman spectroscopy (i.e. low signal strength, long acquisition times) or to gain additional information on a given system.^9^ Thus, in the first multimodal approaches, fluorescence was used as a fast macroscopic scanning method, prior to a detailed Raman analysis, or to confirm conclusions made via Raman‐based imaging.^10^ Raman spectroscopy, in combination with the use of fluorescently labelled molecules or quantum dots, also proved to be an interesting approach in some cell imaging applications.^11^ In disease diagnostics, dual Raman and fluorescence spectroscopy was used by several groups in a complementary fashion to improve the accuracy and sensitivity of the diagnosis.^12^ Dual fluorescence and Raman spectroscopy was successfully employed to investigate and visualize intracellular drug delivery.^13^ The design of small molecules with inherently strong Raman and fluorescence responses is a rather novel approach,^14^ which has already produced much information on the location, environment14b or concentration14a of the small molecule inside a cell.

**Scheme 1:**
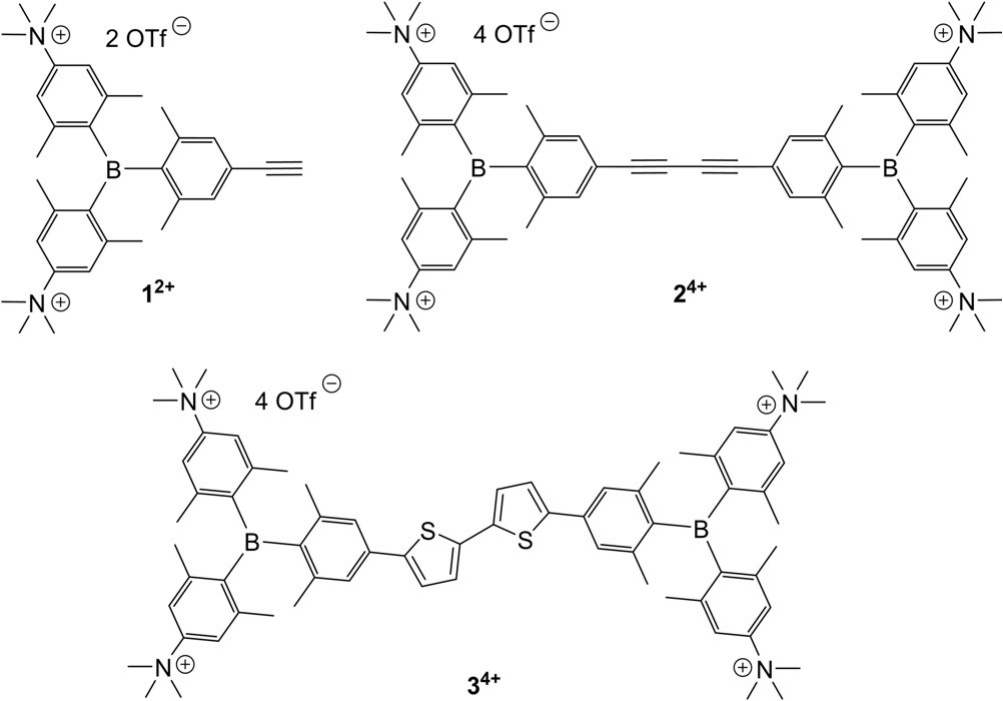
The ethynyl‐triarylborane monomer (**1^2+^**), bis‐triarylborane 1,3‐butadiyne (**2^4+^**) and previously studied bithiophene‐linked bis‐triarylborane tetracation (**3^4+^**).

Recently, triarylboranes have emerged as a structurally novel class of compounds suitable for biological imaging applications.^15^ Over the last decades, triarylboranes have also found applications in many other fields, such as anion sensors, OLEDs and non‐linear optical materials.^16^ Due to its vacant p_z_‐orbital, the three‐coordinate boron in a triarylborane is a strong π‐acceptor. However, it is also Lewis‐acidic and sensitive to hydrolysis. Bulky substituents can stabilize three‐coordinate boron against decomposition by air and moisture, while maintaining its π‐acceptor strength.^17^ Using Gabbaï's approach^18^ we developed the water‐soluble, water‐stable and non‐cytotoxic tetracationic chromophore **3^4+^** (Scheme 1) which was successfully utilized in live cell imaging.^19^ Further studies revealed **3^4+^** to be a structurally novel DNA/RNA/protein probe of high affinity and selective fluorimetric and chirooptic response.^20^

The question arose to which extent the chromophoric linker (bithiophene) is responsible for the observed spectroscopic responses and affinity. In a search for linkers which could also be non‐conventional response probes, 1,3‐butadiyne attracted our attention for several reasons. It is a rigid, rod‐like symmetrical linker, excellent for precise orientation of terminal triarylborane cations (Scheme 1, **2^4+^**), and it is completely inert, in terms of covalent and non‐covalent interactions, to DNA/RNA/protein. Even more importantly, it exhibits an intense Raman band with a narrow linewidth in the Raman‐silent spectral region (2250–2000 cm^−1^) convenient for Raman‐based probing in aqueous solutions. Additionally, the intensity of response can be strongly increased by surface‐enhanced Raman scattering (SERS) spectroscopy and related techniques.

**Scheme 2:**
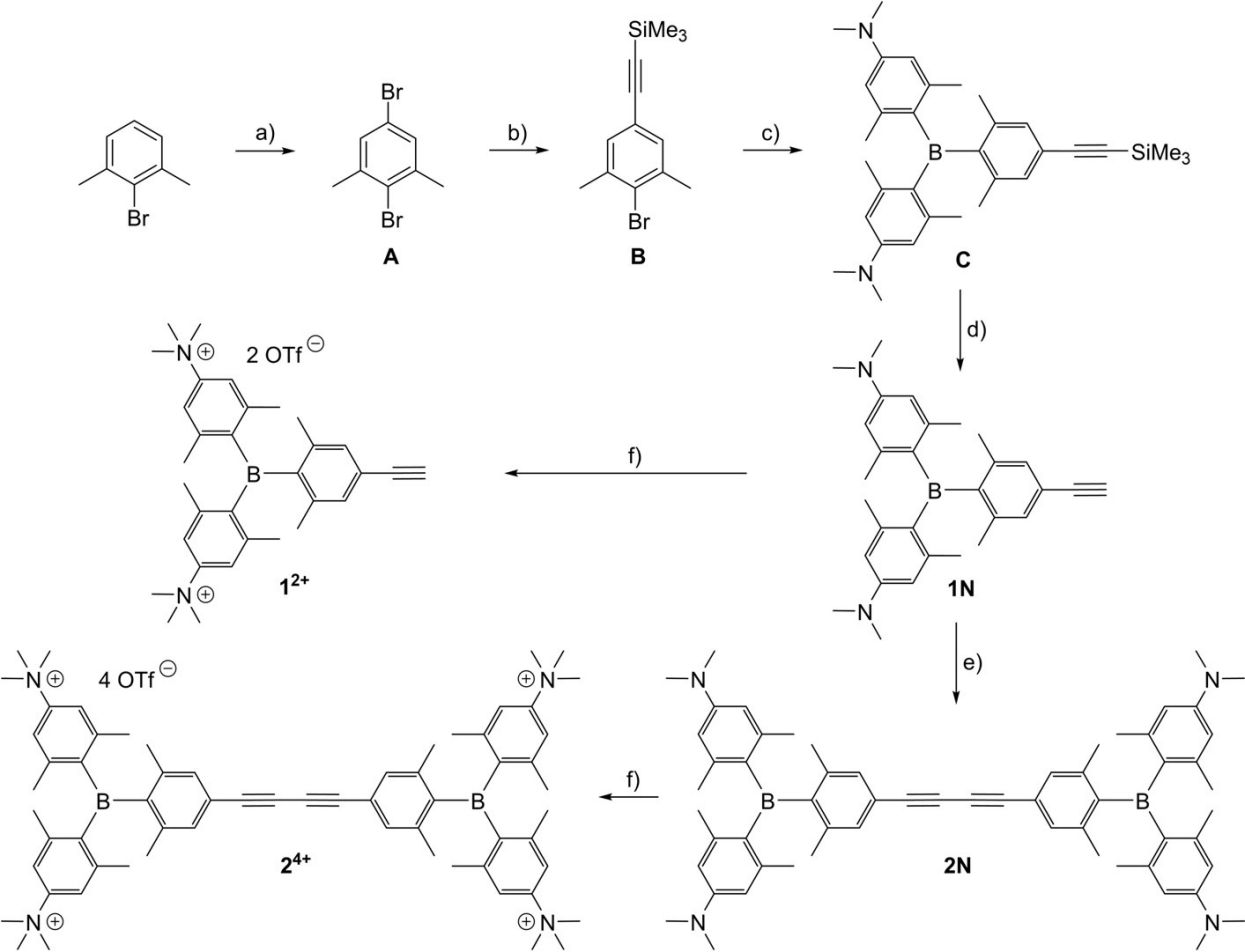
Synthesis of the compounds **1** **N**, **2** **N**, **1^2+^** and **2^4+^**. a) 1. B_2_pin_2_, [Ir(COD)(OMe)]_2_, dtbpy, hexane, 80 °C; 2. CuBr_2_, MeOH/H_2_O, 90 °C, yield: 91 % over two steps; b) trimethylsilylacetylene, Pd(PPh_3_)_2_Cl_2_, CuI, NEt_3_, 80 °C, yield: 62 %; c) *t*BuLi, bis[4‐(*N*,*N*‐dimethylamino)‐2,6‐dimethylphenyl]fluoroborane, hexane, −78 °C to r.t., yield: 47 %; d) KOH, MeOH/THF, r.t., yield: 97 %; e) Pd(PPh_3_)_2_Cl_2_, CuI, I_2_, NEt_3_/THF, r.t., yield: 76 %; f) MeOTf, CH_2_Cl_2_, r.t., yield: 64 % (**1^2+^**), 23 % (**2^4+^**).

To address the above mentioned issues, we designed the new molecule **2^4+^** by replacing the phenyl end‐capping of the shortest polyyne2d (1,3‐butadiyne) with two bis‐triarylborane cations, acting as a fluorescence probe (Scheme 1). For comparison, the dicationic monomer **1^2+^** was also prepared (Scheme 1) as well as their non‐charged precursors **1** **N** and **2** **N** (Scheme 2).

Thus, novel molecule **2^4+^**, and studies of its interactions with DNA/RNA/proteins, will address the potential for the development of innovative combined fluorophore‐Raman probes for the general purpose of non‐covalent probing of DNA/RNA/proteins, and the impact of the nature of the linker connecting two bis‐triarylborane cations on the fluorescent and chirooptic response upon binding to biomacromolecules.

Given the generally weak spontaneous Raman response of molecules in solution, SERS spectroscopy was used to study the binding to biomolecules in aqueous media mimicking the biological environment. The enhancement of the Raman scattering in the vicinity of nanostructured metal surfaces arises from amplification of the electromagnetic field upon excitation of the localized surface plasmon resonances (LSPR) of the molecules when physisorbed on a surface, and from the transfer of electrons from the metal to the molecule and vice versa for chemisorbed molecules.^21^ Even though the charge transfer mechanism is considered to contribute to the overall enhancement to a lesser extent than the electromagnetic one, the total SERS enhancement factor with respect to the normal Raman signal, in most cases, is the product of both mechanisms, reaching up to 10.^10^ Owing to its high sensitivity and ability to produce molecularly specific fingerprint spectra, SERS has been successfully applied for the detection, quantification and biophysical characterization of a variety of biomolecules.^22^

## Results and Discussion

### Synthesis

The key precursor **A**, which was previously only accessible via Sandmeyer type reactions in moderate yields,^23^ was reproducibly prepared in 91 % yield on a 73 mmol scale using a new approach based on literature precedents.^24^ This was achieved by direct, regioselective, Ir‐catalyzed borylation^25^ of the C−H bond of 2‐bromo‐1,3‐dimethylbenzene at the 5‐position. Without any purification, the borylated intermediate was converted into **A**, using CuBr_2_ as a brominating agent. Compound **A** was then cross‐coupled with trimethylsilylacetylene, at the sterically less hindered bromine, giving **B**. For the synthesis of triarylborane **C**, compound **B** was lithiated and bis[4‐(*N*,*N*‐dimethylamino)‐2,6‐dimethylphenyl]fluoroborane^18^ was added. Triarylborane **C** was deprotected using KOH in MeOH/THF to give the neutral terminal alkyne compound **1** **N**. The neutral 1,3‐butadiyne compound **2** **N** was formed by oxidative homo‐coupling of **1** **N** in a Glaser type reaction, using I_2_ as an oxidant. The two neutral compounds **1** **N** and **2** **N** were methylated at the amine groups using methyltriflate in CH_2_Cl_2_. The two cationic species **1^2+^** and **2^4+^** were precipitated by addition of Et_2_O; **1^2+^** was washed with CH_2_Cl_2_/Et_2_O, and **2^4+^** with acetone/Et_2_O.

**Figure 1:**
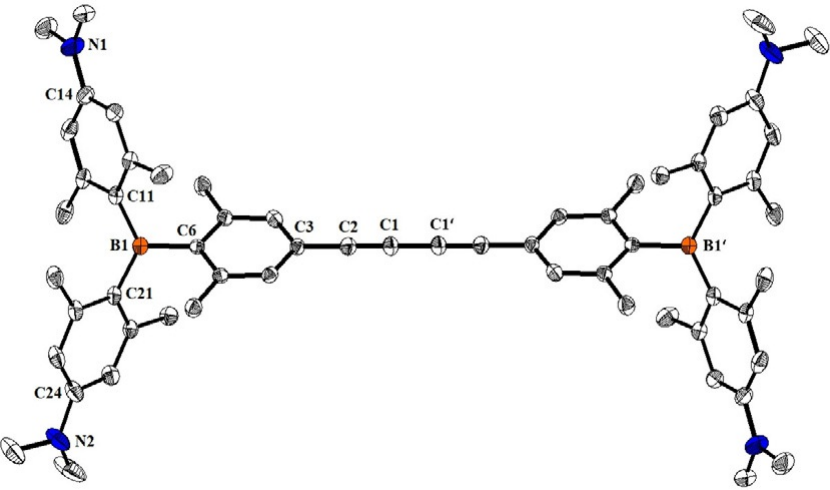
The solid state molecular structure of **2** **N** (50 % probability ellipsoids). Hydrogen atoms and solvent molecules are omitted for clarity. Angles [°] between the plane of the BC_3_‐core (defined by B1, C6, C11 and C21) and the planes of the adjacent aryl rings: 57.9(1) for C6; 49.6(1) for C11; 48.9(1) for C21. Selected distances [Å] and angles [°] for **2** **N**: B1−C6 1.596(2); B1−C11 1.561(2); B1−C21 1.570(2); C1−C1′ 1.373(3); C1−C2 1.202(2); C2−C3 1.431(2); N1−C14 1.381(2); N2−C24 1.390(2); C6‐B1‐C11 116.577(30); C6‐B1‐C21 117.639(28); C11‐B1‐C21 125.764(42); C1‐C2‐C3 177.35(17), C1′‐C1‐C2 179.6(2). Sum of the C‐B‐C angles around B: 359.98(14)°.^37^

Single crystals suitable for X‐ray diffraction analysis were obtained for compounds **1** **N** (Figure S17) and **2** **N**. The solid state molecular structure of **2** **N**, along with selected bond lengths and angles, are depicted in Figure 1, to illustrate the shape and size of the diyne compound. The distance between the two boron atoms B1 and B1′ is 15.457(7) Å. The molecule has an inversion center located between C1 and C1′ and is almost linear, as the relevant C−C−C bond angles of the diyne bridge are all close to 180°.

### Physicochemical properties

Both positively charged compounds (**1^2+^**, **2^4+^**) are moderately soluble in water (*c=*1×10^−3^ m), their solutions being stable for longer periods when stored in the dark. The neutral analogue of **1** **N** was dissolved in DMSO to prepare a stock solution (*c=*5×10^−3^ m) which was further diluted with aqueous buffer prior to every experiment, yielding stable solutions up to the 0.1 m range. The poor solubility of neutral analogue **2** **N** under biorelevant conditions hampered further experiments.

**Figure 2:**
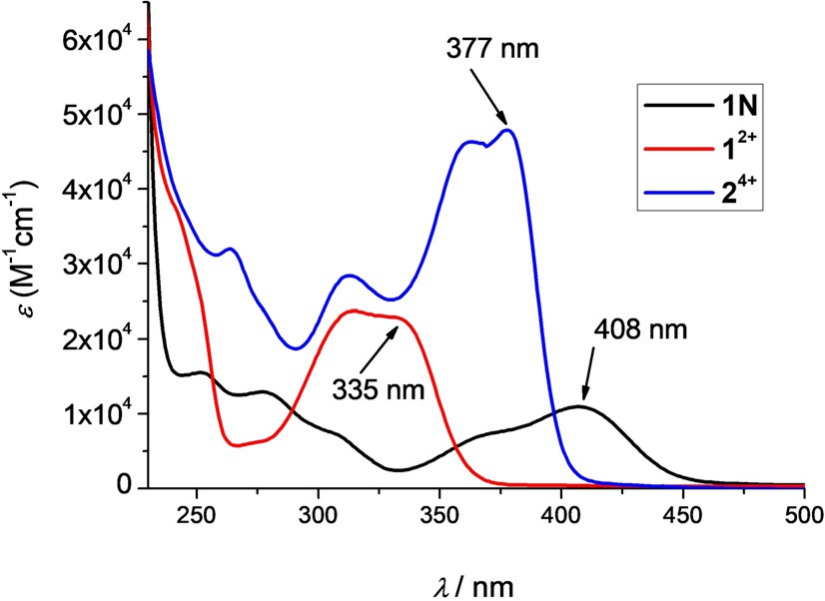
UV/Vis spectra of neutral **1** **N**, **1^2+^**, and **2^4+^** in water.

The absorbances of the studied compounds (Figure 2) were proportional to their concentrations up to *c=*2×10^−5^ m (Figures S21–S23), and changes in their UV/Vis absorption spectra upon temperature increase up to 90 °C were minor (Figure S24). Reproducibility of the UV/Vis spectra upon cooling to 25 °C was excellent. These findings indicate that the compounds do not aggregate by intermolecular stacking under the experimental conditions used. Absorption maxima and corresponding molar extinction coefficients (*ϵ*) are given in Table 1.

**Table 1:** Photophysical data for compounds **1** **N**, **2** **N**, **1^2+^** and **2^4+^**.

	Solvent	*λ*_abs_ [nm]^[a]^	*ϵ* [M^−1^ cm^−1^]	*λ*_em_ [nm]	Stoke's shift [cm^−1^]	*Φ*_f_	*τ* [ns]	*τ*_0_ [ns]	*k_r_* [10^8^ s^−1^]	*k_nr_* [10^8^ s^−1^]
**1N**	hexane	397	–	447^[b]^	2800	0.10^[b]^	1.5	15.0	0.67	6.0
	toluene	405	25 000	496	4500	0.22	4.4	20.0	0.50	1.8
	Et_2_O	400	–	514	5500	0.27	7.6	28.3	0.35	0.96
	H_2_O^[c]^	408	11 000	502	4600	–	–	–	–	–
**2N**	hexane	386	–	464	4400	0.10	2.0	20.0	0.50	4.5
	toluene	386	60 000	525^[b]^	6900	0.16	4.3	26.9	0.37	1.9
	Et_2_O	386	–	546^[b]^	7600	0.15	6.2	41.3	0.24	1.4
**1^2+^**	EtOH	344	26 000	418	5100	0.15	3.9	26.0	0.38	2.2
	H_2_O	335	23 000	428	6700	0.18	6.4	35.5	0.28	1.3
**2^4+^**	EtOH	384	45 000	414^[b]^	1900	0.26	0.94	3.6	2.8	7.8
	H_2_O	377	49 000	431^[b]^	3100	0.25	1.3	5.2	1.9	5.8

[a] Lowest energy absorption band. [b] For compound **1 N** in hexane, *λ*_ex_ = 385 and 375 nm were used for measuring emission spectrum and quantum yield, respectively. For compound **2^4+^**, *λ*_ex_ = 360 nm (in H_2_O) and *λ*_ex_ = 365 nm (in EtOH) were used for emission and quantum yield measurements. For compound **2 N** in toluene and Et_2_O, a 395 nm cut‐off filter was used for the emission measurement. [c] DMSO stock solution (*c* = 5×10^−3^ m) further diluted with aqueous buffer.

Comparison of their UV/Vis absorption spectra reveals distinct differences between the neutral compound **1** **N** and its charged analogue **1^2+^**. The neutral molecule **1** **N** shows an absorption band at 408 nm while the charged analogue **1^2+^** shows a hypsochromically shifted absorption with a maximum at 315 nm and a shoulder at 335 nm. This absorption behavior is well documented in previous publications from our group for a series of analogous neutral and charged compounds17p, 19, 26 and can be attributed to a loss of the charge transfer transition from the amine to the boron moiety upon methylation of the amine. The approximate doubling of the extinction coefficient and the bathochromic shift of >40 nm (3300 cm^−1^) in absorption, when comparing the monomer **1^2+^** and the dimer **2^4+^**, is also consistent with our previous studies17p, 19 and can be attributed to a larger π‐system in the case of **2^4+^**. All photophysical data is summarized in Table 1.

**Figure 3:**
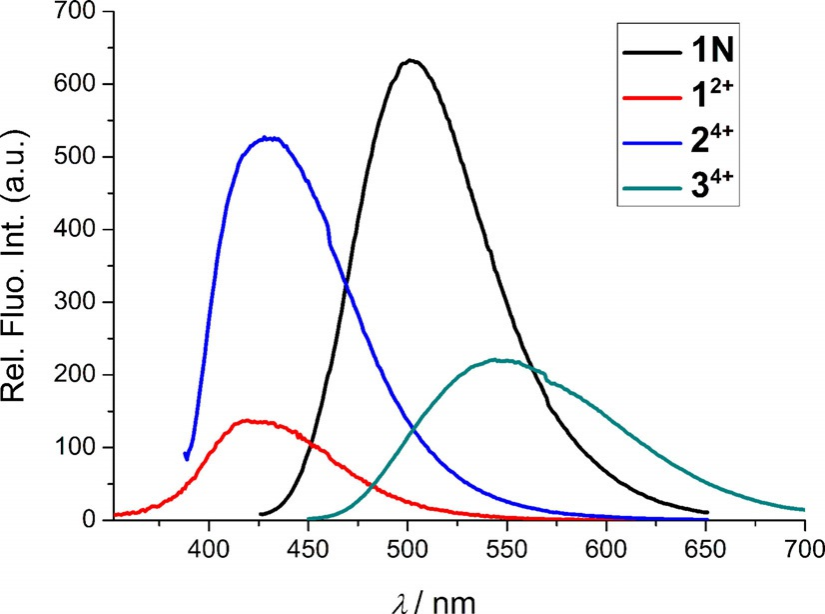
Comparison of emission spectra of neutral **1** **N** (*λ*_ex_=408 nm), **1^2+^**(*λ*_ex_ = 335 nm), **2^4+^**(*λ*_ex_ = 377 nm) and **3^4+^** ^[19]^ (*λ*_ex_ = 425 nm) in sodium cacodylate buffer (pH 7.0, *I* = 0.05 m). *c* (**1** **N** and **3^4+^**) = 5×10^−7^ m; *c* (**1^2^** ^+^ and **2^4+^**) = ×10^−8^ m.

Both the neutral and charged compounds are strongly fluorescent, with significant apparent Stoke's shifts in all tested solvents (see Table 1 and Figure 3). However, neutral compound **1** **N** showed pronounced emission quenching upon heating to 90 °C, whereas the temperature‐induced emission changes of **1^2+^** and **2^4+^** were negligible (Figures S25–S27).

### Study of interactions with DNA, RNA, and BSA

To study the interactions of **1** with DNA/RNA, several typical types of DNA and RNA were chosen (Table S2). Naturally occurring *calf thymus* (ct)‐DNA represents a typical B‐helix structure with a balanced ratio of GC‐(48 %) and AT‐(52 %) base pairs. Synthetic alternating polynucleotides poly (dGdC)_2_ and poly (dAdT)_2_ represent two extreme situations (only AT‐ or GC‐basepairs, respectively), differing significantly in their secondary structures as well as in the availability of the minor groove for small molecule binding (the guanine amino group sterically hinders deep molecule penetration). For comparison between double stranded (ds) DNA and ds‐RNA, poly rA ‐ poly rU RNA was chosen as an A‐helix structure characterized by a major groove available for the binding of bulky small molecules.

Furthermore, to explore the DNA/RNA binding of the novel chromophore to a greater extent, we also studied the single stranded synthetic ss‐RNA polynucleotides poly G, poly A, poly U and poly C, each of them characterized by different properties. Adenine ss‐RNA mimics 50 to 250 adenine nucleotides at the 3′ end of mRNA, poly G is related to guanine‐rich sequences in both DNA and RNA, whereas poly C and poly U are significantly more flexible than purine‐RNAs, and with less organized secondary structures.

Due to the possibility that the compounds studied could interact with proteins, we examined the most naturally abundant protein, bovine serum albumin (BSA), taking into account its versatility of binding sites.

### Thermal denaturation experiments

It is well known that double stranded (ds)‐helices of polynucleotides dissociate into two single stranded polynucleotides upon heating at well‐defined temperatures (*Tm* value). Non‐covalent binding of small molecules to ds‐polynucleotides usually increases the thermal stability of the ds‐helices thus resulting in increased *Tm* values, and the increase (Δ*Tm*) can (corroborated by other methods) be related to the various binding modes.^27^

Tetra‐charged **2^4+^** stabilized ds‐DNA moderately (*r*_[compound]/[polynucleotide]_=0.1; Δ*Tm*=+4.0 °C), whereas monomer **1^2+^** induced only minor stabilization (*r*_[compound]/[polynucleotide]_=0.1; Δ*Tm*=+1.1 °C) and the neutral analogue **1** **N** did not affect the thermal stability of ds‐DNA (Figures S29–S31). The results show a direct proportionality of ds‐DNA stabilization to the number of positive charges. However, tetra‐cation **3^4+^** stabilized ds‐DNA much more strongly (*r*_[compound]/[polynucleotide]_=0.1; Δ*Tm*=+7.3 °C), pointing to the significant impact of the bithiophene linker.^20^

### Fluorimetric titrations with DNA, RNA, BSA

**Figure 4:**
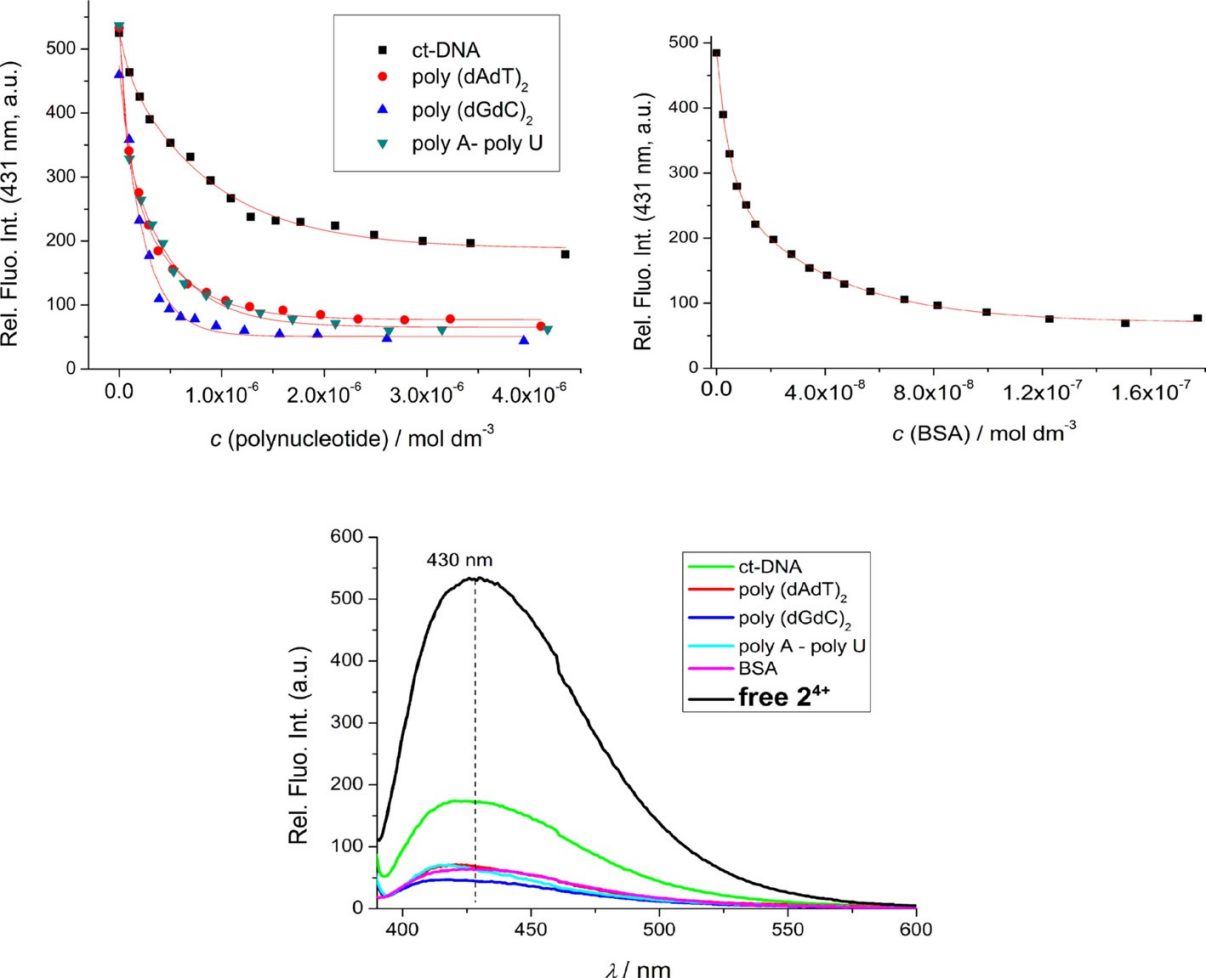
Top: Comparison of fluorimetric titrations of **2^4+^** (*c =* 5×10^−8^ m, *λ*_ex_ = 377 nm) at *λ*_em_ = 431 nm for all ds‐DNA, ds‐RNA and BSA (ss‐RNA not shown, see Supporting Information Figures S38–S42). Bottom: Comparison of the fluorescence spectrum of **2^4+^** with spectra of **2^4+^**/biomacromolecule complexes at the end of titrations. All measurements performed at pH 7.0, in sodium cacodylate buffer, *I* = 0.05 M.

The strong intrinsic fluorescence of the compounds studied allowed fluorimetric titrations with various ds‐DNA/RNA, single‐stranded (ss)‐RNA and BSA. Addition of ds‐DNA or ds‐RNA caused strong, non‐selective quenching of the emission of **1^2+^** (Figures S34 & S35) or **2^4+^** (Figure 4), but no fluorescence change was observed for neutral **1** **N** (Figure S32). Intriguingly, addition of ss‐RNA or BSA caused negligible changes in the emission from monomer **1^2+^** (Figures S36 & S37), in contrast to the behavior of dimer **2^4+^**, which revealed a strong fluorimetric response (Figures S38–S42).

Furthermore, **3^4+^** showed an emission increase upon DNA/RNA/BSA complexation, characterized by distinct differences in fluorescence maxima between DNA/RNA and protein (BSA), whereas **2^4+^** showed only non‐selective emission quenching. Such a difference in fluorimetric response between **2^4+^** and **3^4+^** might be attributed to the fact that bithiophene is a weak donor, whereas the diyne unit does not have an electron‐donating effect.

The non‐linear fitting of the DNA or RNA titration data by means of the Scatchard equation (McGhee, vonHippel formalism)^28^, ^29^ allowed the calculation of binding constants (Table 2). The BSA titration data fitted excellently to a 1:1 (**2^4+^**:BSA) stoichiometry model, pointing to only one dominant binding site of **2^4+^** at BSA.

**Table 2:** Binding constants (log *Ks*)^[a]^ of **1^2+^** and **2^4+^** with polynucleotides or BSA calculated by processing fluorimetric titrations;^[a]^ at pH = 7.0, sodium cacodylate buffer, *I* = 0.05 M.

Polynucleotide	**2^4+^**	**1^2+^**
ct‐DNA	6.9	6.0
poly dAdT‐ poly dAdT	7.7	–
poly dGdC ‐poly dGdC	7.8	–
poly A ‐ poly U	7.6	6.0
poly G	7.5	–
poly A	7.2	^[c]^
poly C	7.4	–
poly U	6.7	–
BSA^[b]^	8.2^[b]^	^[c]^

[a] Analyses of titration data by means of the Scatchard equation^28^, ^29^ gave values of the ratio *n* [bound **2^4+^**]/[polynucleotide]=0.2−0.5; for easier comparison, all log *Ks* values were re‐calculated for fixed *n*=0.25 (ds‐polynucleotides) and *n*=0.5 (ss‐RNA). Correlation coefficients were >0.99 for all calculated *Ks* values. [b] Fitted for Langmuir isotherm for **2^4+^**:BSA 1:1 stoichiometry. [c] Negligible emission change did not allow analysis of titration data.

The strong, submicromolar affinities of **2^4+^** to ds‐DNA and ds‐RNA were within the same order of magnitude, while its affinity to ss‐RNA was approximately an order of magnitude lower. The affinity of **2^4+^** to BSA was an order of magnitude higher than its affinity towards ds‐DNA/RNA (Table 2). The excellent fit of the titration data (Figure S42) strongly supported a single dominant binding site on BSA for **2^4+^**, although other binding sites with several orders of magnitude lower affinities cannot be excluded. Comparison of the affinities between **2^4+^** and bithiophene analogue **3^4+^** revealed similar binding constants for ds‐DNA/RNA and ss‐RNA, but a significant difference in binding to BSA, with **2^4+^** showing an affinity two orders of magnitude higher than **3^4+^**.

Monomer **1^2+^** revealed somewhat lower affinity to ds‐DNA/RNA, but still in the micromolar range, albeit having only half of the positive charge, suggesting that electrostatic interactions with the negatively charged DNA backbone are not the dominant binding interactions. Intriguingly, the emission of **1^2+^** did not change with BSA or ss‐RNA addition, indicating that the long rod‐like structure of dimer **2^4+^** is essential for efficient binding to both targets.

### CD experiments

Thus far, we had studied the non‐covalent interactions at 25 °C by monitoring the spectroscopic properties of the compounds upon addition of the polynucleotides. In order to obtain insight into the changes of polynucleotide properties induced by small molecule binding, we chose CD spectroscopy as a highly sensitive method for the examination of conformational changes in the secondary structure of polynucleotides.^30^ In addition, **1^2+^** or **2^4+^** as achiral small molecules could display induced circular dichroism (ICD) within their absorption spectra upon binding to polynucleotides, which could provide useful information about modes of interaction.^31^, ^32^

**Figure 5:**
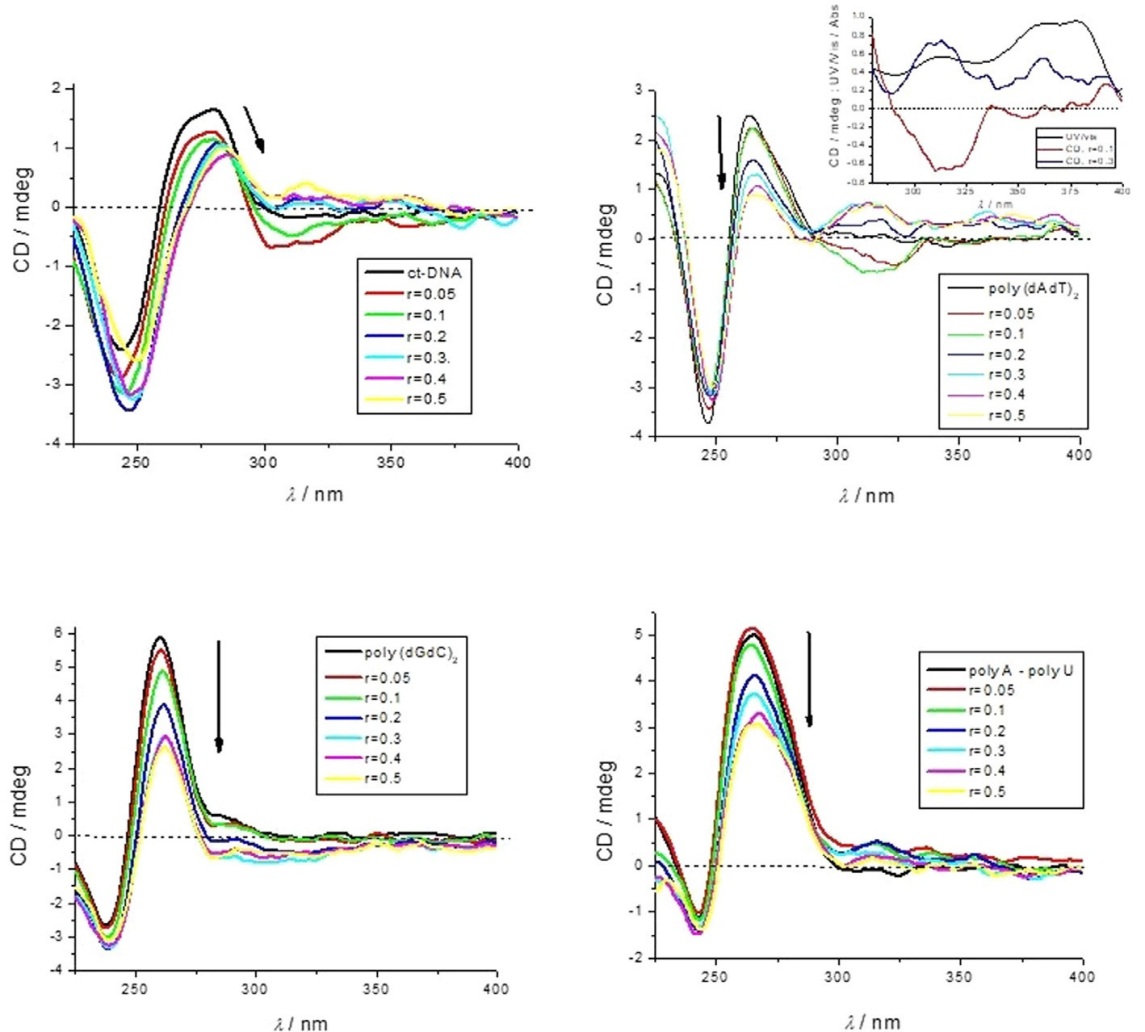
CD titration of ct‐DNA, poly (dAdT)_2,_ poly (dGdC)_2_, poly A‐poly U (all DNA/RNA *c =* 2×10^−5^ m) with **2^4+^** at molar ratios *r* = [**2^4+^**]/[polynucleotide] (pH 7.0, buffer sodium cacodylate, *I* = 0.05 m).

Addition of **1^2+^** did not significantly change the CD spectra of ds‐DNA or ds‐RNA (Figures S44–S46), and no induced (I)CD bands >300 nm were observed. In contrast, tetracation **2^4+^** induced a significant decrease in intensity in the CD spectra of all ds‐DNA/RNA (230–300 nm range; Figure 5), attributed to a pronounced decrease in ds‐polynucleotide chirality.^31^, ^32^

Only poly (dAdT)_2_ (Figure 5) revealed significant induced (I)CD bands at *λ*>300 nm, which could be attributed to the uniformly oriented binding of **2^4+^** within a well‐defined DNA binding site.^32^ Taking into account the structure of the molecule **2^4+^**, the DNA minor groove is the most plausible binding site. Closer inspection of the ICD bands and comparison with the UV/Vis titration data (Figure 5, Inset: black line) revealed a mixed binding mode of **2^4+^**, dependent on a ratio *r*_[compound]/[poly (dAdT)2]_; whereby for *r*<0.2 ICD bands were negative and for *r* ≥0.3 ICD bands were positive. Such a change of the ICD sign is commonly attributed to single molecule binding at an excess of DNA (*r*<0.2) and molecular aggregation within the DNA grooves at an excess of the small molecule (*r*>0.3).^31^, ^32^

Further, dominant ICD bands at 300–330 nm could be partially attributed to electronic transition vectors along the boron‐nitrogen axes (see Table S5), which seem to be well oriented with respect to the DNA chiral axis. The maxima at *λ*_max_=360–380 nm), giving negligible ICD band, were attributed to the electronic transition along the long axis of **2^4+^**, parallel to the diyne‐linker (see Table S5).

The almost negligible intensity of ICD bands for the analogue **2^4+^**/GC‐DNA complex could be attributed to the sterically crowded minor groove with the amino groups of guanine, not allowing deep insertion of **2^4+^** and, thus, diminishing the induced CD effect.^32^ The broad and shallow minor groove of AU‐RNA is a poor binding site for small molecules, in contrast to the major groove of RNA, which has a width similar to that of the minor groove of DNA (Table S2) and could be an efficient binding site for **2^4+^**. However, the large depth of the major groove allows heterogeneous orientation of **2^4+^** molecules with respect to the ds‐RNA chiral axis, thus resulting in negligible ICD bands.

**Figure 6:**
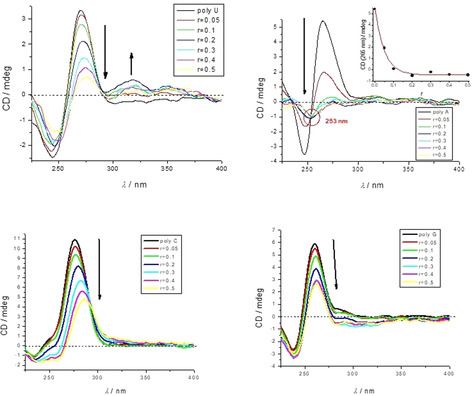
CD titration of poly U, poly A, poly C and poly G (all RNA *c =* 2×10^−5^ m) with **2^4+^** at molar ratios *r* = [**2^4+^**]/[polynucleotide] (pH 7.0, buffer sodium cacodylate, *I* = 0.05 m).

Particularly intriguing results were obtained for the **2^4+^**/ss‐RNA complexes (Figure 6). Addition of **2^4+^** completely disordered the CD spectrum of poly A and also that of poly U, while the CD spectra of poly G and poly C were less affected. For the poly A titration, the isoelliptic point at *λ*=253 nm strongly supported only one type of **2^4+^**/poly A complex. The poly U titration showed a systematic shift of the spectral crossing points during titration, which is typical for mixed binding modes.

Complete loss of the helical chirality of A or U ss‐RNA upon binding to **2^4+^**, accompanied with a rather high affinity of **2^4+^** (Table 2) suggested wrapping of the ss‐polynucleotide chain around the cylindrically shaped compound **2^4+^**. Such a binding mode would maximize the efficiency of electrostatic interactions between four positive charges of **2^4+^** and the negative polynucleotide backbone. This binding mode would be additionally supported by an energetically favorable exclusion of the hydrophobic diyne linker from hydrophilic solvent molecules. Such a complex of achiral **2^4+^** serving as a spindle for ss‐RNA would not give any chiral response, which is in accordance with the CD titration experiments (Figure 6).

Another proof of the proposed binding mode is that monomer **1^2+^** did not change the CD spectrum of ss‐RNA, which could be attributed to the globular shape of **1^2+^** with its centered positive charge, not supporting ss‐polynucleotide wrap‐around.

### Raman spectroscopy

**Figure 7:**
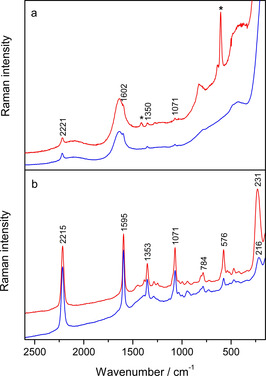
a) Raman spectra of **2^4+^** (*c =* 1×10^−4^ m) in water (blue line) and Na‐cacodylate buffer, pH 7.0 (red line). b) SERS spectra of **2^4+^** (*c =* 1×10^−6^ m), in the silver colloid not containing Na‐cacodylate buffer (blue line) and containing Na‐cacodylate buffer, pH 7.0 (red line). λ_ex_ = 758 nm. The bands labelled with asterisks originate from the buffer. The spectra are displaced for visual clarity.

Raman spectra of **2^4+^** were measured in water (1×10^−4^ m) and Na‐cacodylate buffer, (pH 7.0, 1×10^−4^ m and 5×10^−5^ m) (Figure 7 and Table S4). In the Raman spectra of all solutions, a broad band around 3220 cm^−1^ and a medium band around 1640 cm^−1^ were observed and assigned to the water stretching and bending modes, respectively. In addition, in the Raman spectra of the buffered solutions, bands originating from cacodylate ions were obtained: a medium methyl asymmetric stretching band at 2935 cm^−1^, a weak methyl deformation band at 1414 cm^−1^ and an intense As=O stretching band at 605 cm^−1^. Nevertheless, bands distinctive of **2^4+^** were observed in all Raman spectra, even at a concentration as low as 5×10^−5^ m (Table S4). Hence, the band around 2220 cm^−1^ was assigned to the stretching of the C≡C triple bonds in bisaryl‐substituted diynes,^6^ while the band around 1600 cm^−1^, overlapped by the water band, was attributed to a phenyl ring stretching mode (Table S4).^33^ Based on the calculated Raman spectrum of **2^4+^** (Figure S49), the band around 1355 cm^−1^ was associated with mixed stretching vibrations of the triple bonds and phenyl rings, whereas stretching of the bonds between the boron atom and three aromatic substituents contributed to the band around 1070 cm^−1^. The strong Raman scattering of **2^4+^** was attributed to the diyne moiety conjugated to the aromatic rings,^34^ allowing detection of the molecules in solution at a micromolar concentration range.

### SERS experiments

**Figure 8:**
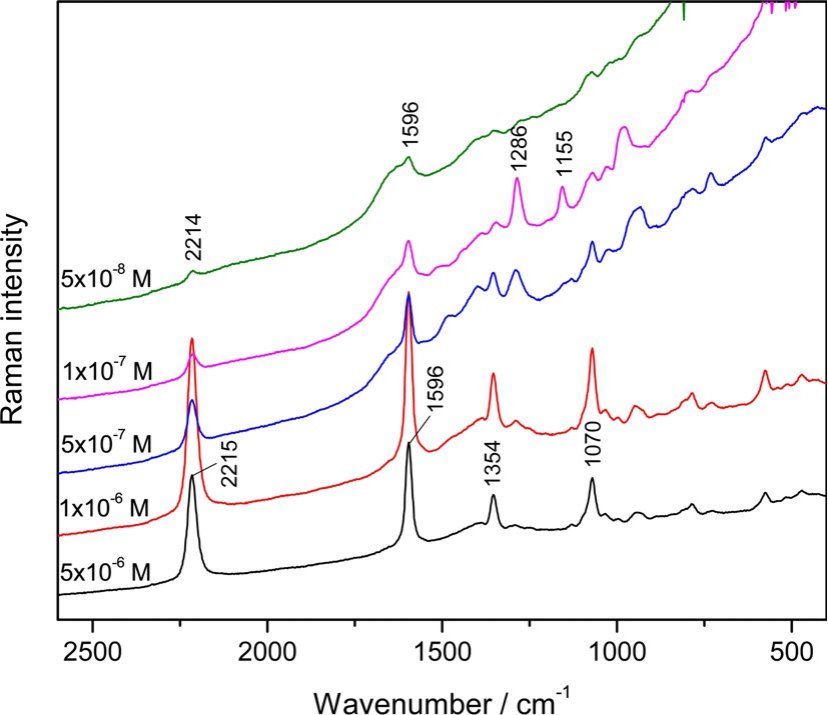
The concentration‐dependent SERS spectra of **2^4+^** in the silver colloid not containing Na‐cacodylate buffer, *c =* 5×10^−8^−5×10^−6^ M λ_ex_ = 758 nm. The spectra are displaced for visual clarity.

The SERS spectra of **2^4+^** were measured in the 5×10^−8^ ‐ 5×10^−6^ m concentration range (Figure 8). The characteristic SERS bands of **2^4+^** were obtained at a concentration as low as 5×10^−8^ m, whereas the Raman scattering enhancement was the highest for the 1×10^−6^ m sample. Referring to the Raman bands assignment, the strong band at 2215 cm^−1^ was assigned to the stretching of the C≡C triple bonds, while the intense band at 1596 cm^−1^ was attributed to the stretching of the phenyl moieties. The medium band at 1354 cm^−1^ was assigned to the stretching modes of the central conjugated part of the molecule, including phenyl rings and triple bonds, whereas the moderate band at 1070 cm^−1^ was associated with the stretching of the bonds between the boron atom and aromatic substituents. Aside from the few **2^4+^** bands observed in the Raman spectra, some additional bands were obtained in the SERS spectra, corresponding mainly to phenyl ring vibrations (Table S4). The bands at 1286 cm^−1^ and 1155 cm^−1^ which appeared at low **2^4+^** concentrations (1×10^−7^ m and 5×10^−7^ m) were attributed to the symmetric stretchings of ‐CF_3_ and ‐SO_3_ groups, respectively, of trifluoromethanesulfonate anions.^35^ At low concentrations of **2^4+^**, the counterions came close to the enhancing silver surface and, consequently, their vibrational modes were enhanced.

**Scheme 3:**
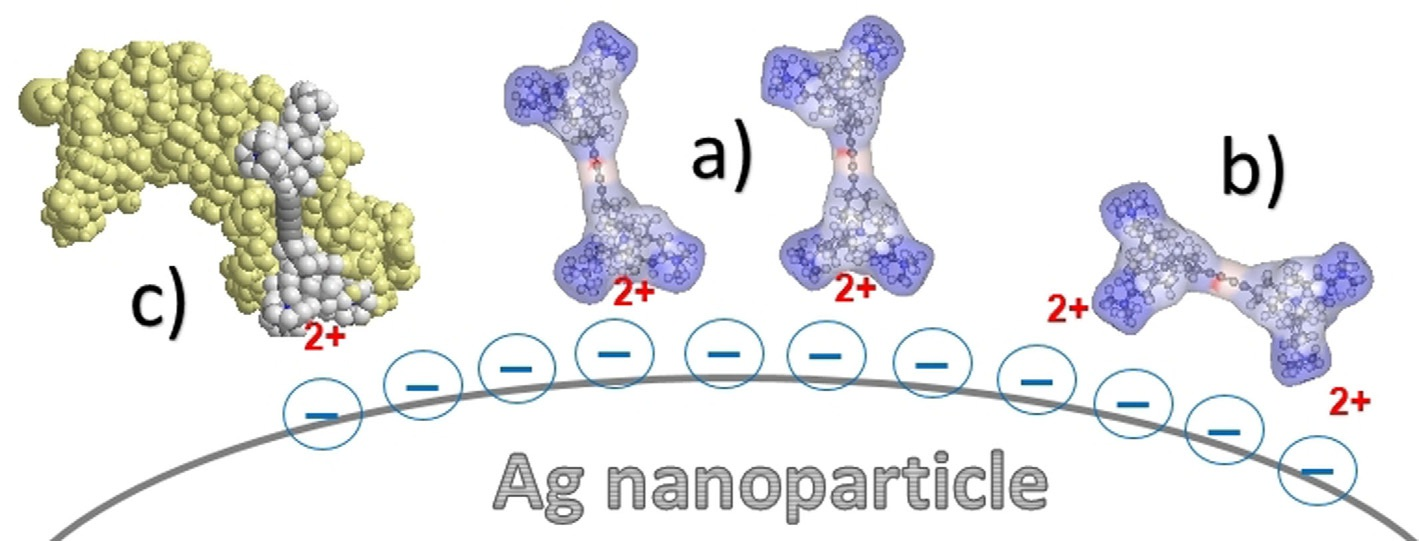
SERS experiments: Depiction of the molecular orientation of compound **2^4+^** with respect to the silver surface at high concentrations of **2^4+^** (a), low concentrations of **2^4+^** (b), and in the complex with ds‐DNA (c). The **2^4+^**/BSA complex (not shown) is detached from the Ag‐surface and, thus, gives no SERS signal.

Considering the surface selection rules according to which polarizability changes perpendicular to the metal surface contribute the most to scattered radiation, the prominent C≡C (2215 cm^−1^) and phenyl (1596 cm^−1^) stretching bands implied that, at a concentration of 1×10^−6^ m, the molecules adopted an optimal position with the triple bonds and phenyl rings oriented perpendicular to the silver surface (Scheme 3 a).

Moreover, the positive charge on each side of the molecule facilitated its positioning in hot spots between two nanoparticles. It can be assumed that adsorption of the **2^4+^** molecules was electrostatically driven by the positively charged trimethylamino groups attracted to the citrate anions on the silver nanoparticles, followed by direct interactions with the silver surface. A band observed in the low wavenumber region (216 cm^−1^) was assigned to Ag−N stretching, indicating interaction between the nitrogen atom and the silver surface (Figure 7 b). By decreasing the concentration, the intensity of the characteristic vibrational bands diminished, most likely due to first tilted and then in‐plane positioning of the molecules on the enhancing surface (Scheme 3 b).

In order to investigate the effect of the buffer on the SERS response, the spectrum of **2^4+^** (1×10^−6^ m) in Na‐cacodylate buffer (pH 7.0) was acquired (Figure 7 b). In general, the spectrum was slightly more intense and the bands more defined, when compared to the spectrum of **2^4+^** in water, though positively charged **2^4+^** molecules most likely acted as aggregating agents of the silver colloid. Salts in the buffer composition additionally induced aggregation of the silver nanoparticles resulting in stronger enhancement of the Raman scattering. A strong band observed at 231 cm^−1^ was assigned to the stretching of the Ag−Cl bond formed between the chloride ions from the buffer and the silver surface (Figure 7 b).

**Figure 9:**
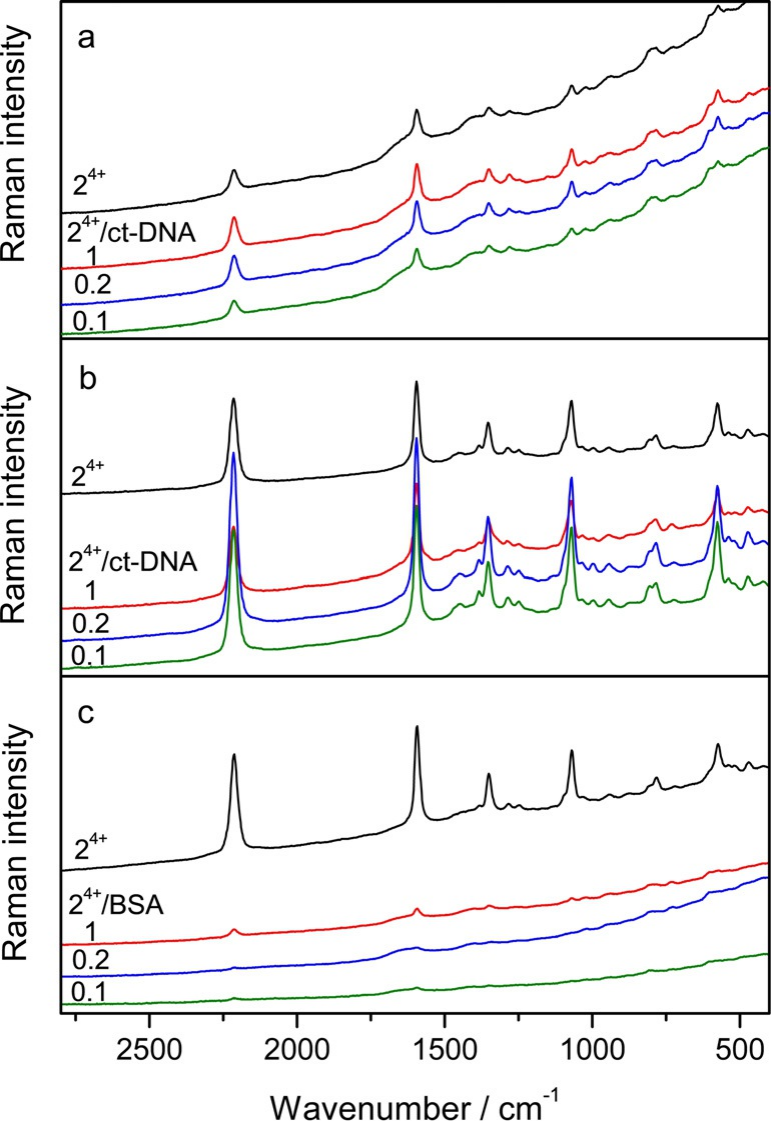
a) SERS spectra of **2^4+^** and **2^4+^**/ct‐DNA complexes of ratio *r*[**2^4+^**]/[ct‐DNA] = 1, 0.2 and 0.1, in the silver colloid containing Na‐cacodylate buffer, pH 7.0; *c*(**2^4+^**) = 5×10^−8^ m b) SERS spectra of **2^4+^** and **2^4+^**/ct‐DNA complexes of ratio *r* = 1, 0.2 and 0.1, in the silver colloid containing Na‐cacodylate buffer, pH 7.0; *c*(**2^4+^**) = 1×10^−6^ m c) SERS spectra of **2^4+^** and **2^4+^**/BSA complexes of ratio *r* = 1, 0.2 and 0.1, in the silver colloid containing Na‐cacodylate buffer, pH 7.0; *c*(**2^4+^**) = 1×10^−6^ m λ_ex_ = 758 nm. The spectra are displaced for visual clarity.

To study the binding of **2^4+^** to ds‐DNA, the SERS spectra of the **2^4+^**/ct‐DNA complexes in *r*[**2^4+^**]/[ct‐DNA] molar ratios of 1, 0.2 and 0.1 were measured at two different **2^4+^** concentrations: a) *c*(**2^4+^**)=1×10^−6^ m, which produced the strongest SERS response, and b) at the lowest concentration at which **2^4+^** was detected (5×10^−8^ m) (Figure 9 a,b). In both cases, the SERS spectra obtained correspond to the SERS spectrum of the small molecule.

The SERS bands were more intense for the **2^4+^**/ct‐DNA complex than for **2^4+^** alone. For example, at *c*(**2^4+^**)=5×10^−8^ m, the intensity of the triple C≡C stretching band at 2214 cm^−1^ for the complexes of *r*[**2^4+^**]/[ct‐DNA]=1 and 0.2 was enhanced 1.4 and 1.3 times, respectively, relative to that of the free **2^4+^** molecules. The increase in intensity obtained indicated interactions of **2^4+^** with the nucleic acid, owing to which the small molecules adopted a more optimal orientation with respect to the enhancing silver surface and/or were placed closer to the silver nanoparticles (Scheme 3 c). On the other hand, the excess of highly negatively charged DNA in the **2^4+^**/ct‐DNA sample of the *r*=0.1 most likely caused less efficient adsorption of the complex on the silver surface, reducing the SERS intensity.

Furthermore, the SERS response of **2^4+^** (*c=*1×10^−6^ m) upon binding to BSA was studied for the **2^4+^**/BSA complexes prepared in *r*[**2^4+^**]/[BSA] molar ratios of 1, 0.2 and 0.1 (Figure 9 c). Unlike the complexes of **2^4+^** with the nucleic acids, the SERS intensity significantly diminished regardless of the complex molar composition. The characteristic band of the triple bond stretching at 2215 cm^−1^ almost completely vanished from the spectrum of the **2^4+^**/BSA complex of the molar ratio 0.1. Due to strong interactions of the small molecules with the protein, and likely deep insertion of the molecule within the BSA binding site, compound **2^4+^** was removed from the silver nanoparticles responsible for the SERS effect.

It is interesting to note that the characteristic SERS response of **2^4+^** was observed upon NIR excitation (785 nm) in the simply prepared and widely used silver colloid, even at a nanomolar concentration range. Thereby the distinctive band of the C≡C triple bond at 2215 cm^−1^, which does not interfere either with the bands of its own, or with the bands of the other species, in the measured system, allowed easy detection of the small molecules. The intense SERS response obtained for **2^4+^** interacting with DNA, and its loss upon binding with BSA, is characteristic of binding with the nucleic acid and the protein, respectively. Owing to the sensitivity obtained and minimal spectral interference, the molecules studied could potentially be used as alkyne‐coded SERS tags for live cell imaging.2d

### Preliminary biological screening

The biological experiments aimed to verify the actual capability of the DNA/RNA/protein binder studied to penetrate the cells, to visualize its intracellular location and subcellular targets. Evaluation of the anti‐proliferative effect was conducted in order to identify further potential applications, either as a cytotoxic lead compound toward theranostic^36^ applications (combining dual fluorescent/Raman monitoring with an anti‐proliferative action), or as a non‐cytotoxic dye suitable for intracellular applications or in biochemical studies.

To examine toxicity, i.e., the effect of **2^4+^** on the proliferation of human cell lines, we used the MTT test. The compound was tested on two human cell lines (HeLa and HEK 293). As an indicator of anti‐proliferation activity we used the IC_50_ value, which corresponds to the concentration of the compound that inhibits proliferation to 50 % compared to the control cells (proliferating without the compound in the medium). The results we obtained showed negligible anti‐proliferative activity of **2^4+^** even at the highest concentration used in the test (1×10^−4^ m, results not shown).

**Figure 10:**
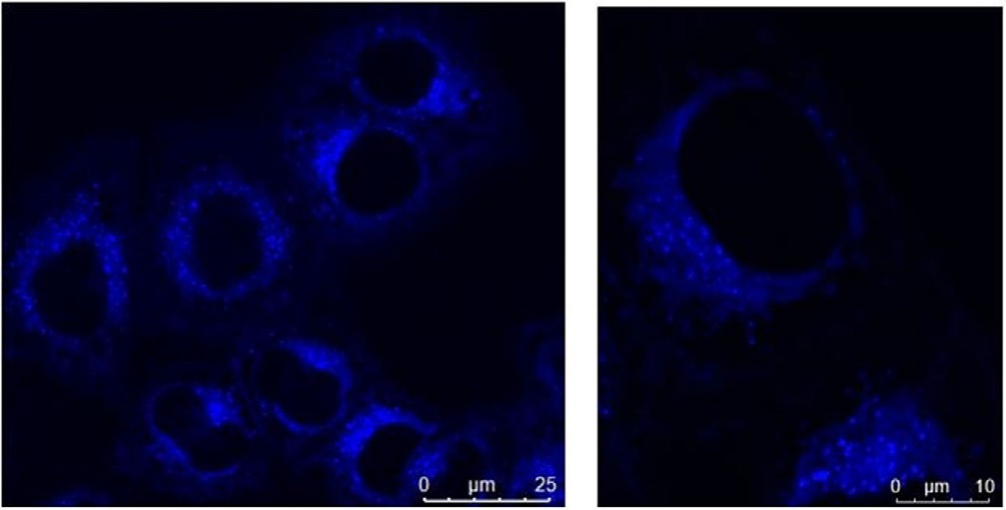
Confocal microscopy of live HeLa cells (*c =* 50 000 cells mL^−1^) taken on a Leica SP8 X confocal microscope, cells stained for 2 h in the *c*(**2^4+^**) = 1×10^−6^ m, λ_exc_ = 360–400 nm; λ_em_ = 415–471 nm.

We also checked the ability of the compound to cross the cellular membrane and penetrate the HeLa cells, by using the intrinsic fluorescence of **2^4+^** in fluorescent confocal microscopy experiments. Confocal microscopy also allows us to propose the possible intracellular localization of the compound. The compound **2^4+^** entered cells very efficiently within 2 hours of cell immersion in *c*(**2^4+^**)=1×10^−6^ m. The observed fluorescence of the compound coincided with the area of the cell where the endoplasmic reticulum is located, and the grainy dispersion of the fluorescence signal indicates possible endosomal accumulation (Figure 10).

As titration experiments of **2^4+^** with DNA/RNA and BSA revealed strong quenching of the emission of **2^4+^** upon binding, the strong fluorescence in the cell suggests that emission from the compound is not significantly affected by its proposed intracellular localization. This would be consistent with the hydrophobic endosomal environment, which is devoid of any DNA/RNA or albumin‐like binding sites. This could spur intriguing future studies of **2^4+^** and its analogues in dual fluorescence/Raman‐based SRS microscopy,2d whereby the intracellular location with quenched fluorescence and active SRS signal could be easily differentiated from the intracellular location with strong fluorescence.

The cell nucleus is completely void of any emission which, in combination with its negligible anti‐proliferative activity, strongly suggests that **2^4+^** does not bind to or interfere with genomic DNA or RNA processes, which are essential for cell viability. However, for a more accurate determination of its cellular localization, further, more detailed biological experiments are planned.

## Conclusions

As a follow‐up to our work on novel applications of bis‐triarylboranes as DNA/RNA/protein binders and bright intra‐cellular probes,17p, 19, 20, 26 we designed and studied new derivatives to investigate the influence of the linker type on DNA/RNA/protein interactions and to compare (4‐Ar_2_B‐3,5‐Me_2_C_6_H_2_)‐C≡C−C≡C‐(3,5‐Me_2_C_6_H_2_‐4‐BAr_2_ (**2^4+^**) with its corresponding monomeric (4‐Ar_2_B‐3,5‐Me_2_C_6_H_2_)‐C≡CH (**1^4+^**) analogue.

It can be safely assumed that the positively charged analogue **2^4+^** retains structural features very similar to those of its neutral analogue **2** **N** (see Figure 1); thus, both compounds could be considered as rod‐like dumbbell structures. Compound **2^4+^** is also characterized by four terminal positive charges. Only **2^4+^** was soluble in water and, therefore, further studied for biorelevant applications using, as a reference, the corresponding monomer **1^2+^**.

One of the main aims of the **2^4+^** design was to study the impact of the linker connecting two triarylborane units upon the binding to DNA/RNA or protein, in comparison to previously studied bithiophene‐linker analogue **3^4+^**.^20^ Addition of any type of DNA/RNA/protein induced quenching of both **2^4+^** and **1^2+^** fluorescence, in contrast to the strong emission enhancement of **3^4+^**. Such an opposite effect demonstrates the pronounced impact of the linker on the triarylborane fluorescence emission. Furthermore, globular‐shaped monomer **1^2+^** showed only negligible interaction with protein BSA, stressing that the highly hydrophobic linker in “dimers” (**2^4+^** or **3^4+^**) is essential for efficient binding to the hydrophobic pocket of BSA. Moreover, the linker structure (**2^4+^** diyne vs. **3^4+^** bithiophene) strongly influenced the BSA affinity (**2^4+^** log*Ks*=8.2 vs. **3^4+^** log*Ks*=5.9), suggesting that a linear, aliphatic low‐volume linker (**2^4+^** diyne) is highly preferred for insertion into BSA binding site.

Both cationic compounds (**2^4+^**, **1^2+^**) showed remarkably high affinity toward various types of DNA/RNA (log*Ks*=6–7.5 range) and, intriguingly, dicationic monomer **1^2+^** showed only an order of magnitude lower affinity than tetracationic **2^4+^**. This implies only a minor contribution of electrostatic interactions with the negatively charged DNA/RNA backbone and suggested binding within ds‐DNA/RNA grooves, within which the highly hydrophobic linker and extensive interactions of aromatic units surrounding the boron atoms strongly contribute to overall DNA/RNA affinity.

However, tetracationic **2^4+^** also strongly interacted with ss‐RNA, which does not possess any groove as a binding site. The CD results strongly supported ss‐RNA chain wrapping around tetracationic **2^4+^** as a thread around the spindle, which is a very unusual mode of binding of ss‐RNA with small molecules. The absence of interaction of ss‐RNA with globular‐shaped structure of **1^2+^** additionally stressed the nature of the rod‐like dumbbell structure of **2^4+^** being crucial for strong interaction with ss‐RNA.

Another major design feature of **2^4+^** was the ability to use the diyne‐linker as a Raman‐probe, complementing the **2^4+^** fluorescence response. Thus, in aqueous solution **2^4+^** gave rise to several remarkably strong Raman bands in the 2220–1355 cm^−1^ range, allowing accurate monitoring at as low as 10 micromolar concentration. Even more interesting was the response of **2^4+^** in the SERS spectra, leading to a detection limit below 10‐nanomolar concentrations. Most intriguingly, addition of DNA actually increased the SERS signal intensity slightly, whereas BSA completely quenched it. This specific response is very useful and complementary to that of the **2^4+^** fluorescence response in which DNA/RNA and BSA cause similar emission quenching.

Finally, preliminary biological activity screening showed that **2^4+^** entered human cells very efficiently while not interfering with cell viability up to 10^−4^ m concentrations. Its bright fluorescence is proposed to originate from localization along the endoplasmic reticulum, possibly via intra‐ribosomal or endosomal accumulation.

Our results strongly support further development of bis‐triarylborane Raman/fluorescence chromophores as dual probes for simultaneous confocal fluorescence microscopy and SRS microscopy of cell lines, whereby careful choice of a linker can finely tune DNA‐protein selectivity (note the difference between **2^4+^** diyne vs. **3^4+^** bithiophene) and consequently the intracellular accumulation of a probe. The Raman‐response of **2^4+^**‐analogues in SRS microscopy could, thus, efficiently complement the fluorescence response when the studied system is turbid/non‐suitable for fluorescence imaging (e.g. cell‐organoid agglomerates), some other fluorescence probe is used simultaneously, or one of the cellular targets quenches the fluorescence emission. Moreover, the unique binding mode of ss‐RNAs with very high affinity makes bis‐triarylborane tetracations very promising ss‐RNA delivery systems, particularly as some of them have already shown very efficient cellular uptake and negligible toxicity.17p, 19, 26

## Conflict of interest

The authors declare no conflict of interest.

## Supporting information

As a service to our authors and readers, this journal provides supporting information supplied by the authors. Such materials are peer reviewed and may be re‐organized for online delivery, but are not copy‐edited or typeset. Technical support issues arising from supporting information (other than missing files) should be addressed to the authors.

Supplementary
