# Supplementary material for: Tetracationic Bis‐Triarylborane 1,3‐Butadiyne as a Combined Fluorimetric and Raman Probe for Simultaneous and Selective Sensing of Various DNA, RNA, and Proteins
Source: Chemistry. 2020 Apr 24;26(27):6017–28. doi: 10.1002/chem.201905328 (PMC7318631; doi:10.1002/chem.201905328)
Supplement: Supplementary file 1 — Supplementary [file CHEM-26-6017-s001.pdf]

# Chemistry–A European Journal

Supporting Information


## Tetracationic Bis-Triarylborane 1,3-Butadiyne as a Combined Fluorimetric and Raman Probe for Simultaneous and Selective Sensing of Various DNA, RNA, and Proteins


Hashem Amini,[a] Željka Ban,[b] Matthias Ferger,[a] Sabine Lorenzen,[a] Florian Rauch,[a] Alexandra Friedrich,[a] Ivo Crnolatac,[b] Adriana Kenđel,[c] Snežana Miljanić,[c] Ivo Piantanida,*[b] and Todd B. Marder*[a]

# Supporting information

**Contents**

**Synthesis and NMR data..........................................................................................S3**

**Single-crystal X-ray diffraction.......................................................................S19**

**Photophysical measurements in solution........................................................S22**

**Study of interactions with DNA/RNA/protein...................................................S32**

**Raman and SERS measurements…………………………………………………...S46**

**Biological Screening…………………………………………………………………..S49**

**Theoretical Studies…………………………………………………………………….S50**

**References……………………………………………………………………………….S57**

**Synthesis: General information**

Unless otherwise stated, all reactions were performed under a dry argon atmosphere. All workups were carried out in air. $NEt_3$, THF, *n*-hexane and $CH_2Cl_2$ were deoxygenated and then dried using an Innovative Technology Inc. Pure Solvent Purification System. Bis[4-(*N*,*N*-dimethylamino)-2,6-dimethylphenyl]fluoroboran[1] was synthesized according to literature. Trimethylsilylacetylene (97%, Fluorochem), *tert*-butyllithium (1.7 M in pentane, Sigma-Aldrich), methyl trifluoromethanesulfonate (98%, Sigma-Aldrich), CuI (98%, Alfa Aesar), and alumina ($Al_2O_3$, neutral, Brockmann I, for chromatography, 50-200 µm, Acros) were used as received. Other solvents and chemicals were purchased from commercial sources and were used without further purification.

All solution NMR spectra were acquired at r.t. using Bruker Avance III HD 300 or Bruker Avance 500 NMR spectrometers (the 500 MHz spectrometer is equipped with a cryoprobe). All chemical shifts are given in ppm and are referenced via residual solvent peaks for $^{1}$H NMR (5.32 ppm for $CD_2Cl_2$, 3.31 ppm for $CD_3OD$ and 7.26 for $CDCl_3$) and $^{13}C\{^{1}H\}$NMR (53.84 ppm for $CD_2Cl_2$ and 49.00 ppm for $CD_3OD$). Whereas $^{19}$F chemical shifts are referenced to external $CFCl_3$ and $^{11}B\{^{1}H\}$ signals are referenced to external $BF_3{\cdot}OEt_2$. The solid-state magic-angle spinning (MAS) NMR spectra were recorded using a Bruker DSX-400 spectrometer and a 4 mm rotor (o. d.) and are referenced to external $BF_3{\cdot}OEt_2$. Isotropic chemical shifts were estimated by simulating the observed spectrum using the Solid Line Shape Analysis 2.2.4 (SOLA) in Bruker TopSpin.

Elemental analyses were carried out using an Elementar vario MICRO cube elemental analyzer. Carbon analyses of **1N** and $\mathbf{2^{4+}}$ were respectively 0.6% and 2.0% less than the calculated values. The lower carbon value in elemental analysis of organo-$BMes_2$ compounds has been reported previously and may be due to formation of boron carbide during the elemental analysis measurement.[2]

High resolution mass spectra were measured with a Thermo Fisher Scientific Exactive Plus Orbitrap MS System. Thin-layer chromatography (TLC) was carried out on neutral aluminium oxide.

**Synthesis of 2,5-dibromo-*m*-xylene (A)**

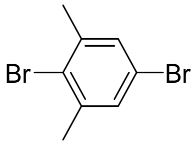

A Schlenk flask was charged with 2-bromo-1,3-dimethylbenzene (13.5 g, 72.9 mmol), $B_2pin_2$ (20.0 g, 78.7 mmol), dtbpy (199 mg, 0.73 mmol), $[Ir(COD)(OMe)]_2$ (241 mg, 0.37 mmol) and *n*-hexane (100 mL). The mixture was stirred over night at 80 °C. The following steps were performed open to air. The reaction mixture was transferred into a round bottom flask. The solvent was removed by rotary evaporation. Methanol (400 mL) and a solution of $CuBr_2$ (48.8 g, 219 mmol) in water (400 mL) were added and the mixture was stirred for 5 d under reflux. The mixture was cooled to 3°C. The precipitated solid was collected by filtration. *n*-Hexane was added and the insoluble residue was separated by passing the solution through a silica-gel pad. The solvent was removed by rotary evaporation to give **A** as a colorless liquid that crystallised over night at r.t. (17.5 g, 66.3 mmol, 91%). The spectroscopic data matched those reported previously.[3]

**NMR** (δ (ppm), $CDCl_3$): $^{1}$**H** (300 MHz) 7.21 (br s, 2H), 2.38 (m, 6H)

**Synthesis of 2-bromo-5-(trimethylsilyl)ethynyl-*m*-xylene (B)**

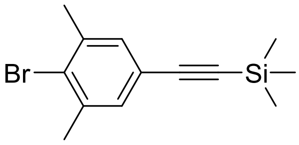

This compound was synthesized according to a reported procedure for a related compound.[4] A Schlenk flask was charged with CuI (549 mg, 2.88 mmol), $Pd(PPh_3)_2Cl_2$ (717 mg, 1.02 mmol) and 350 mL of degassed $NEt_3$. Then, compound **A** (14.2 g, 53.8 mmol) and trimethylsilylacetylene (8.5 mL, 60.1 mmol) were added under argon. The mixture was stirred over night at 80 °C. The reaction was monitored by GC-MS and, when complete, the solvent was evaporated *in vacuo*. The residue was extracted with *n*-hexane (450 mL), the extract was passed through a silica plug and the solvent was removed from the filtrate by rotary evaporation. The residue was further purified by Kugelrohr distillation (0.1 mbar, 90 °C) to give the product **B** as a colorless liquid (9.36 g, 33.3 mmol, 62%)

**NMR** (δ (ppm), $CDCl_3$): **$^1$H** (300 MHz) 7.18 (m, 2H), 2.37 (m, 6H), 0.24 (s, 9H); **$^{13}$C{$^1$H}** (75 MHz) 138.5, 131.5, 128.3, 121.6, 104.5, 94.6, 23.8, 0.08; **Elemental analysis** calc. (%) for $C_{13}H_{17}SiBr$ C 55.51, H 6.09; found C 55.82, H 6.19.

**Synthesis of Compound C**

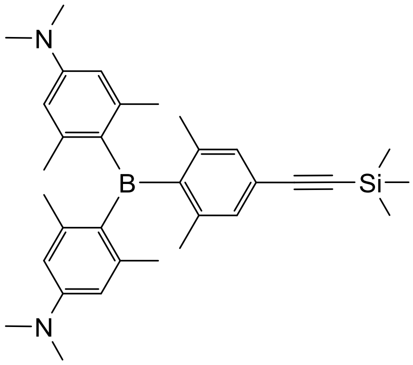


A Schlenk flask was charged with compound **B** (2.05 g, 7.3 mmol) and *n*-hexane (100 mL). The solution was cooled to -78 °C (dry ice/acetone bath). Then 9 mL of a *t*-BuLi solution (1.7 M in pentane, 15.3 mmol, 2.1 eq) was added by syringe. After 2 h, the cooling bath was removed and reaction mixture was stirred for an additional 18 h. Then, an *n*-hexane (100 mL) solution of bis[4-(*N*,*N*-dimethylamino)-2,6-dimethylphenyl]fluoroborane (2.14 g, 6.57 mmol, 0.9 eq) was added at -78 °C. The resulting dark brown solution was allowed to warm to r.t. After stirring overnight at r.t., the reaction mixture was placed in an ultasonic bath for 6 h. Then, $H_2O$ (20 mL) was added, the aqueous layer was extracted with $Et_2O$ (300 mL), and the combined extracts were dried ($Na_2SO_4$). The solvent was removed by rotary evaporation. The dark orange residue was chromatographed (silica, gradient *n*-hexane → 0.5:9.5 v/v $Et_2O$/*n*-hexane). The solvent was removed from the product-containing fractions by rotary evaporation to give **C** as a yellow powder (1.74 g, 3.43 mmol, 47%).

**NMR** (δ (ppm), $CD_2Cl_2$): **$^1$H** (500 MHz) 7.00 (m, 2H) 6.30 (m, 4H), 2.95 (s, 12H), 1.99 (s, 12H), 1.90 (s, 6H), 0.23 (s, 9H); **$^{13}$C{$^1$H}** (125 MHz) 151.8, 151.1, 143.4, 142.9, 140.6, 135.8, 130.7, 122.7, 111.9, 111.8, 106.4, 93.8, 40.1, 24.1, 23.7, 22.7, 0.1; $^{11}$B{$^1$H} (160 MHz) 73; **HRMS** (APCI$^+$): *m*/*z* found: 509.3518 [M+H]; calc. for [$C_{33}H_{45}BN_2Si$+H] 509.3523; **Elemental analysis** calc. (%) for $C_{33}H_{45}BN_2Si$: C 77.93, H 8.92, N 5.51; found: C 78.04, H 9.36, N 5.31.

**Synthesis of Compound 1N**

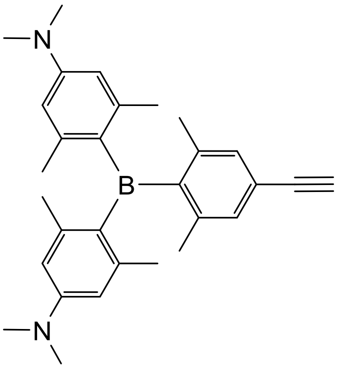

A round bottom flask was charged with **C** (767 mg, 1.51 mmol), KOH (438 mg, 7.54 mmol, 5 eq) and a mixture of MeOH/THF (1:1). The reaction mixture was stirred at r.t. After 12 h, the solvent was removed by rotary evaporation. Water (100 mL) was added and the mixture was extracted with $CH_2Cl_2$ (300 mL). The combined extracts were dried ($Na_2SO_4$), the solvent was removed by rotary evaporation, and the residue was washed with MeOH (30 mL) and dried in vacuo to give **1N** as a bright yellow powder (642 mg, 1.47 mmol, 97%).

**NMR** (δ (ppm), $CD_2Cl_2$): $^{1}$**H** (500 MHz) 7.03 (s, 2H), 6.30 (m, 4H), 3.08 (s, 1H) 2.95 (s, 12H), 2.00 (overlapped bs, 12H), 1.90 (bs, 6H); $^{13}$**C{$^{1}$H}** (125 MHz) 151.8, 151.3, 143.4, 142.9, 140.6, 135.7, 130.9, 121.6, 111.9, 111.8, 84.8, 76.8, 40.1, 24.1, 23.8, 22.7; $^{11}$**B{$^{1}$H}** (160 MHz) 73; **HRMS** ($ASAP^+$): *m*/*z* found: 437.3120 [M+H]; calc. for [$C_{30}H_{37}BN_2$+H] 437.3128; **Elemental analysis** calc. (%) for $C_{30}H_{37}BN_2$: C 82.56, H 8.55, N 6.42; found: C 81.92, H 8.31, N 6.33.

## Synthesis of Compound 2N

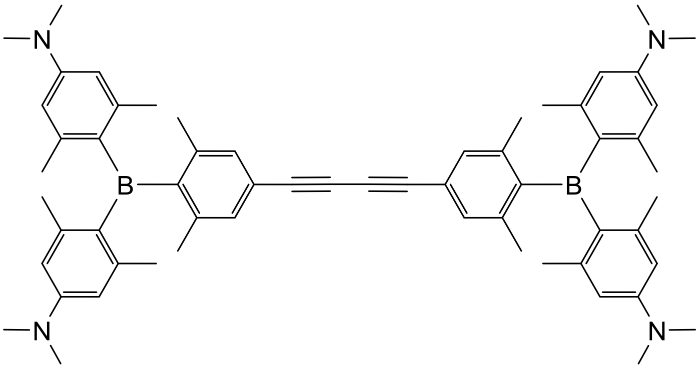


A round bottom flask was charged with **1N** (600 mg, 1.37 mmol), CuI (13.1 mg, 0.069 mmol), $PdCl_2(PPh_3)_2$ (19.3 mg, 0.027 mmol), $I_2$ (174.5 mg, 0.687 mmol) and a mixture of $NEt_3$/THF (1:3). The reaction mixture was stirred at r.t. for 1 day. Then the solvent was removed by rotary evaporation. The residue was washed with MeOH (30 mL), dissolved in $CH_2Cl_2$ and filtered through a plug of silica. Evaporation of the solvent from the filtrate gave the product as an orange powder (453 mg, 0.521 mmol, 76%).

**NMR** (δ (ppm), $CD_2Cl_2$): **$^1$H** (500 MHz) 7.07 (s, 4H) 6.30 (m, 8H), 2.95 (s, 24H), 2.00 (s, 24H), 1.91 (s, 12H); **$^{13}$C{$^1$H}** (125 MHz) 152.1, 151.8, 143.4, 142.9, 140.8, 135.7, 131.2, 121.3, 111.9, 111.8, 82.7, 73.9, 40.1, 24.1, 23.8, 22.8; **$^{11}$B{$^1$H}** (160 MHz) 74; **HRMS** (APCI$^+$): *m/z* found: 871.6009 [M+H]; calc. for [$C_{60}H_{72}B_2N_4$+H] 871.6021; **Elemental analysis** calc. (%) for $C_{60}H_{72}B_2N_4$: C 82.75, H 8.33, N 6.43; found: C 82.31, H 8.46, N 6.56.

**Synthesis of Compound $1^{2+}$**

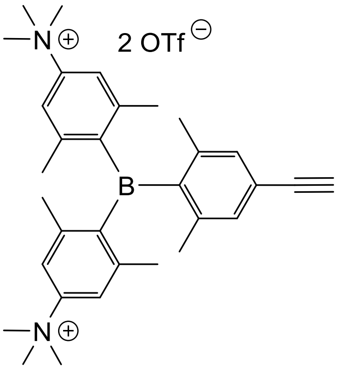

A scintillation vial was charged with **1N** (300 mg, 0.687 mmol) and $CH_2Cl_2$ (5 mL) in a glovebox and 158 μL of MeOTf (1.40 mmol, 2.1 eq) was added. The reaction mixture was stirred at r.t. for 1 day. The solution was concentrated to ca. 1 mL, and the product was precipitated by addition of $Et_2O$. The resulting white solid was collected and washed with $Et_2O$ and $CH_2Cl_2$ and dried *in vacuo*. (338 mg, 0.442 mmol, 64%).

**NMR** (δ (ppm), $CD_3OD$): **$^1$H** (500 MHz) 7.57 (bs, 4H), 7.13 (s, 2H), 3.65 (s, 24H), 3.57 (s, 1H), 2.18 (s, 12H), 2.14 (s, 12H), 2.01 (s, 12H); **$^{13}$C{$^1$H}** (125 MHz) 149.8, 149.0, 146.8, 144.9, 144.6, 142.1, 132.6, 126.4, 121.8 (q, $^1J_{CF}$ = 319 Hz, $OTf^-$), 120.2, 84.1, 80.1, 57.5, 23.5, 23.1; **$^{11}$B{$^1$H}** (160 MHz) 79; **$^{11}$B SS-NMR** (128 MHz) 77; **HRMS** ($ESI^+$): *m/z* found: 615.3025 $[M\text{-}OTf]^+$; calc for $[M\text{-}OTf]^+$ 615.3034; 233.1753 $[M\text{-}2OTf]^{2+}$; calc. for $[M\text{-}2OTf]^{2+}$ 233.1754; **Elemental analysis** calc. (%) for $C_{34}H_{43}BF_6N_2O_6S_2$: C 53.41, H 5.67, N 3.66, S 8.39; found: C 53.42, H 5.78, N 3.81, S 7.96.

**Synthesis of Compound $2^{4+}$**

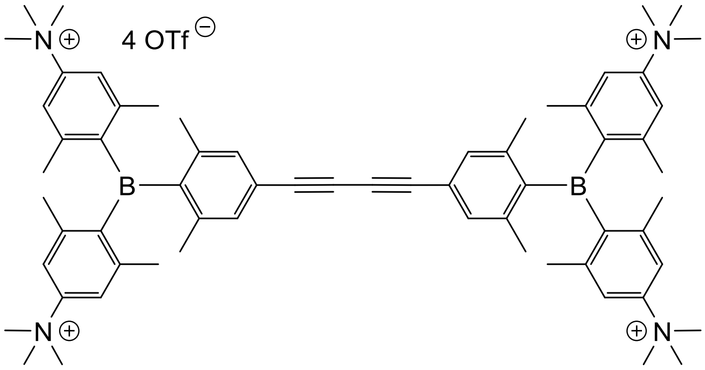


A scintillation vial was charged with **2N** (252 mg, 0.287 mmol) and $CH_2Cl_2$ (5 mL) in a glovebox, and 142 µl of MeOTf (1.292 mmol, 4.5 eq) was added. The reaction mixture was stirred at r.t. for 1 day. Then solution was concentrated to ca. 1 mL. The product was precipitated by addition of $Et_2O$. The pale yellow product was collected and washed with cold acetone and $Et_2O$ and dried *in vacuo*. (101 mg, 0.066 mmol, 23%).

**NMR** (δ (ppm), $CD_3OD$): **$^1$H** (500 MHz) 7.58 (m, 8H), 7.21 (s, 4H), 3.66 (s, 36H), 2.19 (s, 12H), 2.15 (s, 12H), 2.03 (s, 12H); **$^{13}$C{$^1$H}** (125 MHz) 149.9, 148.9, 147.9, 144.9, 144.7, 142.4, 132.9, 125.3, 121.8 (q, $^1J_{CF}$ = 319 Hz, $OTf^-$), 120.2, 82.8, 75.6, 57.5, 23.5, 23.1; **$^{11}$B SS-NMR** (128 MHz) 77; **HRMS** ($ESI^+$): *m*/*z* found: 232.6716 $[M\text{-}4OTf]^{4+}$; calc for $[M\text{-}4OTf]^{4+}$ 232.6715; 359.8792 $[M\text{-}3OTf]^{3+}$; calc. for $[M\text{-}3OTf]^{3+}$ 359.8795; 614.2950 $[M\text{-}2OTf]^{2+}$; calc. for $[M\text{-}2OTf]^{2+}$ 614.2956; **Elemental analysis** calc. (%) for $C_{68}H_{84}B_2F_{12}N_4O_{12}S_4$: C 53.48, H 5.54, N 3.67, S 8.40; found: C 51.46, H 5.38, N 3.69, S 8.05.

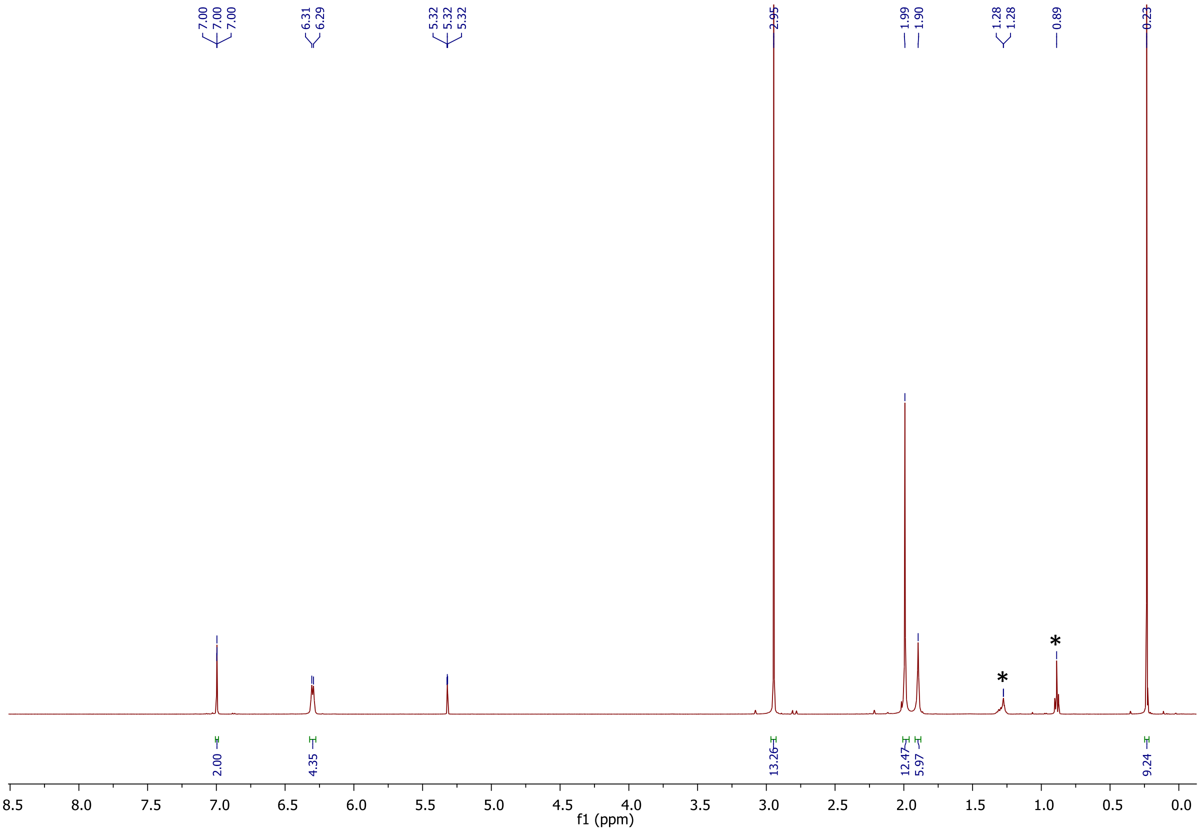


**Figure S1.** $^{1}H$ NMR spectrum of **C** in $CD_2Cl_2$ at 500 MHz. The solvent (*n*-hexane) peaks are labelled by *.

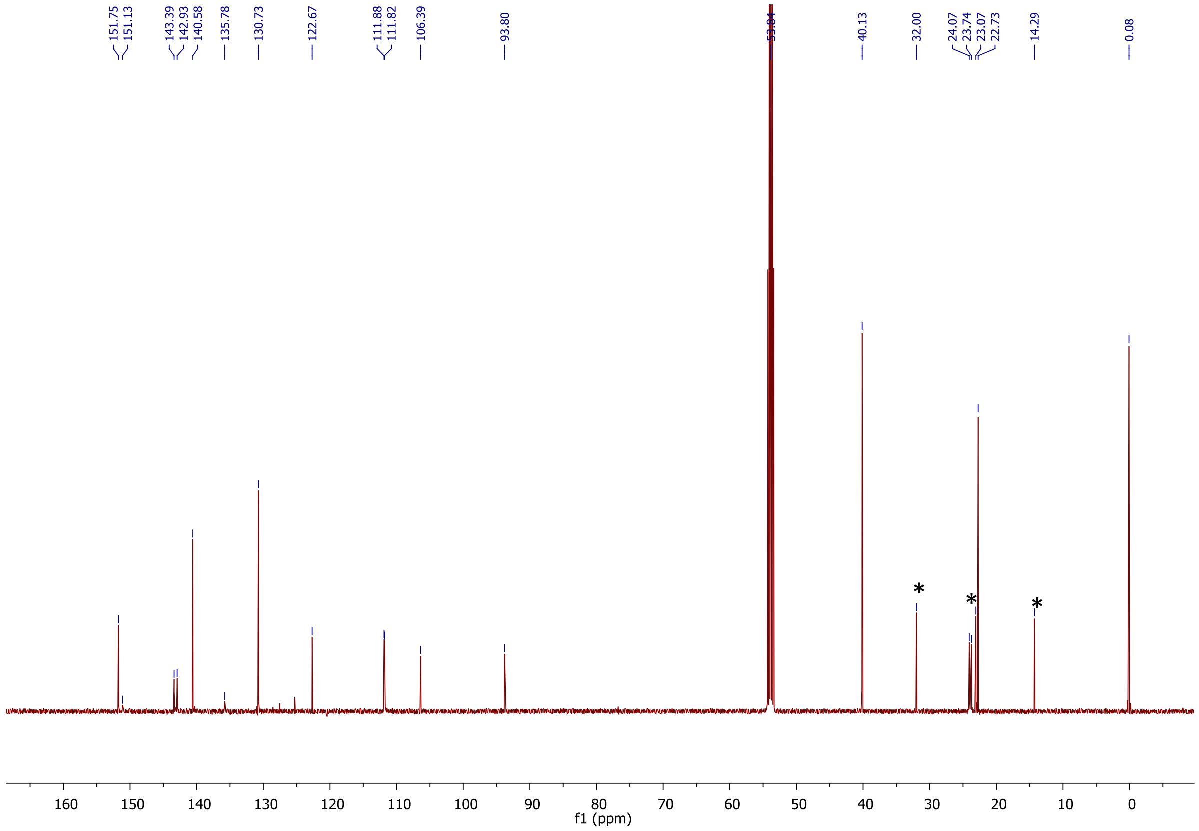


**Figure S2.** $^{13}C\{^{1}H\}$ NMR spectrum of **C** in $CD_2Cl_2$ at 125 MHz. The solvent (*n*-hexane) peaks are labelled by *.

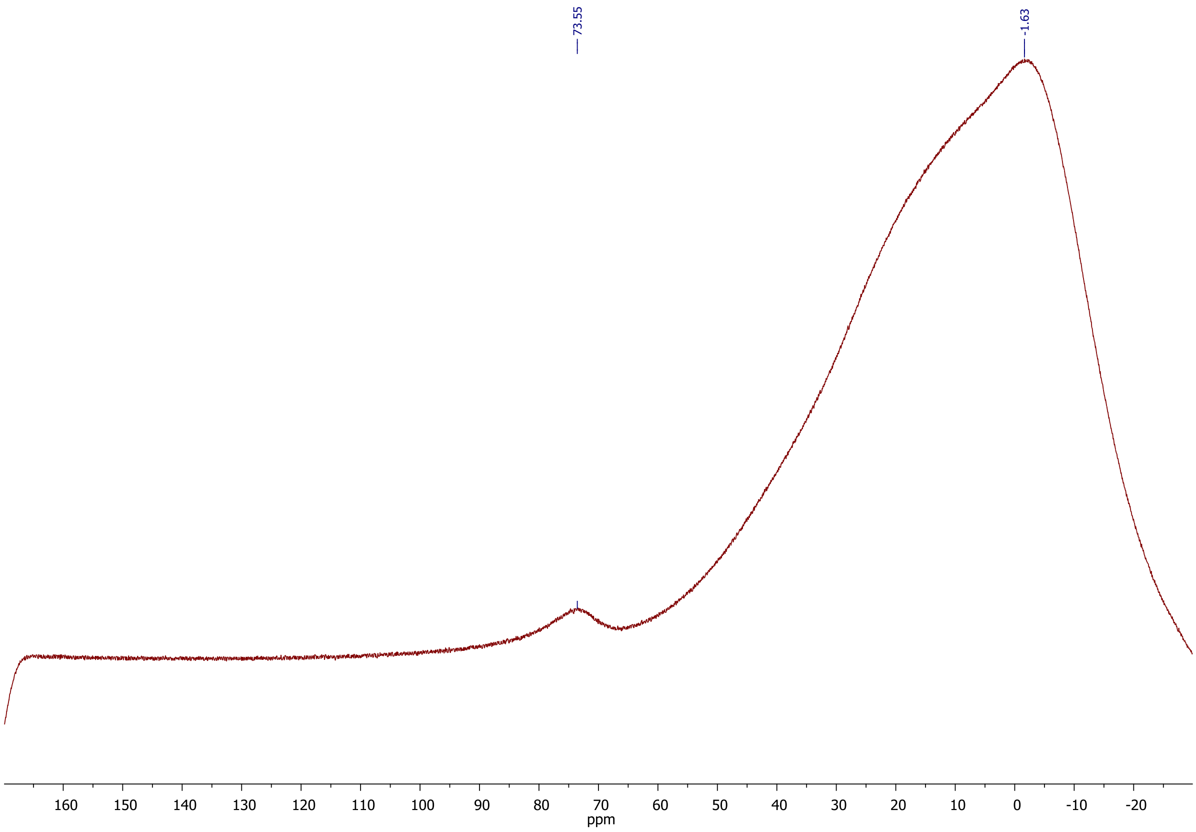


**Figure S3.** $^{11}B\{^{1}H\}$ NMR spectrum of **C** in $CD_2Cl_2$ at 160 MHz.

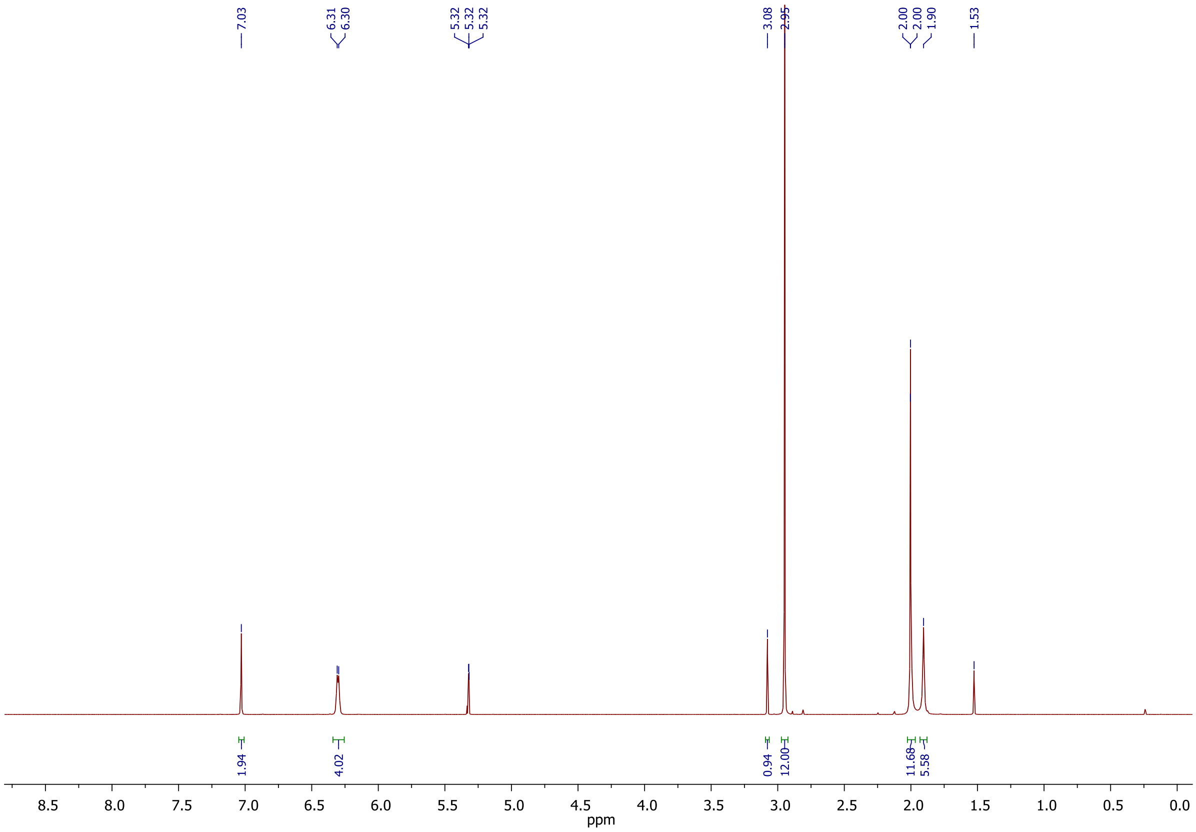


**Figure S4.** $^{1}H$ NMR spectrum of **1N** in $CD_2Cl_2$ at 500 MHz.

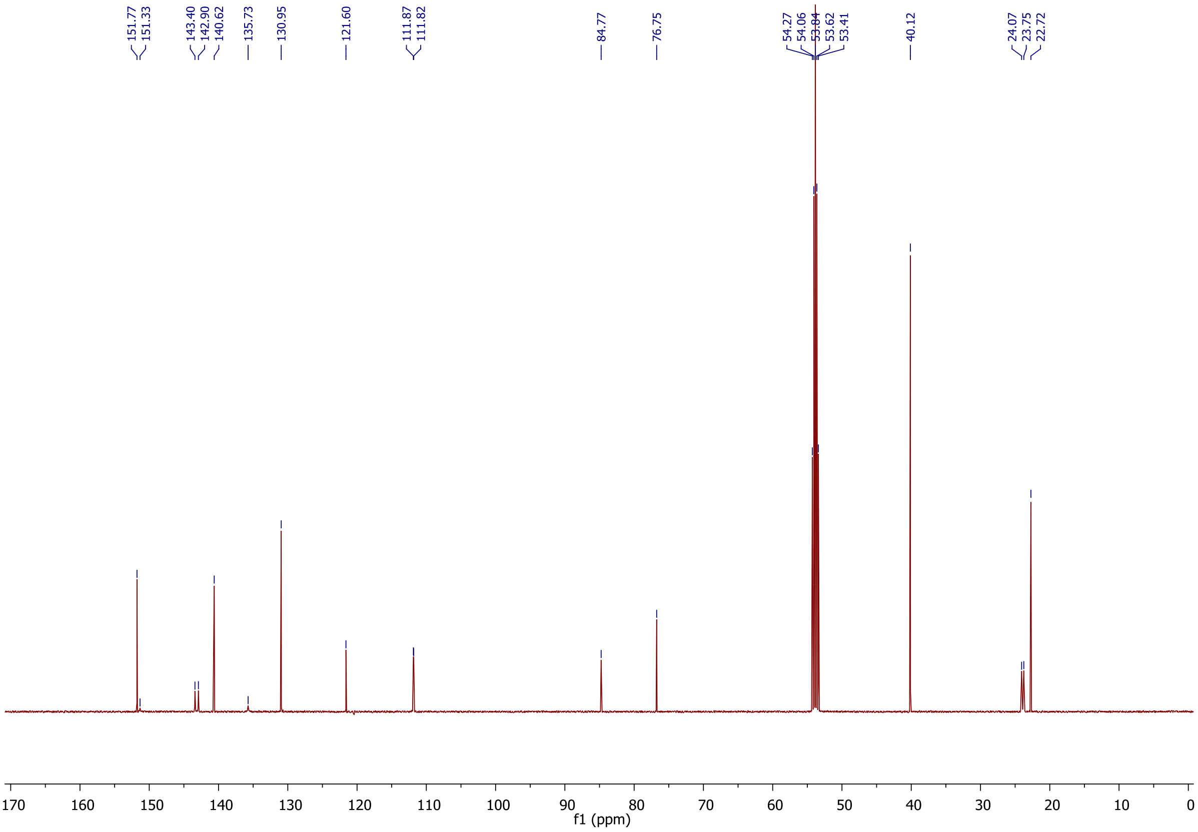


**Figure S5.** $^{13}C\{^1H\}$ NMR spectrum of **1N** in $CD_2Cl_2$ at 125 MHz.

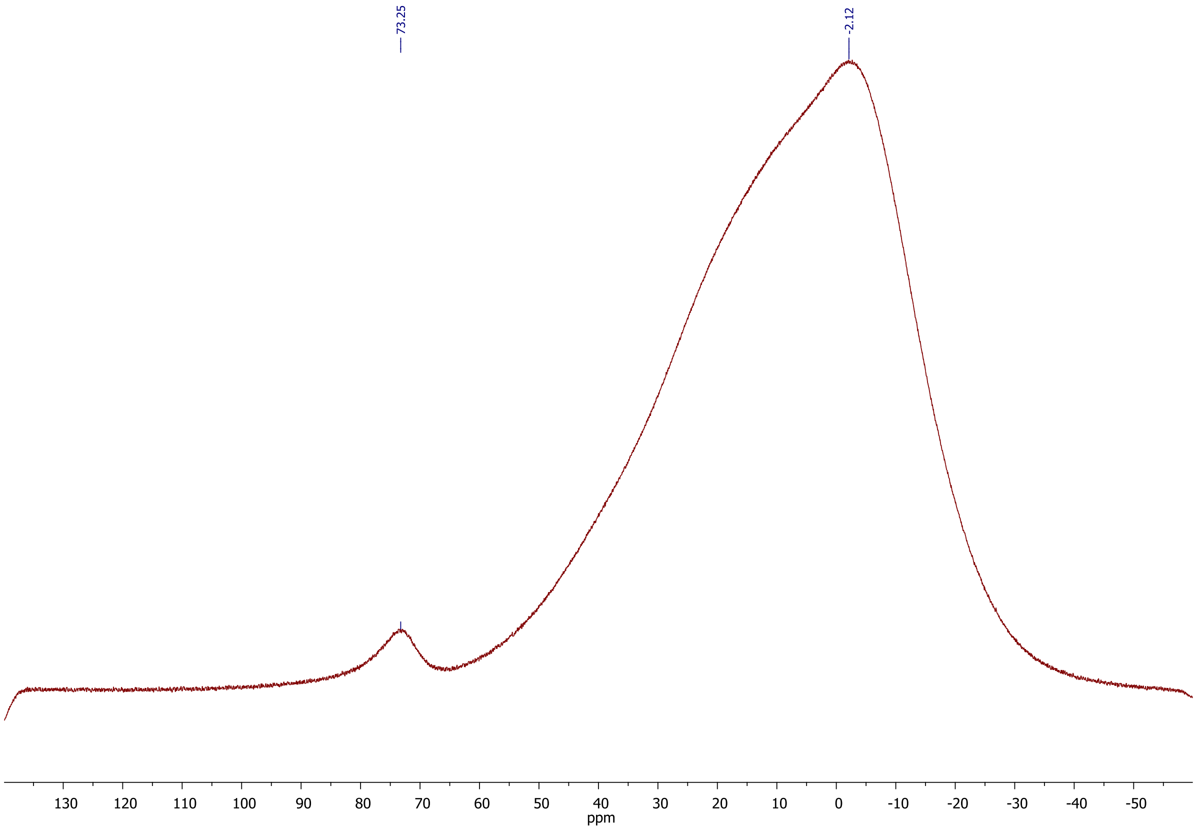


**Figure S6.** $^{11}B\{^1H\}$ NMR spectrum of **1N** in $CD_2Cl_2$ at 160 MHz.

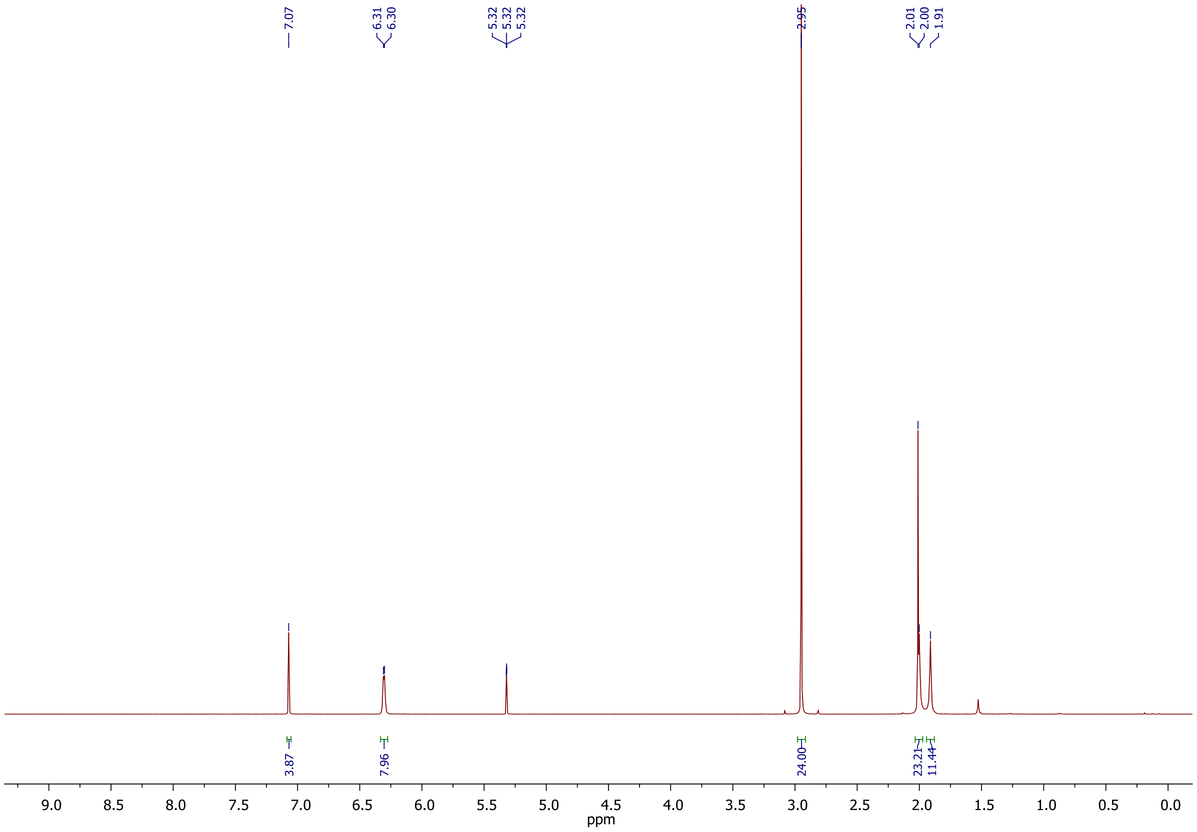


**Figure S7.** $^{1}H$ NMR spectrum of **2N** in $CD_2Cl_2$ at 500 MHz.

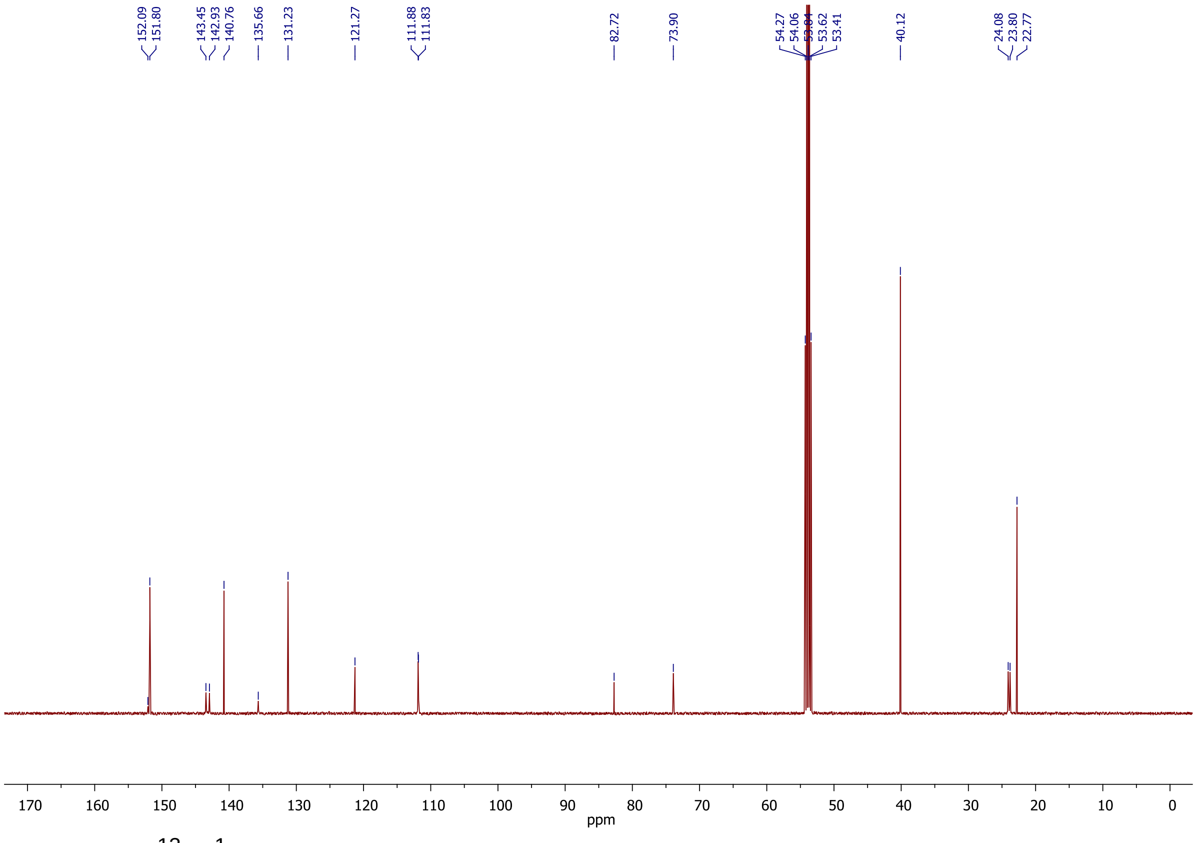


**Figure S8.** $^{13}C\{^{1}H\}$ NMR spectrum of **2N** in $CD_2Cl_2$ at 125 MHz.

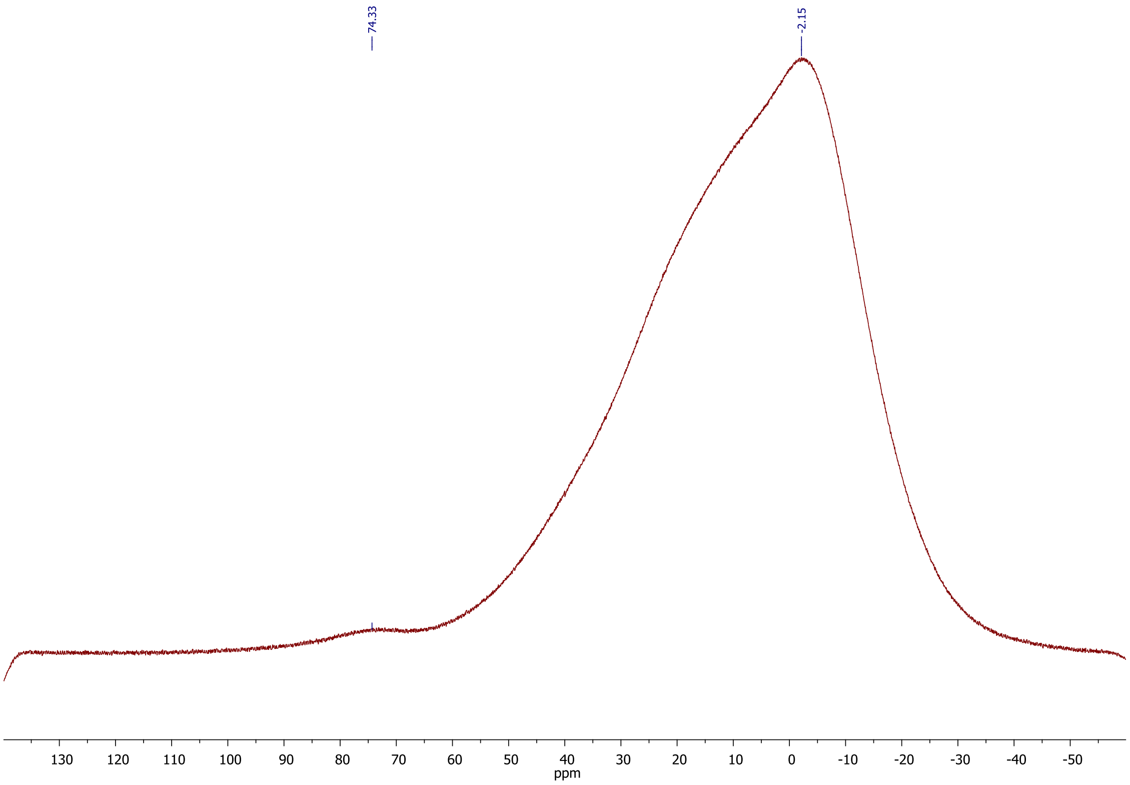


**Figure S9.** $^{11}B\{^{1}H\}$ NMR spectrum of **2N** in $CD_2Cl_2$ at 160 MHz

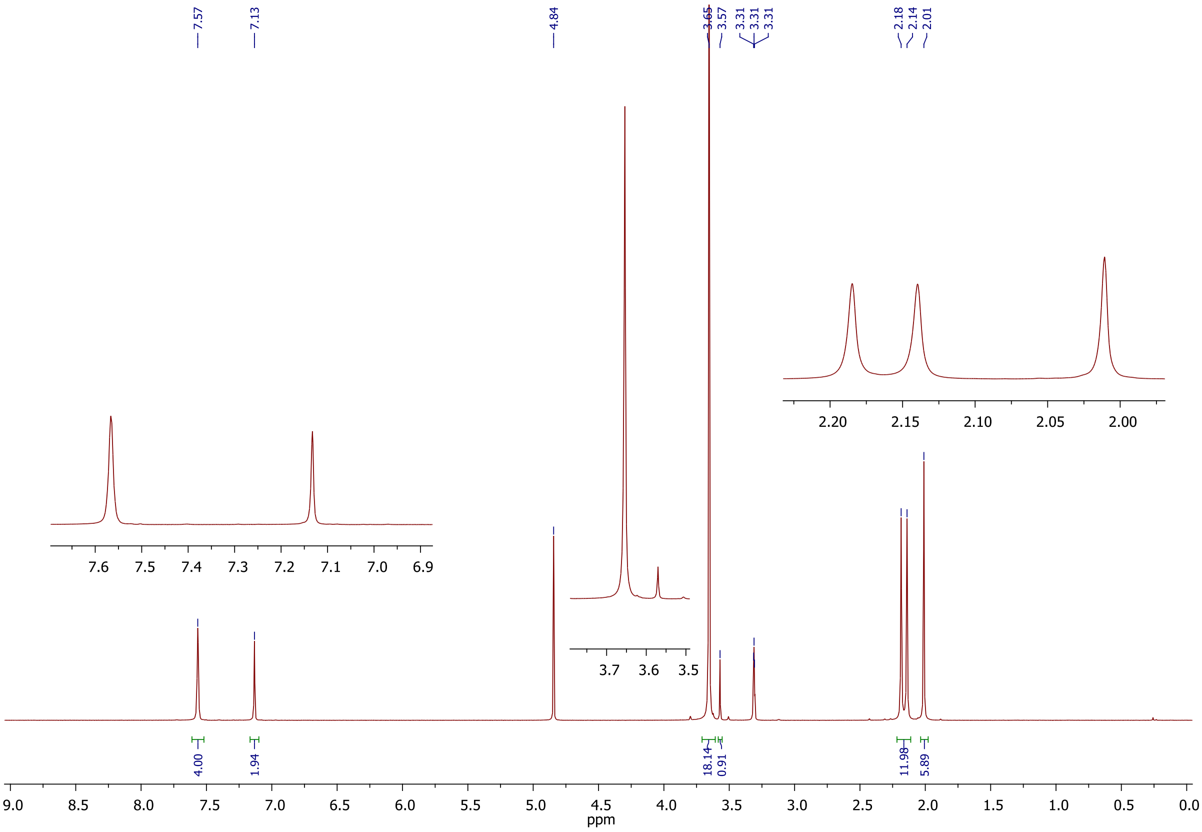


**Figure S10.** $^{1}H$ NMR spectrum of $\mathbf{1}^{2+}$ in $CD_3OD$ at 500 MHz

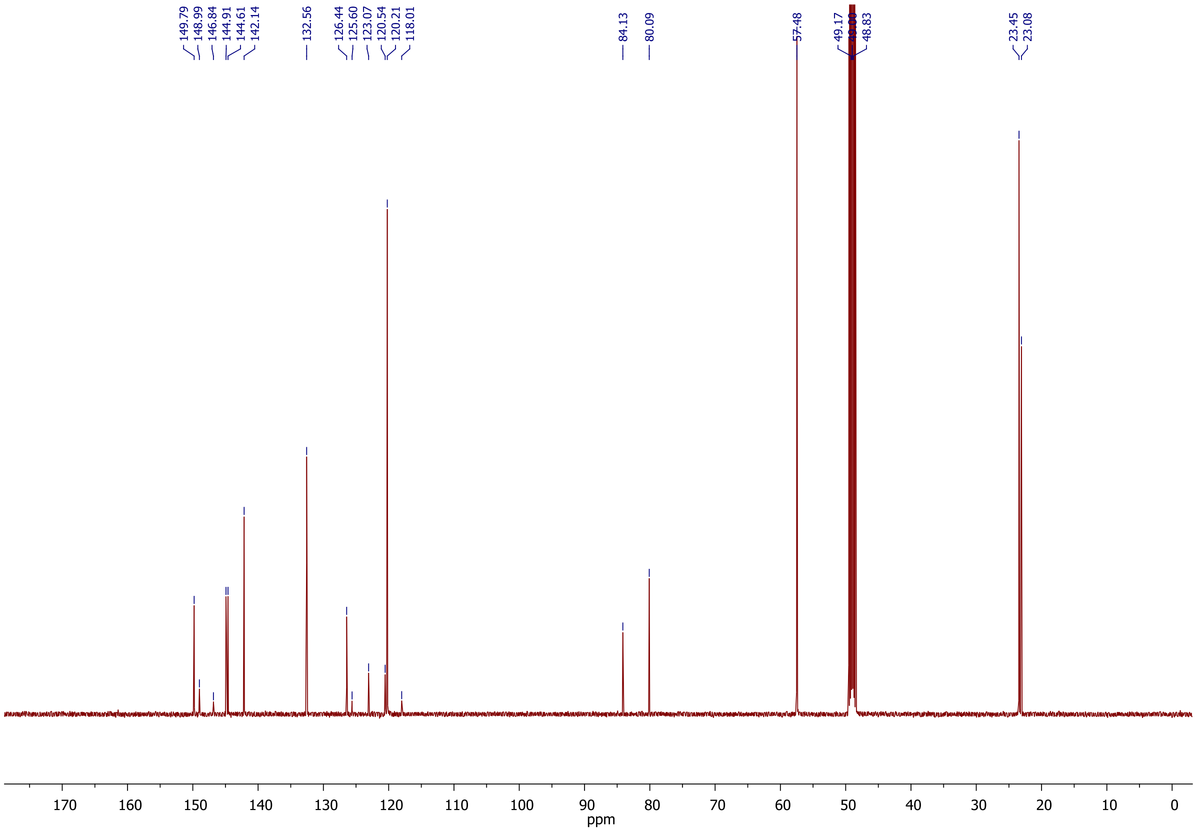


**Figure S11.** $^{13}C\{^{1}H\}$ NMR spectrum of $\mathbf{1}^{2+}$ in $CD_3OD$ at 125 MHz.

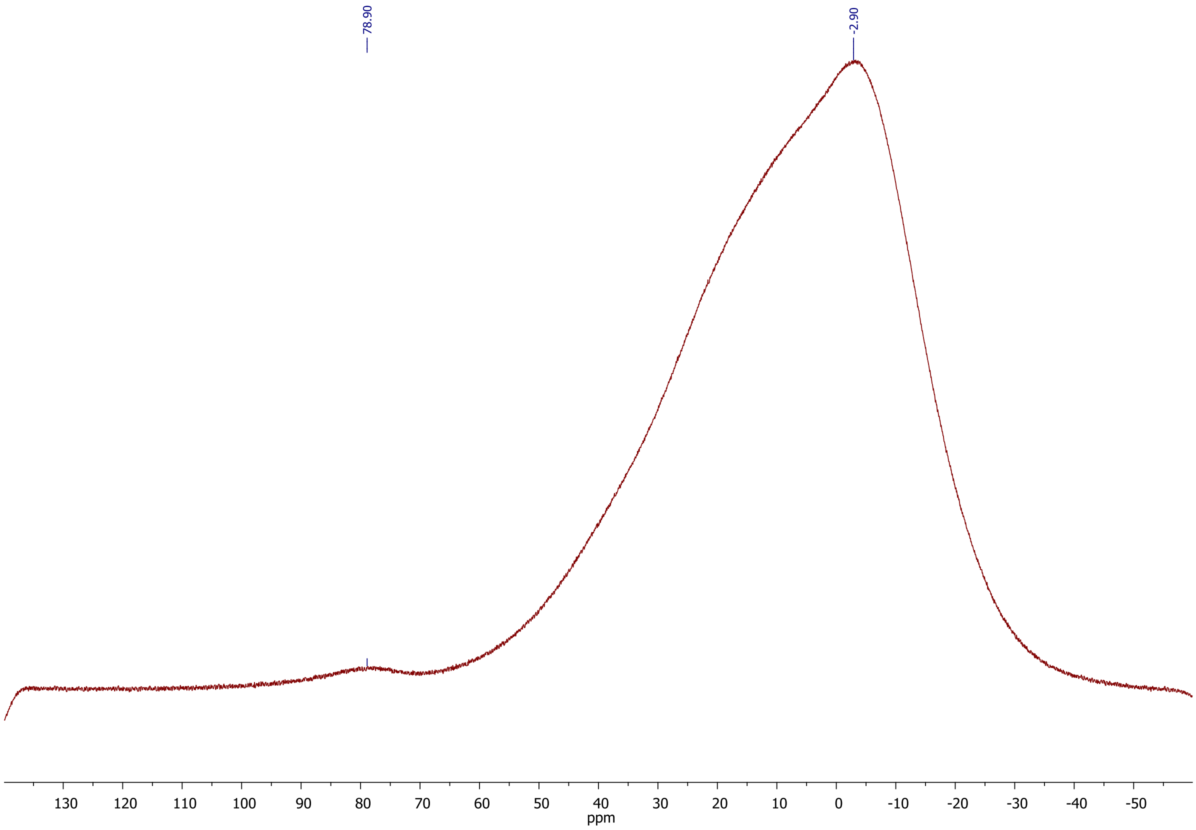


**Figure S12.** $^{11}B\{^{1}H\}$ NMR spectrum of $\mathbf{1}^{2+}$ in $CD_3OD$ at 160 MHz.

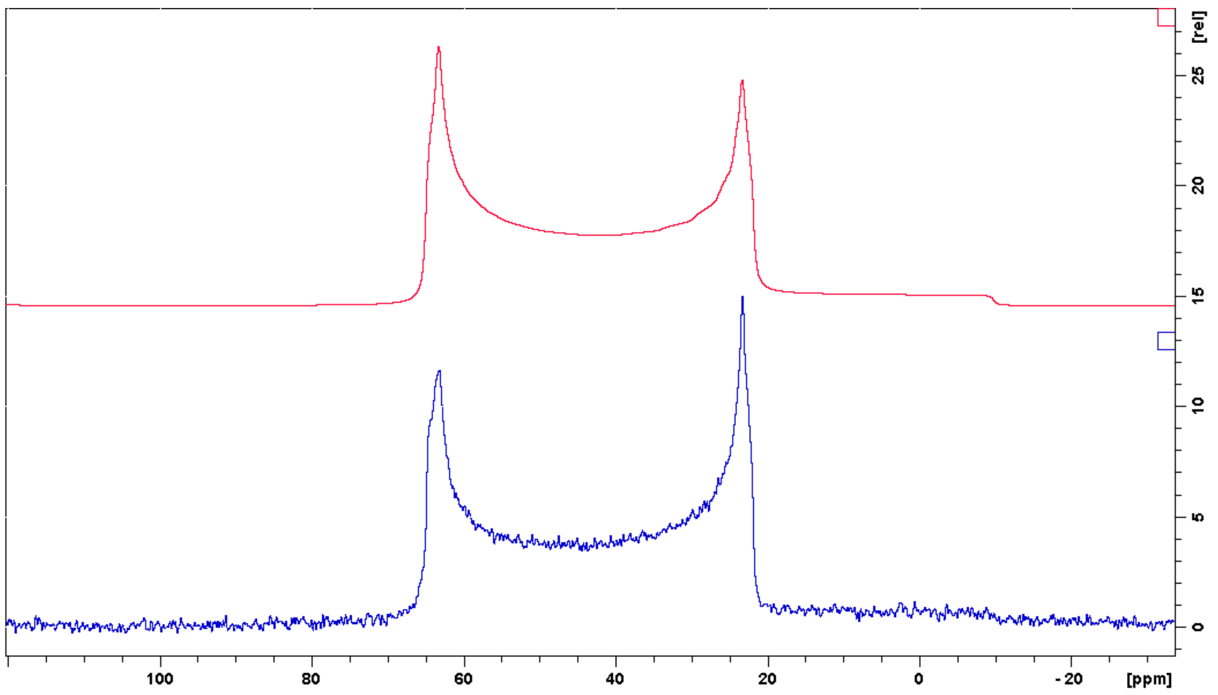


**Figure S13.** Solid-state $^{11}B\{^{1}H\}$ NMR spectrum of $\mathbf{1}^{2+}$ at 128 MHz (bottom). Simulated spectrum (top), isotropic chemical shift $\delta_{iso}$ = 76.6 ppm, quadrupolar coupling constant $C_Q$ = 4.78 MHz, quadrupolar asymmetry parameter $\eta_{Quad}$ = 0.0.

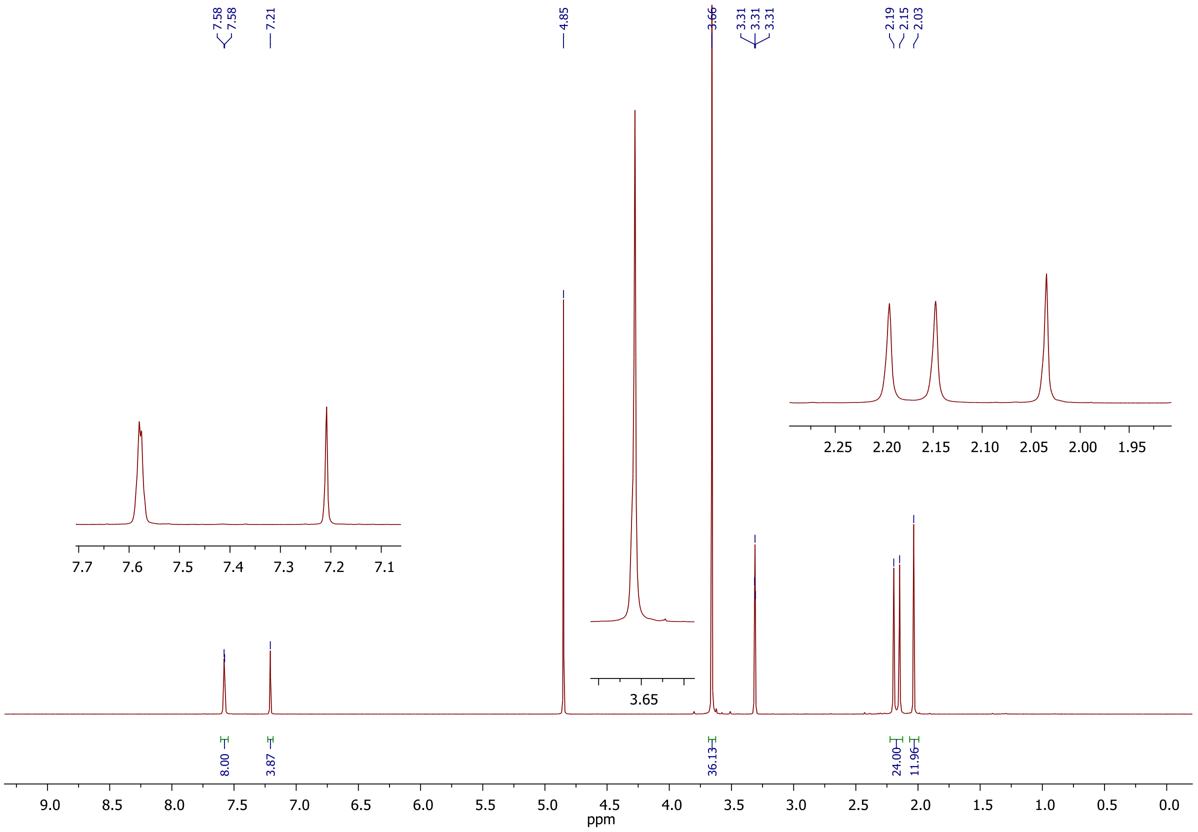


**Figure S14.** $^{1}H$ NMR spectrum of $\mathbf{2}^{4+}$ in $CD_3OD$ at 500 MHz.

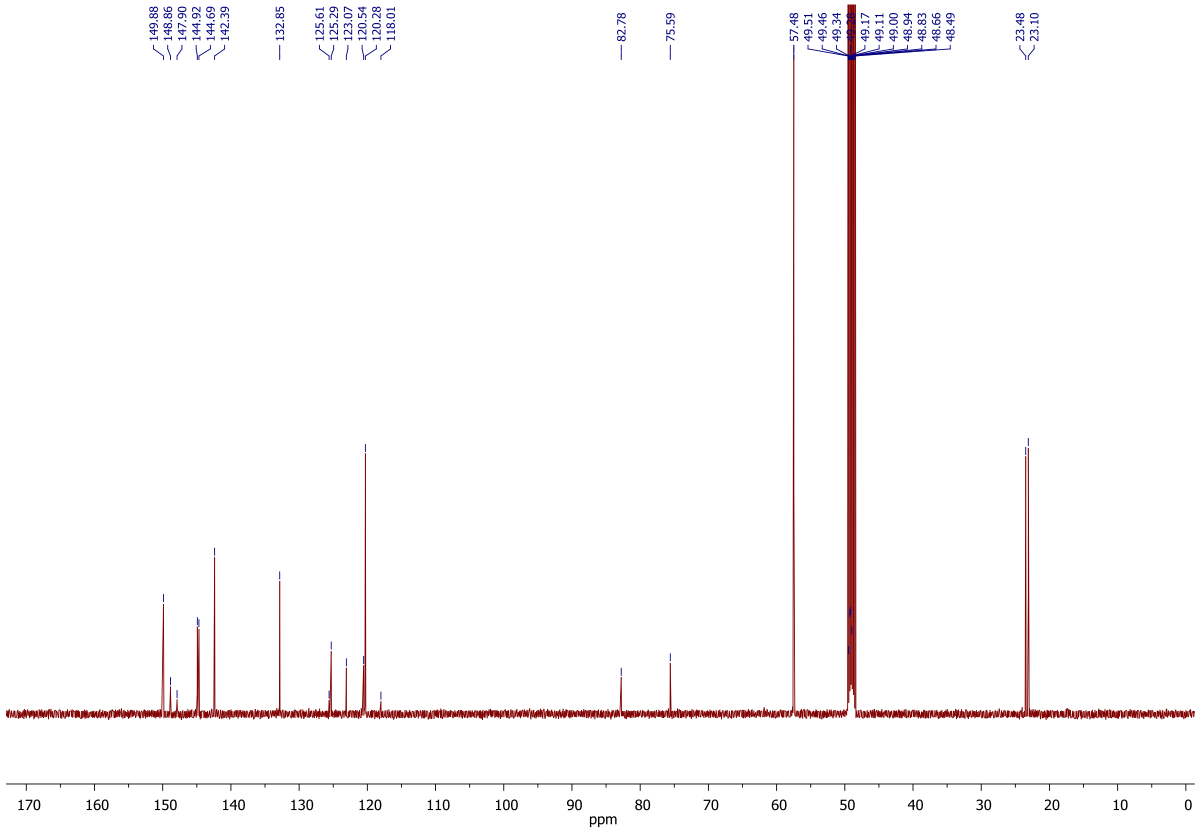


**Figure S15.** $^{13}C\{^{1}H\}$ NMR spectrum of **$2^{4+}$** in $CD_3OD$ at 125 MHz.

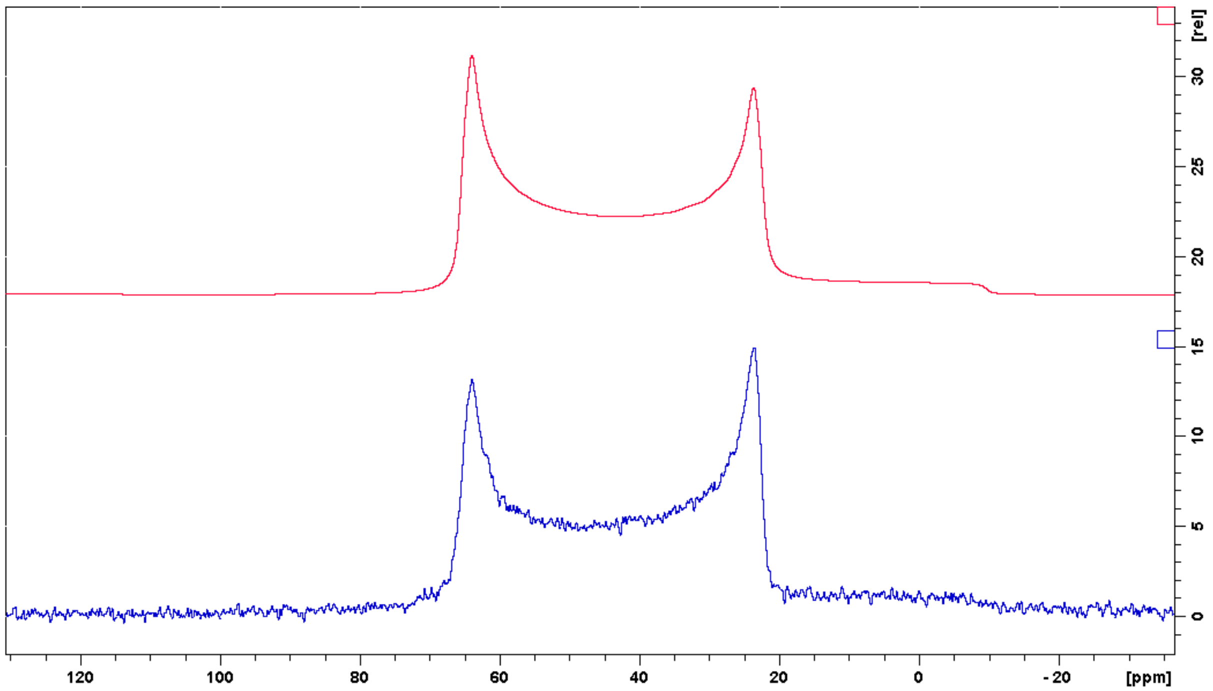


**Figure S16.** Solid-state $^{11}B\{^{1}H\}$ NMR spectrum of **$2^{4+}$** at 128 MHz (bottom). Simulated spectrum (top), isotropic chemical shift $\delta_{iso}$ = 77.4 ppm, quadrupolar coupling constant $C_Q$ = 4.79 MHz, quadrupolar asymmetry parameter $\eta_{Quad}$ = 0.0.

**Single-crystal X-ray diffraction**

Slow evaporation of solvent from a $CH_2Cl_2$ solution of **1N** resulted in yellow single crystal plates. Slow evaporation of solvent from a solution of **2N** in a mixture of acetone/$CH_2Cl_2$ (1:1) afforded yellow single crystal blocks. In each case, a single crystal suitable for single-crystal X-ray diffraction was selected, coated in perfluoropolyether oil, and mounted on a MiTeGen sample holder. Diffraction data were collected on a BRUKER X8 APEX II 4-circle diffractometer with a CCD area detector using Mo-$K_\alpha$ radiation monochromated by multi-layer focusing mirrors. The crystals were cooled using an Oxford Cryostreams low-temperature device. Data were collected at 100 K. The images were processed and corrected for Lorentz-polarization effects and absorption as implemented in the Bruker software packages. The structures were solved using the intrinsic phasing method (SHELXT)[5] and Fourier expansion technique. All non-hydrogen atoms were refined in anisotropic approximation, with hydrogen atoms 'riding' in idealized positions, by full-matrix least squares against $F^2$ of all data, using SHELXL[6] software. Diamond[7] software was used for graphical representation.

**Table S1.** Crystallographic data for **1N**, and **2N**·2($C_3H_6O$).

| **Data** | **1N** | **2N**·2($C_3H_6O$) |
|---|---|---|
| Empirical formula | $C_{30}H_{37}BN_2$ | $C_{60}H_{72}B_2N_4$·2($C_3H_6O$) |
| Formula weight (g·mol$^{-1}$) | 436.42 | 986.99 |
| Temperature (K) | 100(2) | 100(2) |
| Radiation, λ (Å) | Mo-$K_\alpha$ 0.71073 | Mo-$K_\alpha$ 0.71073 |
| Crystal size (mm³) | 0.33×0.40×0.47 | 0.01×0.35×0.44 |
| Crystal color, habit | yellow block | yellow plate |
| Crystal system | Monoclinic | Monoclinic |
| Space group | *C*2/*c* | *P*2$_1$/*n* |
| *a* (Å) | 11.005(3) | 8.652(5) |
| *b* (Å) | 12.648(3) | 15.973(9) |
| *c* (Å) | 18.608(3) | 21.537(11) |
| $\alpha$ (°) | 90 | 90 |
| $\beta$ (°) | 104.318(8) | 92.70(2) |
| $\gamma$ (°) | 90 | 90 |
| Volume (Å$^3$) | 2509.5(10) | 2973(3) |
| *Z* | 4 | 2 |
| $\rho_{calc}$ (g·cm$^{-3}$) | 1.155 | 1.102 |
| $\mu$ (mm$^{-1}$) | 0.066 | 0.065 |
| *F*(000) | 944 | 1068 |
| $\theta$ range (°) | 2.259 - 26.021 | 1.588 - 26.759 |
| Reflections collected | 21250 | 33532 |
| Unique reflections | 2484 | 6289 |
| Minimum/maximum transmission | 0.6915/0.7458 | 0.6957/0.7454 |
| Parameters / restraints | 157 / 0 | 346 / 0 |
| GooF on $F^2$ | 1.082 | 1.068 |
| $R_1$ [I > 2σ (I)] | 0.0472 | 0.0512 |
| $wR^2$ (all data) | 0.1312 | 0.1282 |
| Max. / min. residual electron density (e·Å$^{-3}$) | 0.350 / –0.206 | 0.294 / –0.252 |

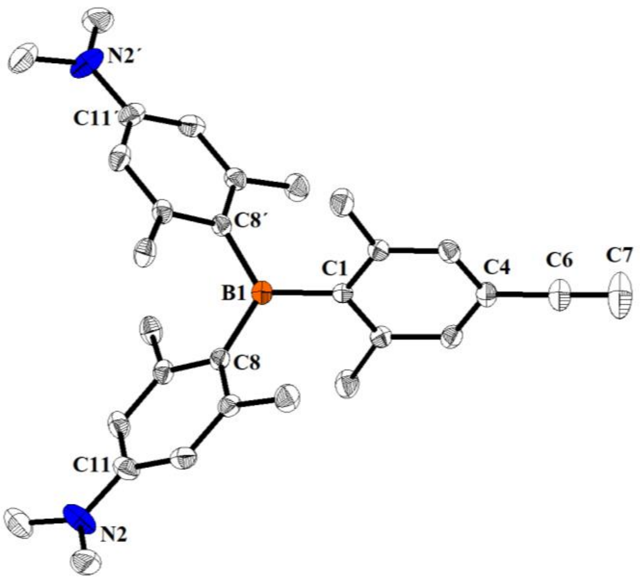


**Figure S17**. The solid state molecular structure of **1N** (50% probability ellipsoids). Hydrogen atoms are omitted for clarity. Angles between the plane of the $BC_3$-core (defined by B1, C1, C8 and C8′) and the planes of the adjacent aryl rings: 53.49(5)° for C1; 47.47(5)° for C8 and C8′. Selected distances and angles for **1N**: B1–C1 1.584(3) Å; B1–C8 1.5707(19) Å; C4–C6 1.439(3) Å; C6–C7 1.185(4) Å; N2–C11 1.379(2) Å; C1–B1–C8 121.721(13)°; C8–B1–C8′ 116.558(9)°. Sum of the C–B–C angles around B: 360.00(12)°.

**Photophysical measurements in solution**

All measurements were performed in standard quartz cuvettes (1 cm × 1 cm cross-section). Solvents used were HPLC grade and were dried using an Innovative Technology Inc. Pure Solvent Purification System. All solutions used in the measurements had a concentration lower than $4\times10^{-5}$ M. All samples were exposed to room light between the measurements which were performed at r.t.

**UV-visible absorption spectra** were measured with an Agilent 8453 diode array UV-visible spectrophotometer or Varian Cary 100 Bio spectrometer. The molar extinction coefficients were calculated from three independently prepared samples. Under the experimental conditions used, the absorbance of **1N,** $\mathbf{1^{2+}}$ and $\mathbf{2^{4+}}$ was proportional to its concentration.

**The excitation and emission spectra** were measured using an Edinburgh Instruments FLSP920 spectrometer equipped with a double monochromator for both excitation and emission, operating in right angle geometry mode, and all spectra were fully corrected for the spectral response of the instrument. For investigations of the influence of temperature increase, excitation and emission spectra were recorded on Varian Cary Eclipse fluorimeter in quartz cuvettes (1 cm) by adding portions of polynucleotide or protein solution into the solution of the studied compound (c = $5\times10^{-7}$ M for **1N** and $\mathbf{1^{2+}}$ and $5\times10^{-8}$ M for $\mathbf{2^{4+}}$).

**The fluorescence quantum yields** were measured using a calibrated integrating sphere (inner diameter: 150 mm) from Edinburgh Instruments combined with the FLSP920 spectrometer described above. For all measurements, the longest-wavelength absorption maximum of the compound in the respective solvent was chosen as the excitation wavelength, unless stated otherwise. **Fluorescence lifetimes** were recorded using the time-correlated single-photon counting (TCSPC) method using an Edinburgh Instruments FLS980 spectrometer equipped with a high speed photomultiplier tube positioned after a single emission monochromator. Measurements were made in right-angle geometry mode, and the emission was collected through a polarizer set to the magic angle. Solutions were excited using Edinburgh Photonics EPLED-280 pulsed diode lasers at 315.8 nm (for compound $\mathbf{1^{2+}}$) and 376.6 nm (for compounds **1N**, **2N** and $\mathbf{2^{4+}}$) at repetition rates of 10 or 20 MHz, as appropriate. The full-width-at-half-maximum (FWHM) of the pulse from the diode laser was *ca.* 80 ps with an instrument response function (IRF) of *ca.* 230 ps

FWHM and *ca.* 200 ps with an instrument response function (IRF) of *ca.* 1120 ps FWHM, respectively. The IRFs were measured from the scatter of an aqueous suspension of Ludox at the excitation wavelength. Decays were recorded to 10 000 counts in the peak channel with a record length of 8 192 channels. The band pass of the emission monochromator and a variable neutral density filter on the excitation side were adjusted to give a signal count rate of < 60 kHz. Iterative reconvolution of the IRF with one decay function and non-linear least-squares analysis were used to analyse the data. The quality of all decay fits was judged to be satisfactory, based on the calculated values of the reduced $\chi^2$ and Durbin-Watson parameters and visual inspection of the weighted residuals.

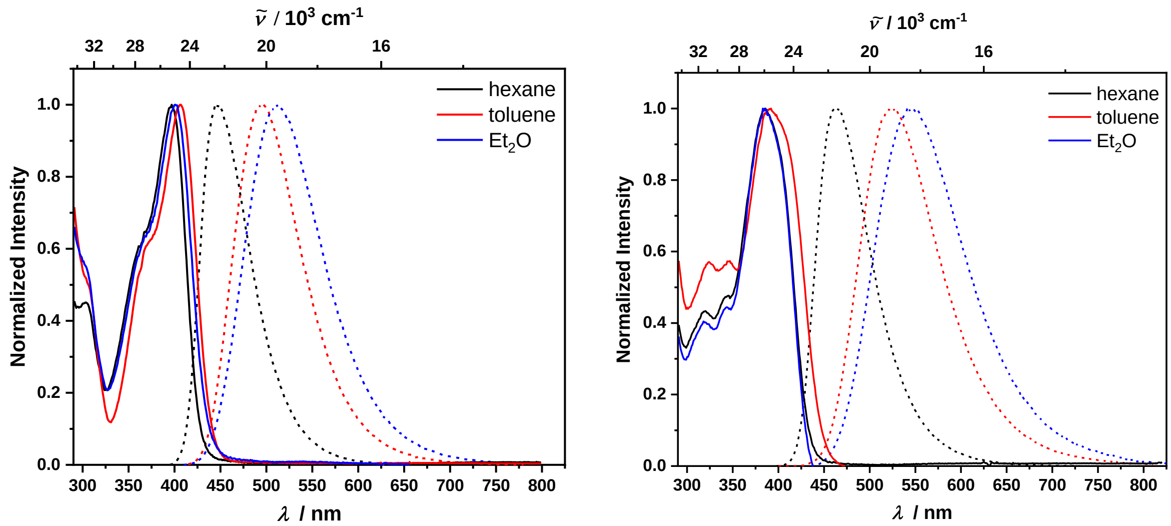


**Figure S18.** Normalized absorption (solid) and emission (dashed) spectra of **1N** (left) and **2N** (right) in *n*-hexane, toluene and $Et_2O$.

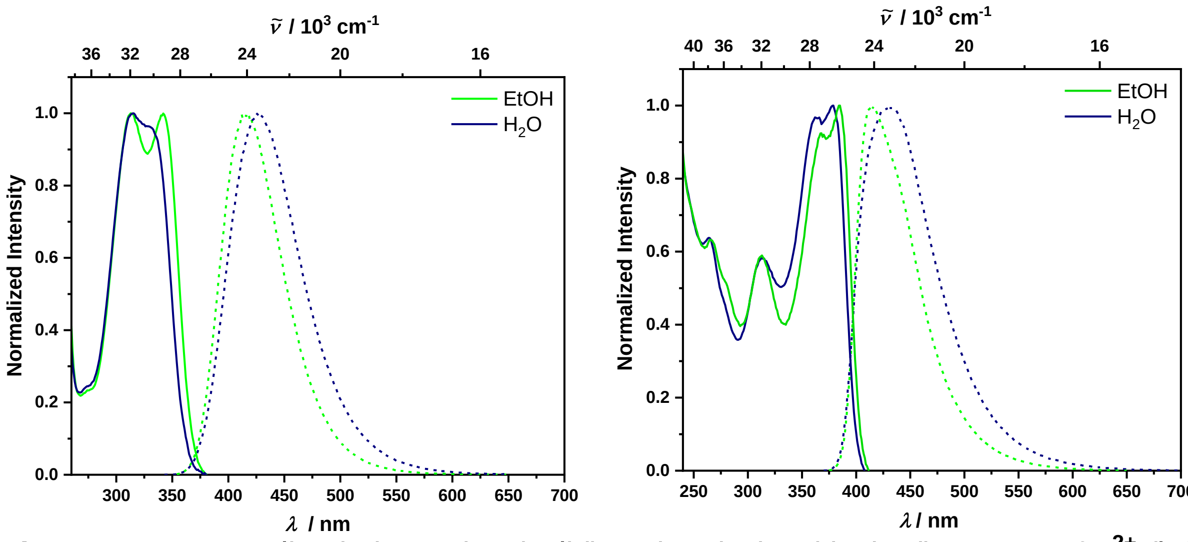


**Figure S19.** Normalized absorption (solid) and emission (dashed) spectra of $\mathbf{1}^{2+}$ (left) and $\mathbf{2}^{4+}$ (right) in EtOH and $H_2O$.

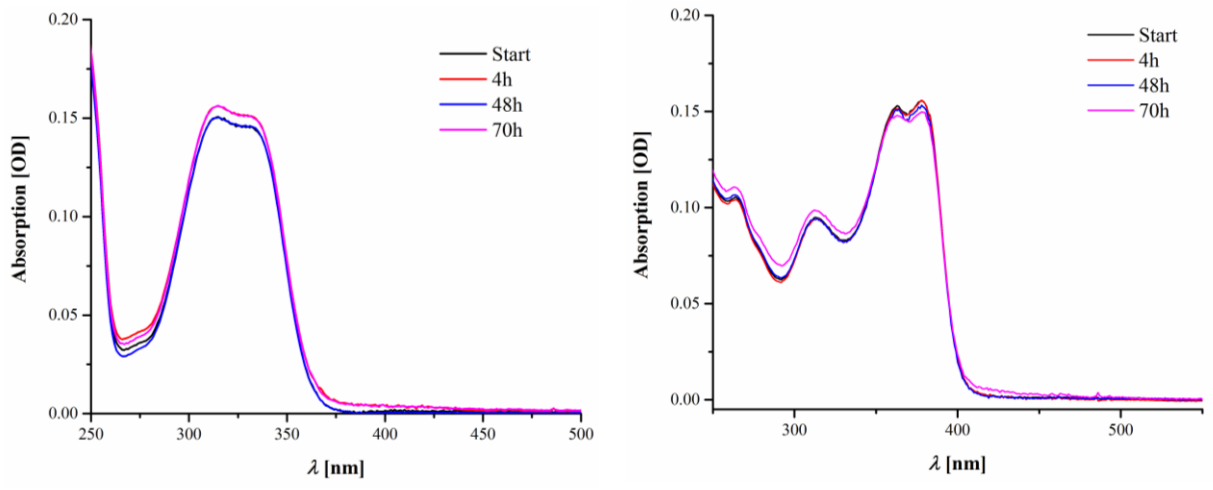


**Figure S20.** Plots of absorption vs. time of **1**$^{2+}$ (left) and **2**$^{4+}$ (right) in $H_2O$.

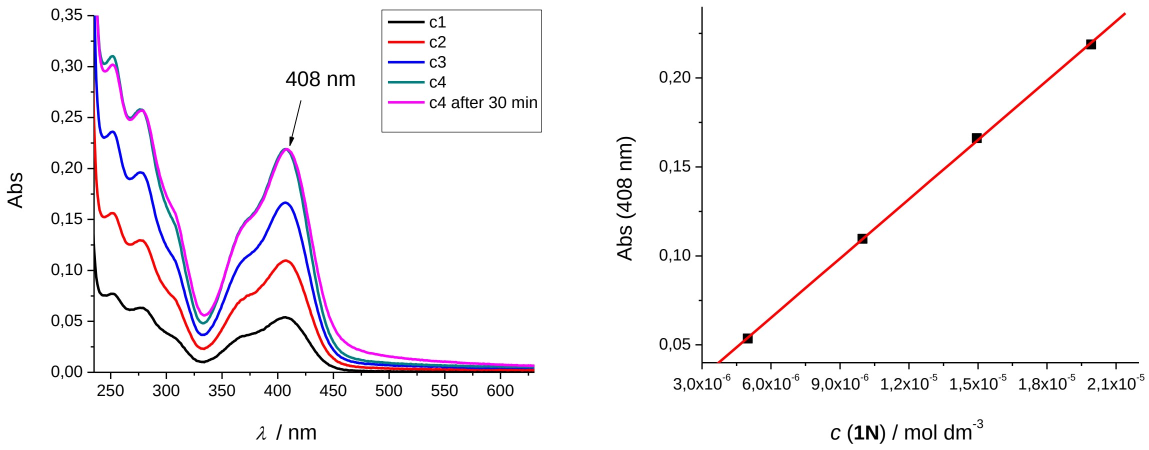


**Figure S21**. UV-Vis spectra of **1N**, c = 5×10$^{-6}$ - 2×10$^{-5}$ M (left); linear dependence (—) of the absorbance at 408 nm (■) on the **1N** concentration (right). Done in Na-cacodylate buffer, pH = 7.0, *I* = 0.05 M.

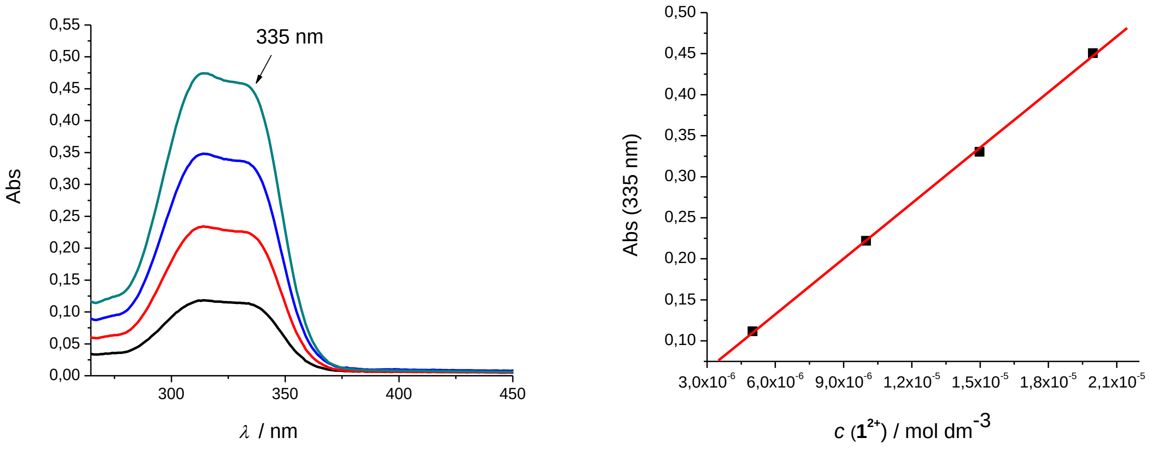


**Figure S22**. UV-Vis spectra of $\mathbf{1}^{2+}$, c = 5×10$^{-6}$ - 2×10$^{-5}$ M (left); linear dependence (—) of the absorbance at 335 nm (■) on the $\mathbf{1}^{2+}$ concentration (right). Done in Na-cacodylate buffer, pH = 7.0, *I* = 0.05 M.

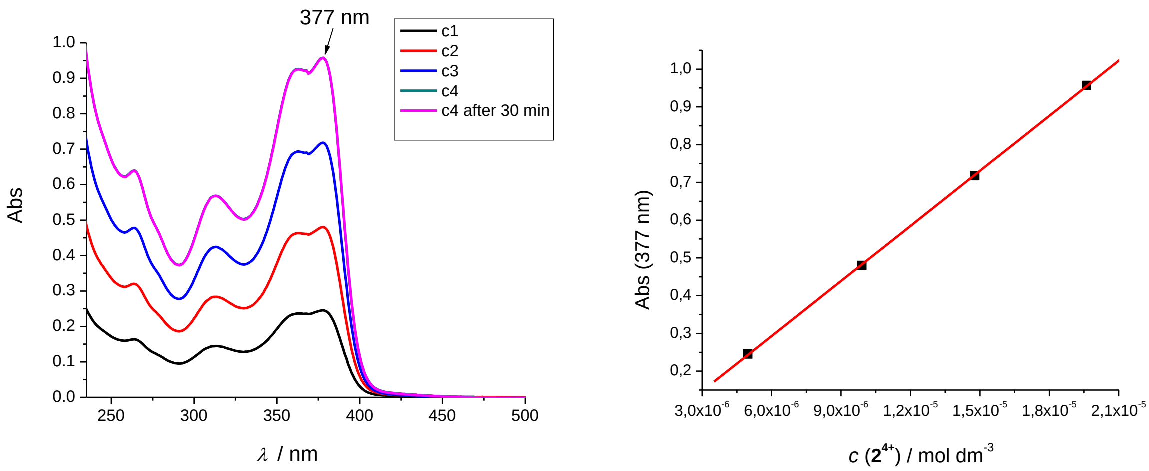


**Figure S23**. UV-Vis spectra of $\mathbf{2}^{4+}$, c = 5×10$^{-6}$ - 2×10$^{-5}$ M (left); linear dependence (—) of the absorbance at 377 nm (■) on the $\mathbf{2}^{4+}$ concentration (right). Done in Na-cacodylate buffer, pH = 7.0, *I* = 0.05 M.

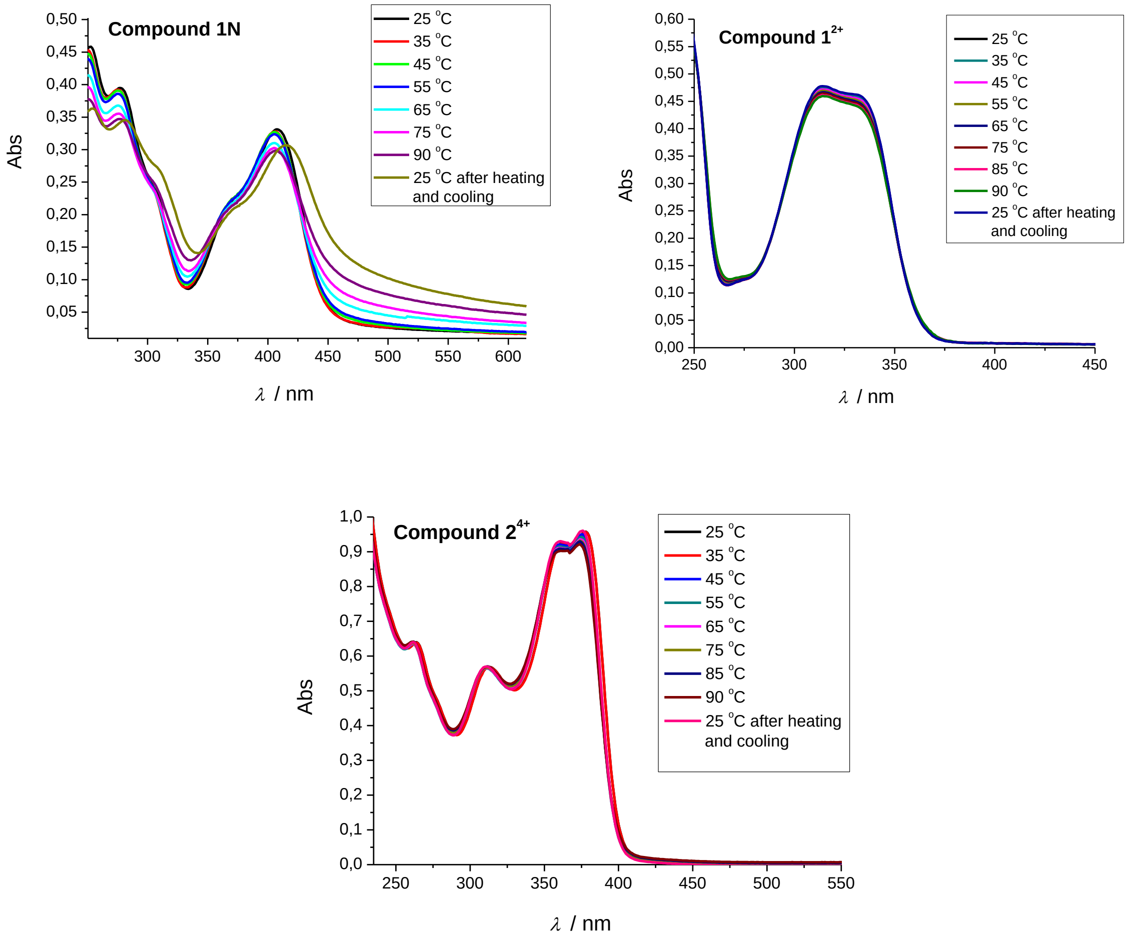


**Figure S24**. Influence of temperature increase (T = 25°C – 90 °C) on UV/Vis spectra of **1N, 1$^{2+}$** and **2$^{4+}$,** $c = 2\times10^{-5}$ M, pH = 7.0. Done in Na-cacodylate buffer, pH = 7.0, $I$ = 0.05 M.

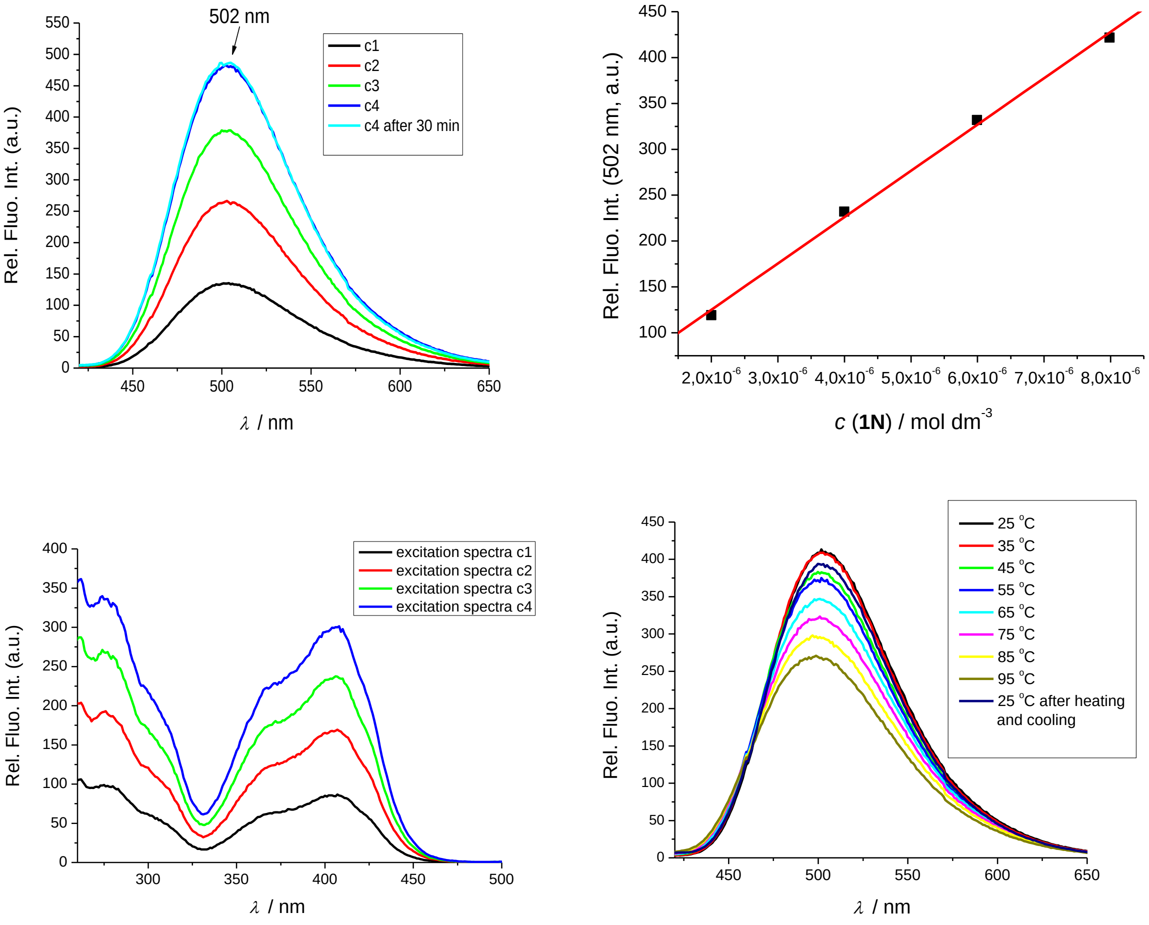


**Figure S25**. Top: Fluorescence spectra of **1N** ($\lambda_{ex}$ = 408 nm) at micromolar concentrations (left); linear dependence (—) of the fluorescence at 502 nm (■) on the **1N** concentration (right); bottom: Excitation spectra of **1N** (left); influence of temperature increase (T = 25°C – 90 °C) on fluorescence spectra of **1N** (c = $8\times10^{-6}$ M) (right). Done in buffer sodium cacodylate (pH = 7.0, *I* = 0.05 M).

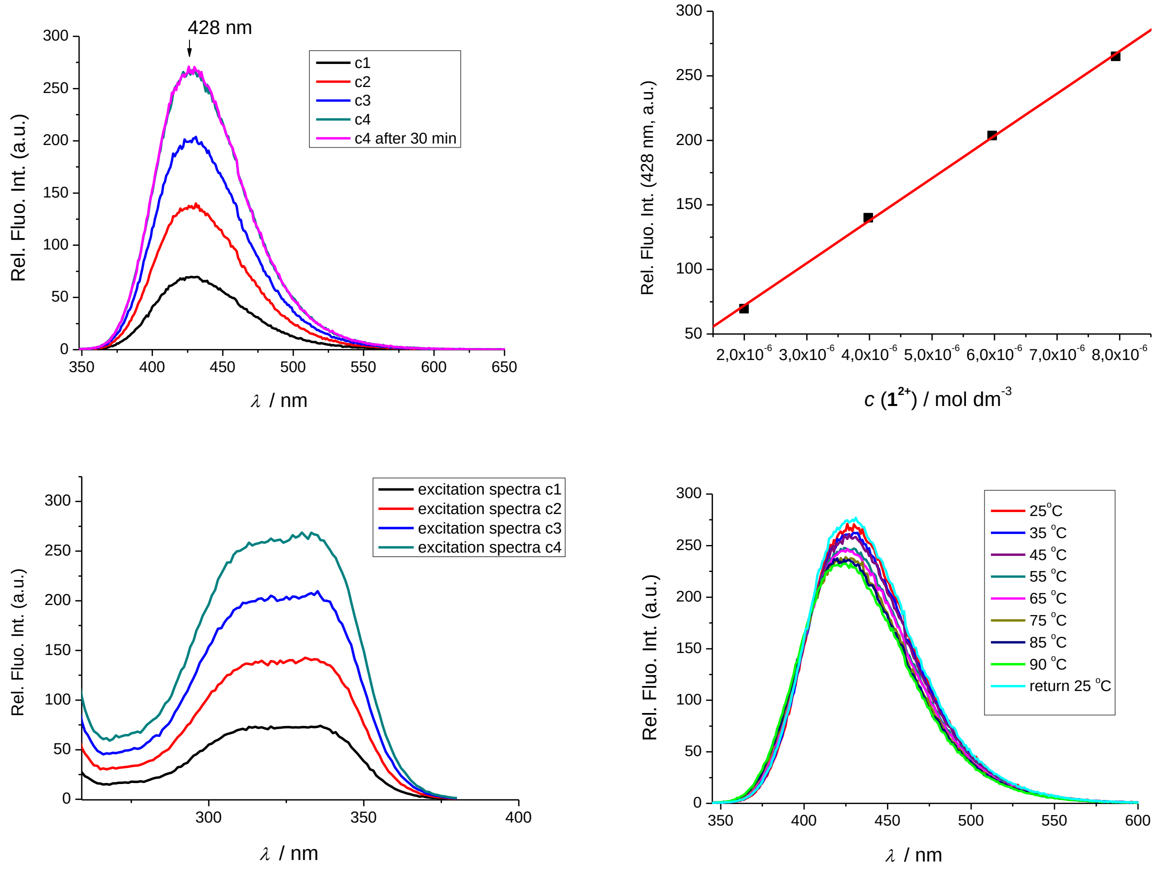


**Figure S26**. Top: Fluorescence spectra of $\mathbf{1}^{2+}$ ($\lambda_{ex}$ = 335 nm) at micromolar concentrations (left); linear dependence (—) of the fluorescence at 428 nm (■) on the $\mathbf{1}^{2+}$ concentration (right); bottom: Excitation spectra of $\mathbf{1}^{2+}$ (left); influence of temperature increase (T = 25°C – 90 °C) on fluorescence spectra of $\mathbf{1}^{2+}$ (c = $8\times10^{-6}$ M) (right). Done in buffer sodium cacodylate (pH = 7.0, *I* = 0.05 M).

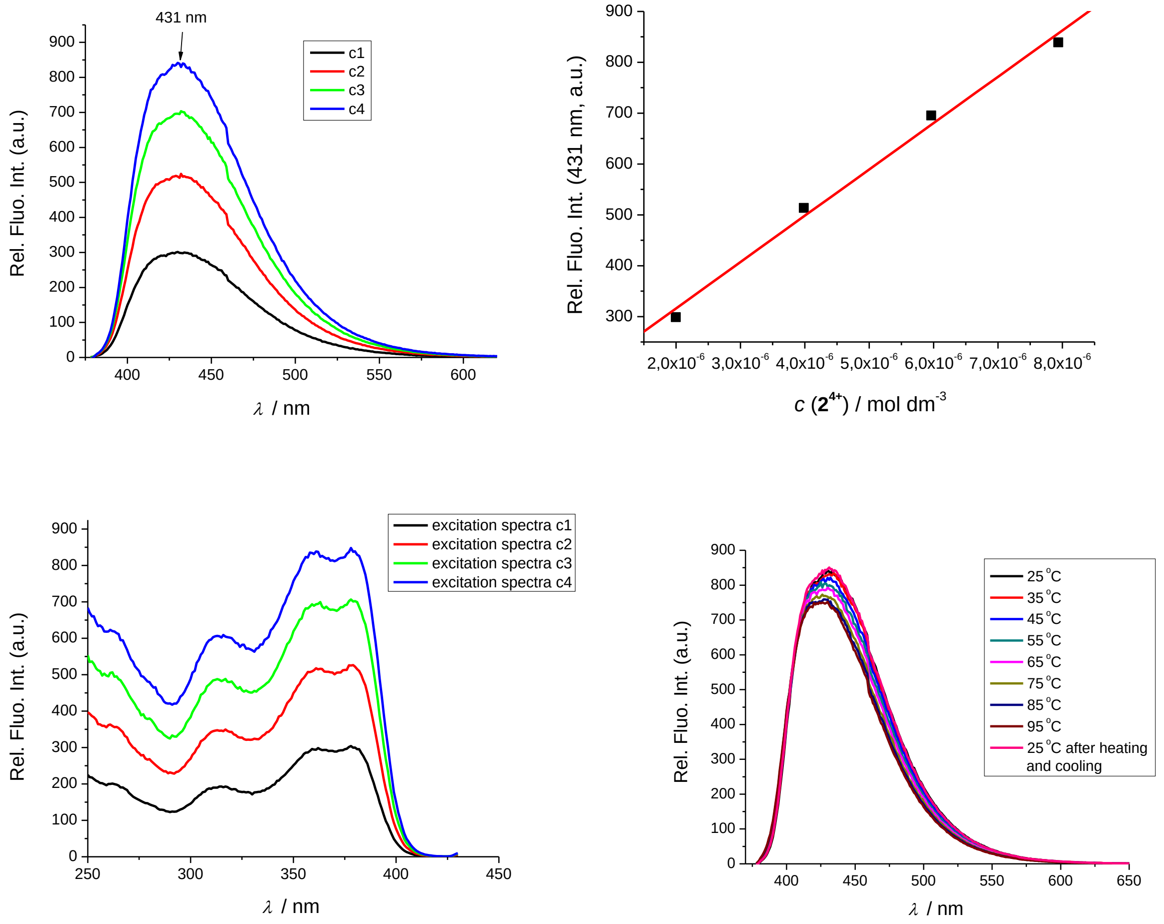


**Figure S27**. Top: Fluorescence spectra of $\mathbf{2}^{4+}$ ($\lambda_{ex}$ = 377 nm) at micromolar concentrations (left); linear dependence (—) of the fluorescence at 431 nm (■) on the $\mathbf{2}^{4+}$ concentration (right), bottom: Excitation spectra of $\mathbf{2}^{4+}$ (left); influence of temperature increase (T = 25°C – 90 °C) on fluorescence spectra of $\mathbf{2}^{4+}$ (c = 8×10$^{-6}$ M) (right)**.** Done in buffer sodium cacodylate (pH = 7.0, *I* = 0.05 M).

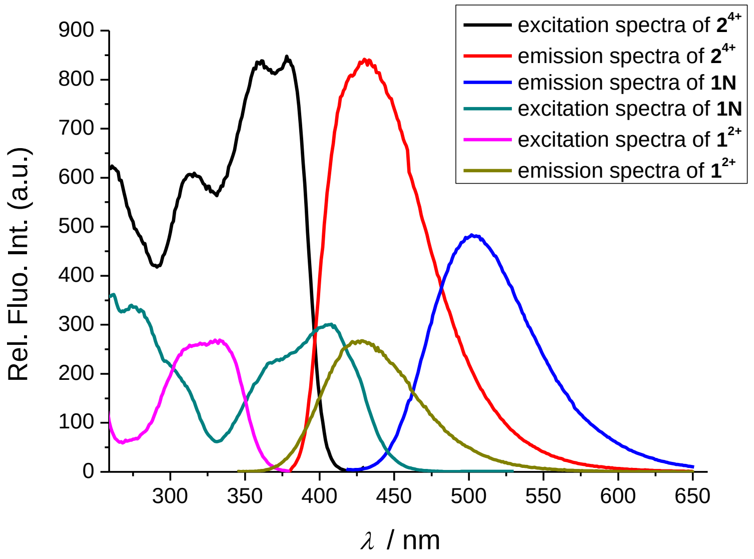


**Figure S28**. Comparison of emission and excitation spectra of **1N, $1^{2+}$** and **$2^{4+}$** in buffer sodium cacodylate (ph = 7.0, *I* = 0.05 M).

**Study of interactions with DNA/RNA/protein**

Polynucleotides were purchased as noted: poly dGdC – poly dGdC, poly dAdT – poly dAdT, poly A – poly U, poly A, poly G, poly C, poly U (Sigma), *calf thymus* (ct)-DNA (Aldrich) and dissolved in sodium cacodylate buffer, *I* = 0.05 M, pH = 7.0. The ct-DNA was additionally sonicated and filtered through a 0.45 mm filter to obtain mostly short (ca. 100 base pairs) rod-like B-helical DNA fragments.[8] The polynucleotide concentration was determined spectroscopically[9] as the concentration of phosphates (corresponds to *c*(nucleobase)).

Bovine Serum Albumin (BSA) (Sigma-Aldrich) was dissolved in sodium cacodylate buffer, *I* = 0.05 M, pH = 7.0 and its concentration determined spectroscopically using a NanoDrop spectrophotometer at 280 nm using its molar extinction coefficient 43824 $M^{-1}$ $cm^{-1}$.

**Table S2**. Groove widths and depths for selected nucleic acid conformations.[10],[11]

| Structure type | Groove width [Å] | | Groove depth [Å] | |
|---|---|---|---|---|
| | major | minor | major | minor |
| [a] poly rA – poly rU | 3.8 | 10.9 | 13.5 | 2.8 |
| [b] poly dA – poly dT | 11.4 | 3.3 | 7.5 | 7.9 |
| [c] poly dGdC – poly dGdC | 13.5 | 9.5 | 10.0 | 7.2 |
| [c] poly dAdT – poly dAdT | 11.2 | 6.3 | 8.5 | 7.5 |

[a] A-helical structure (e.g. A-DNA)
[b] C-helical structure (e.g. C-DNA)
[c] B- helical structure (e.g. B-DNA)

*Thermal melting experiments*

Thermal melting experiments were performed on a Varian Cary 100 Bio spectrometer in quartz cuvettes (1 cm). The measurements were done in aqueous buffer solution at pH = 7.0 (sodium cacodylate buffer *I* = 0.05 M). Thermal melting curves for ds-DNA, ds-RNA and their complexes with **1N**, $\mathbf{1^{2+}}$ and $\mathbf{2^{4+}}$ were determined by following the absorption change at 260 nm as a function of temperature.[12] *Tm* values are the midpoints of the transition curves determined from the maximum of the first derivative and checked graphically by the tangent method. The Δ*Tm* values were calculated

subtracting *Tm* of the free nucleic acid from *Tm* of the complex. Every Δ*Tm* value reported here was the average of at least two measurements. The error in Δ*Tm* is ± 0.5 °C.

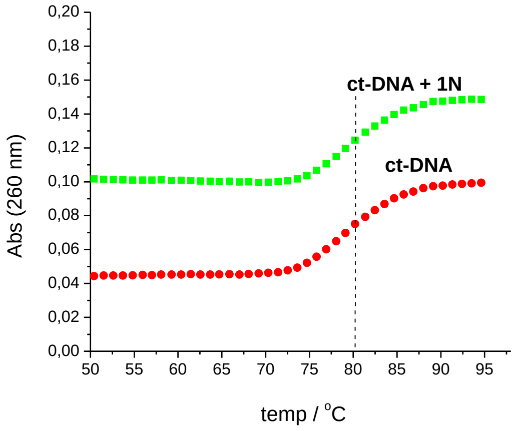


**Figure S29.** Thermal denaturation curves of ct-DNA ($c$(ct-DNA) = $2.5\times10^{-5}$ M, $r_{[\mathbf{1N}]\,/\,[\text{ct-DNA}]}$ = 0.1) at ph = 7.0 (sodium cacodylate buffer, $I$ = 0.05 M) upon addition of **1N**. Error in Δ*Tm* values: ±0.5 °C.

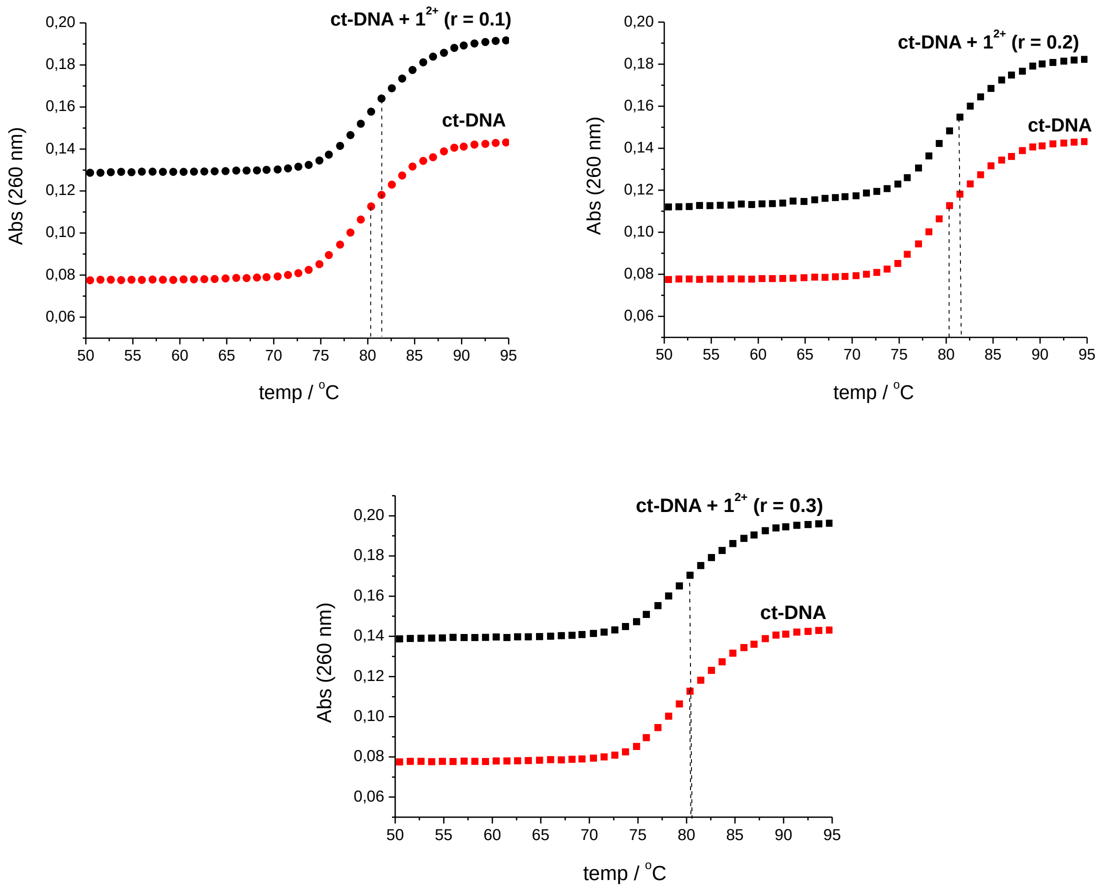


**Figure S30.** Thermal denaturation curves of ct-DNA ($c$(ct-DNA) = $2.5\times10^{-5}$ M, $r_{[\mathbf{1}^{2+}]/[\text{ct-DNA}]}$ = 0.1 – 0.3) at pH 7.0 (sodium cacodylate buffer, $I$ = 0.05 M) upon addition of $\mathbf{1}^{2+}$. Error in $\Delta Tm$ values: ±0.5 °C.

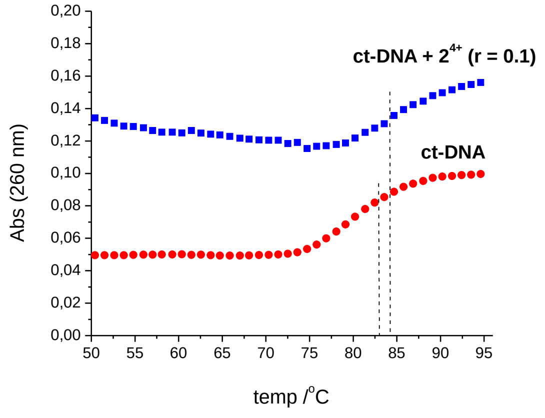


**Figure S31.** Thermal denaturation curves of ct-DNA ($c$(ct-DNA) = $2.5\times10^{-5}$ M, $r_{[\mathbf{2}^{4+}]/[\text{ct-DNA}]}$ = 0.1 – 0.3) at pH = 7.0 (sodium cacodylate buffer, $I$ = 0.05 M) upon addition of $\mathbf{2}^{4+}$. Error in $\Delta Tm$ values: ±0.5 °C.

**Table S3.** The [a]$\Delta$*Tm* values (°C ) of ctDNA upon addition of ratio [b]*r* of **1N, $1^{2+}$** and **$2^{4+}$** at pH = 7.0 (sodium cacodylate buffer, *I* = 0.05 M)

| [a]$\Delta$*Tm*/°C | [b]*r* = 0.1 | [b]*r* = 0.2 | [b]*r* = 0.3 |
|---|---|---|---|
| **1N** | - | - | 0 |
| **$1^{2+}$** | +1.1 | +1 | 0 |
| **$2^{4+}$** | +4 | c | c |

[a] Error in $\Delta$Tm : ±0.5°C;
[b] *r* = [compound] / [polynucleotide];
[c] precipitation of compound

**Spectrophotometric titrations**

*Fluorimetric titrations*

In fluorimetric experiments, an excitation wavelength of $\lambda_{ex}$ = 425 nm was used to avoid absorption of excitation light by added polynucleotides or BSA. Fluorimetric titrations were performed by adding portions of polynucleotide or BSA solution into the solution of the studied compound being studied (*c* = $5\times10^{-7}$ M). After mixing polynucleotides or BSA with the compound, equilibrium was reached in less than 120 s. Fluorescence spectra were collected using an excess of DNA/RNA ($r_{[\mathbf{compd}]/[DNA]}$ <0.3) to assure one dominant binding mode. To obtain binding constants (*Ks*), titration data were processed by means of non-linear fitting to the Scatchard equation (McGhee, von Hippel formalism),[13] which gave values of the ratio of [bound compound] / [polynucleotide] in the range 0.1–0.3, but for easier comparison, all *Ks* values were re-calculated for the fixed n = 0.25 (for ds-DNA/RNA) or 0.5 (for ss-RNA). Calculated values for *Ks* have satisfactory correlation coefficients (>0.99). Titration data with BSA gave an excellent correlation (>0.999) to non-linear regression fitting to a 1:1 (**compd**:BSA) stoichiometry model, giving a value of *Ks*.

For fluorimetric titrations fluorescence spectra were recorded on Varian Cary Eclipse fluorimeter in quartz cuvettes (1 cm) by adding portions of polynucleotide or protein solution into the solution of the studied compound (c = $5\times10^{-7}$ M for **1N** and **$1^{2+}$** and $5\times10^{-8}$ M for **$2^{4+}$**).

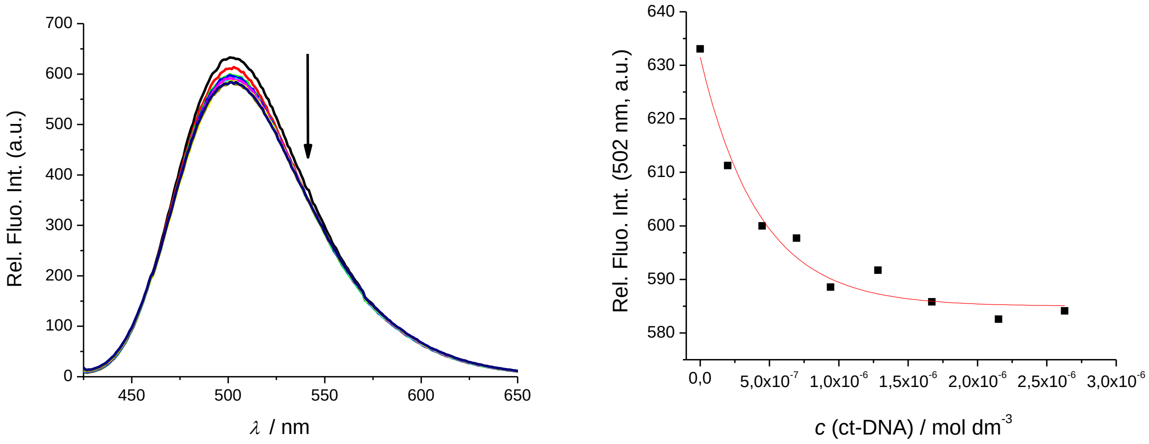


**Figure S32.** LEFT: Fluorimetric titration of **1N** ($c$ = 5×10$^{-7}$ M; $\lambda_{ex}$ = 408 nm) with ctDNA at pH = 7, sodium cacodylate buffer, $I$ = 0.05 M. RIGHT: dependence of fluorescence at $\lambda_{max}$= 502 nm on $c$(DNA), red line is non-linear least square fitting of Scatchard eq. (McGhee, von Hippel formalism)[13] to the experimental data.

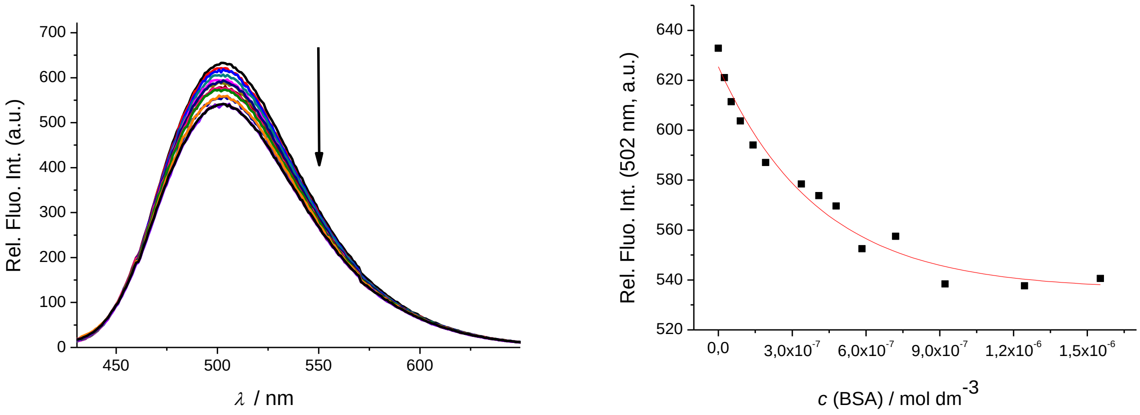


**Figure S33.** LEFT: Fluorimetric titration of **1N** ($c$ = 5×10$^{-7}$ M; $\lambda_{ex}$ = 408 nm) with BSA (bovine serum albumin) at pH = 7, sodium cacodylate buffer, $I$ = 0.05 M. RIGHT: dependence of fluorescence at $\lambda_{max}$= 502 nm on $c$(BSA), red line is the non-linear least square fitting to the experimental data for 1:1 = **1N**:BSA stoichiometry.

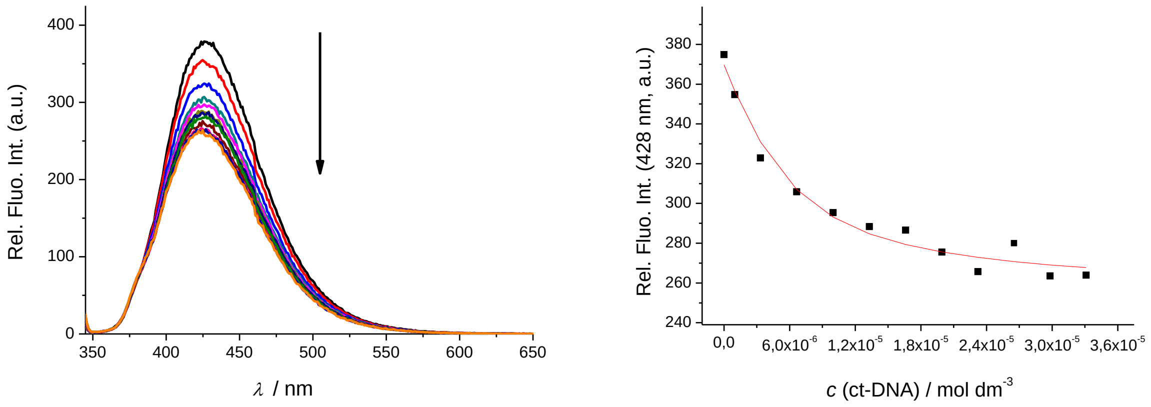


**Figure S34**. LEFT: Fluorimetric titration of $\mathbf{1}^{2+}$ ($c$ = 5×10$^{-7}$ M; $\lambda_{ex}$ = 335 nm) with ct-DNA at pH = 7, sodium cacodylate buffer, $I$ = 0.05 M. RIGHT: dependence of fluorescence at $\lambda_{max}$= 428 nm on $c$(DNA), red line is non-linear least square fitting of Scatchard eq. (McGhee, von Hippel formalism)[13] to the experimental data.

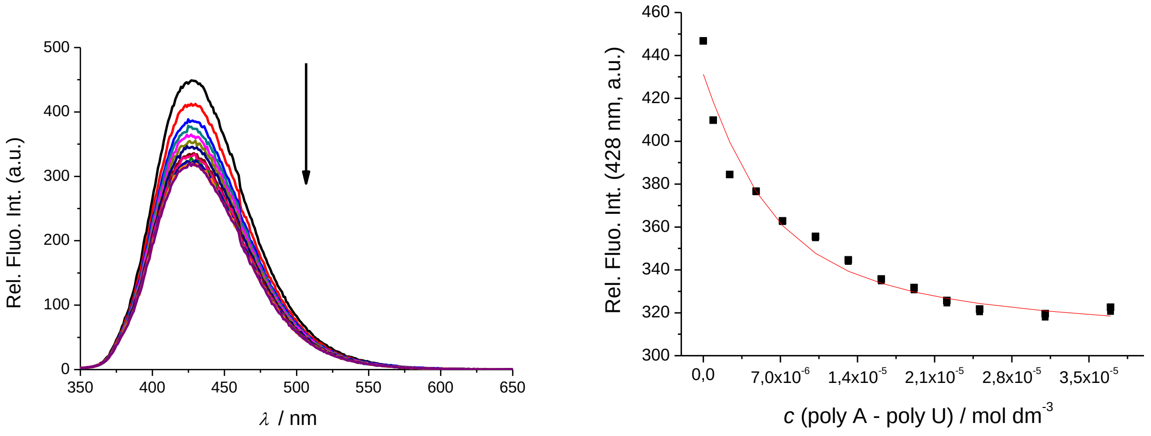


**Figure S35.** LEFT: Fluorimetric titration of $\mathbf{1}^{2+}$ ($c$ = 5×10$^{-7}$ M; $\lambda_{ex}$ = 335 nm) with poly A – poly U at pH = 7, sodium cacodylate buffer, $I$ = 0.05 M. RIGHT: dependence of fluorescence at $\lambda_{max}$ = 428 nm on $c$(RNA), red line is non-linear least square fitting of Scatchard eq. (McGhee, von Hippel formalism)[13] to the experimental data.

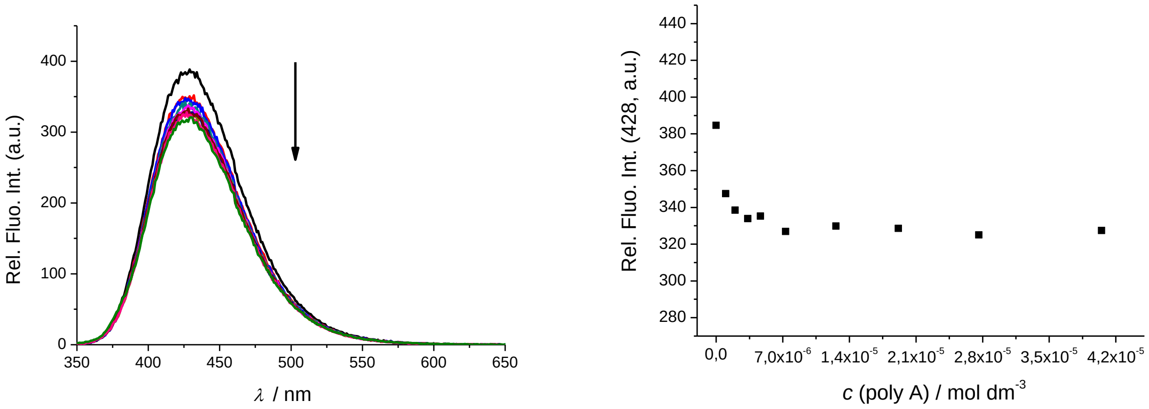


**Figure S36.** LEFT: Fluorimetric titration of $\mathbf{1}^{2+}$ ($c$ = 5×10$^{-7}$ M; $\lambda_{ex}$ = 335 nm) with poly A at pH = 7, sodium cacodylate buffer, $I$ = 0.05 M. RIGHT: dependence of fluorescence at $\lambda_{max}$ = 428 nm on $c$(RNA).

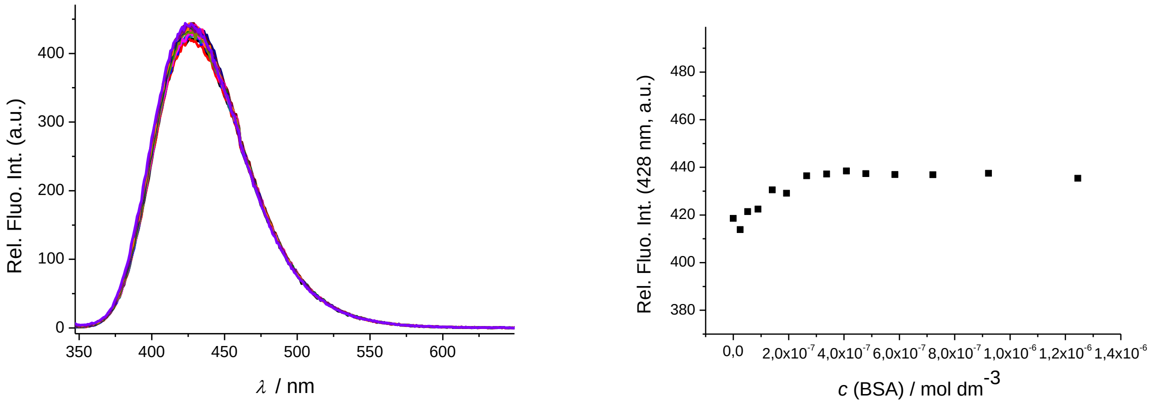


**Figure S37.** LEFT: Fluorimetric titration of $\mathbf{1}^{2+}$ ($c$ = 5×10$^{-7}$ M; $\lambda_{ex}$ = 335 nm) with BSA (bovine serum albumin) at pH = 7, sodium cacodylate buffer, $I$ = 0.05 M. RIGHT: dependence of fluorescence at $\lambda_{max}$ = 428 nm on $c$(BSA).

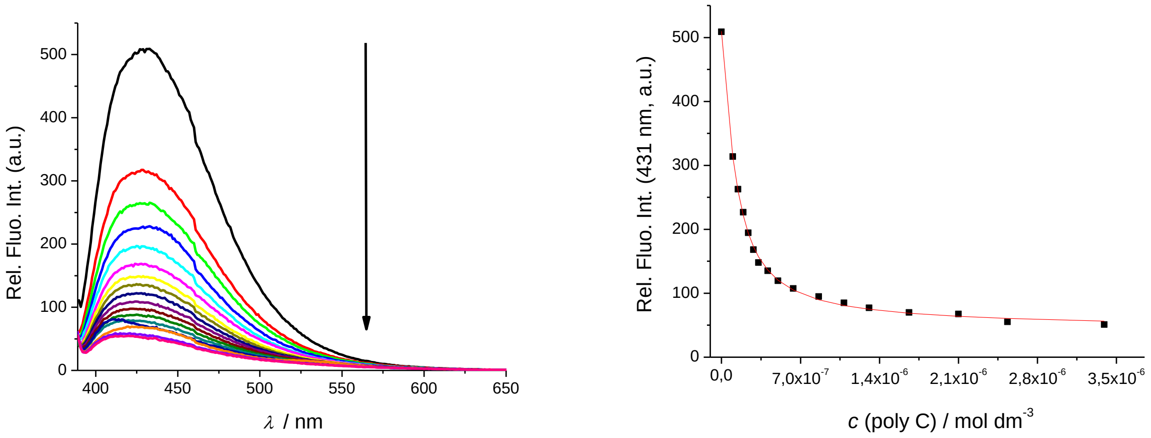


**Figure S38.** Left: Fluorimetric titration of $\mathbf{2}^{4+}$ ($c$ = 5×10$^{-8}$ M; $\lambda_{ex}$ = 377 nm) with poly C at pH = 7, sodium cacodylate buffer, $I$ = 0.05 M. Right: Dependence of fluorescence at $\lambda_{max}$ = 431 nm on $c$(RNA), red line is the non-linear least square fitting of Scatchard eq. (McGhee, von Hippel formalism)[13] to the experimental data.

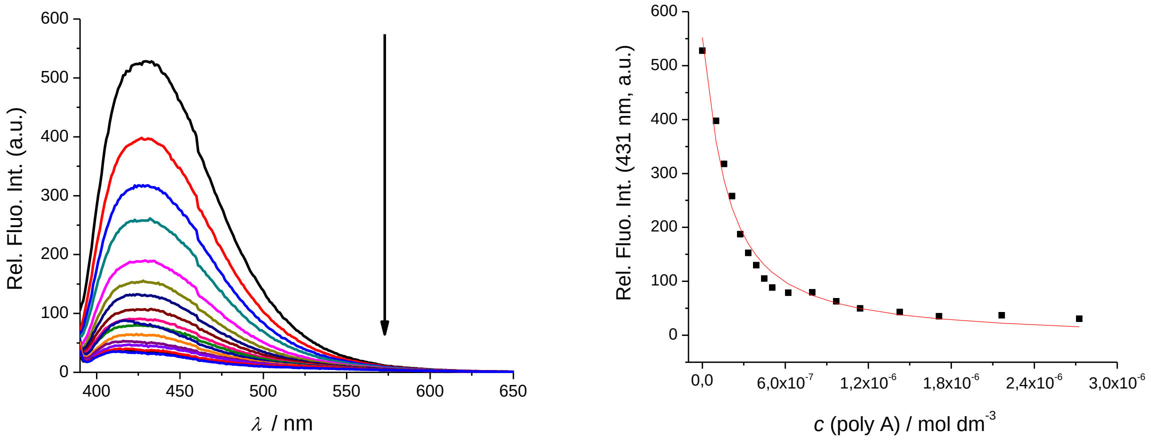


**Figure S39**. Fluorimetric titration of $\mathbf{2}^{4+}$ ($c$ = 5×10$^{-8}$ M; $\lambda_{ex}$ = 377 nm) with poly A at pH = 7, sodium cacodylate buffer, $I$ = 0.05 M. Right: Dependence of fluorescence at $\lambda_{max}$ = 431 nm on $c$(RNA), red line is the non-linear least square fitting of Scatchard eq. (McGhee, von Hippel formalism)[13] to the experimental data.

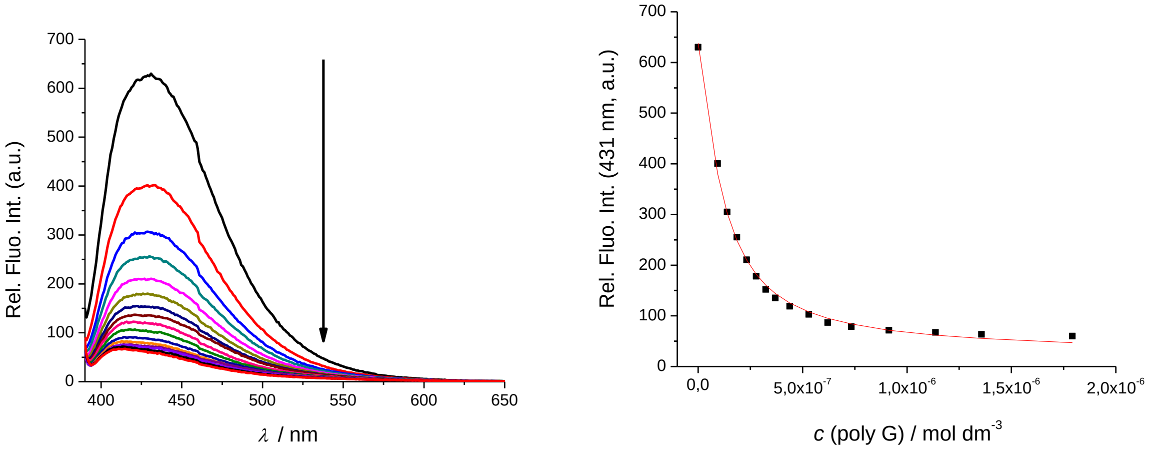


**Figure S40.** Fluorimetric titration of $\mathbf{2}^{4+}$ ($c$ = 5×10$^{-8}$ M; $\lambda_{ex}$ = 377 nm) with poly G at pH = 7, sodium cacodylate buffer, $I$ = 0.05 M. Right: Dependence of fluorescence at $\lambda_{max}$ = 431 nm on $c$(RNA), red line is the non-linear least square fitting of Scatchard eq. (McGhee, von Hippel formalism)[13] to the experimental data.

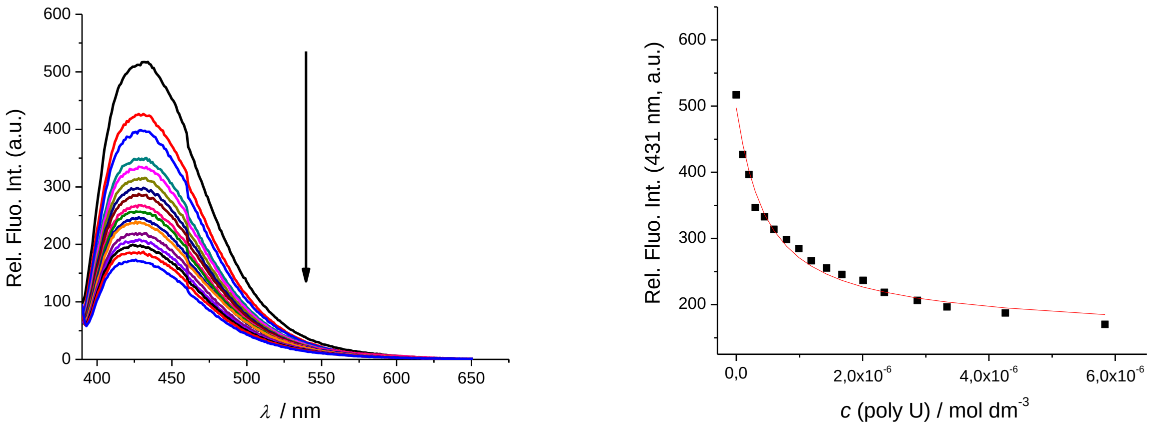


**Figure S41**. Fluorimetric titration of $\mathbf{2}^{4+}$ ($c$ = 5×10$^{-8}$ M; $\lambda_{ex}$ = 377 nm) with poly U at pH = 7, sodium cacodylate buffer, $I$ = 0.05 M. Right: Dependence of fluorescence at $\lambda_{max}$ = 431 nm on $c$(RNA), red line is the non-linear least square fitting of Scatchard eq. (McGhee, von Hippel formalism)[13] to the experimental data.

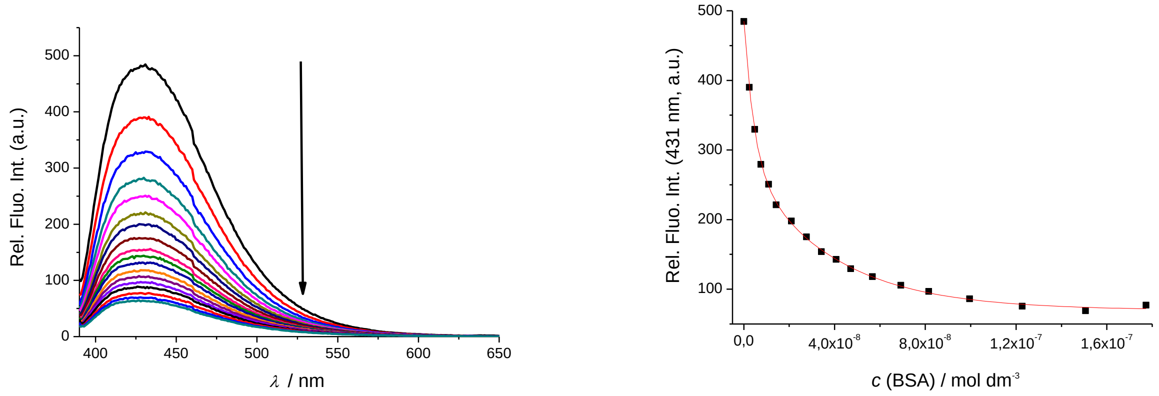


**Figure S42**. Fluorimetric titration of $\mathbf{2}^{4+}$ ($c$ = 5×10$^{-8}$ M; $\lambda_{ex}$ = 377 nm) with BSA (bovine serum albumin) at pH = 7, sodium cacodylate buffer, $I$ = 0.05 M. Right: Dependence of fluorescence at $\lambda_{max}$ = 431 nm on $c$(BSA), red line is the non-linear least square fitting of Scatchard eq. (McGhee, von Hippel formalism)[13] to the experimental data for 1:1 = $\mathbf{2}^{4+}$:BSA stoichiometry.

*A competitive Cl-6-TOTO-analogue / 2$^{4+}$ displacement titration from ct-DNA*

Fluorescence emission was measured on a Varian Cary Eclipse spectrophotometer at 25°C, pH 7.0, using sodium cacodylate buffer, $I$ = 0.05 M.

A buffered solution of Cl-6-TOTO ($c$ = 5×10$^{-7}$ mol dm$^{-3}$) was non-fluorescent (Figure S43, Left, green line) but upon addition of $c$(ct-DNA) = 2×10$^{-6}$ mol dm$^{-3}$ and excitation at 483 nm, gave rise to fluorescence emission at 533 nm (Figure S43, Left, red line), in accordance with previous results.[14]

The series of aliquots of $\mathbf{2}^{4+}$ stock solution ($c$ = 4×10$^{-3}$ mol dm$^{-3}$) were then subsequently added to the DNA-TOTO solution, and Cl-6-TOTO emission was monitored (Figure S43, Left, c1-c8). Processing of the titration data (Figure S43, Right) revealed the $IC_{50}$ = 0.11 value (ratio c(TOTO)/c($\mathbf{2}^{4+}$) at which 50% of the emission at 533 nm is quenched).

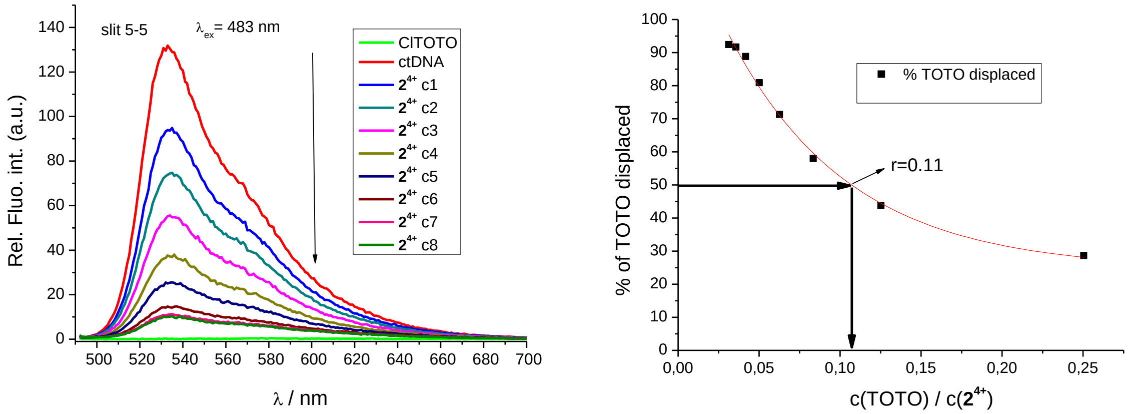


**Figure S43.** Left: Fluorimetric titration of Cl-6-TOTO/ct-DNA complex (*c* (Cl-6-TOTO) = $5\times10^{-7}$ mol $dm^{-3}$; c(ct-DNA) = $2\times10^{-6}$ mol $dm^{-3}$, $\lambda_{exc}$= 483 nm) with $\mathbf{2}^{4+}$. Right: dependence of the % of Cl-6-TOTO displaced ((Int(TOTO/DNA)-Int(TOTOfree) / Int(TOTO/DNA))x100) on the ratio c(TOTO)/c($\mathbf{2}^{4+}$). Titration performed at pH 7.0, sodium cacodylate buffer, *I* = 0.05 M.

It should be noted that Cl-6-TOTO showed some interaction with $\mathbf{2}^{4+}$, but only at 10-fold larger excess than used in the displacement experiments. Nevertheless, even such a small contribution should be taken into account for the competition experiment performed.

## Circular dichroism (CD) experiments

CD spectra were recorded on a JASCO J815 spectrophotometer at room temperature using appropriate 1 cm path quartz cuvettes with scanning speed of 200 nm/min. A background spectrum of the buffer was subtracted from each spectrum and each spectrum was the result of three accumulations. CD experiments were performed by adding portions of compound stock solution into the solution of polynucleotide (c = $2\times10^{-5}$ M).

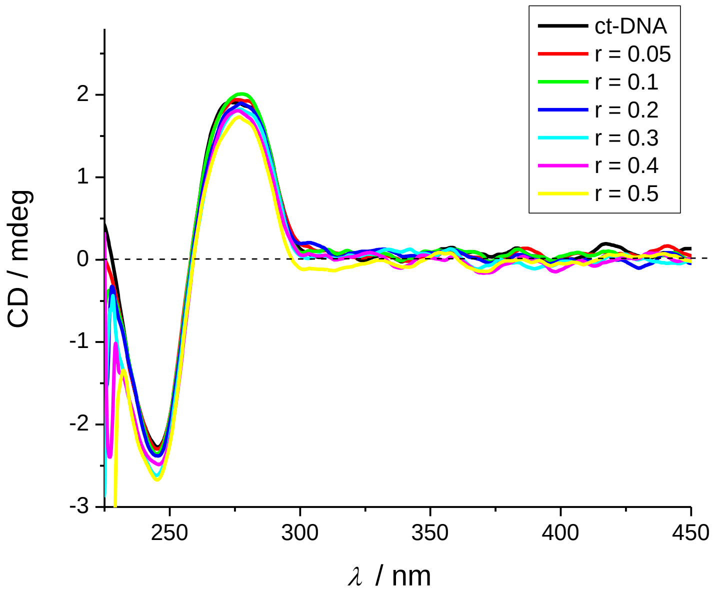


**Figure S44.** CD titration of ct-DNA ($c = 2\times10^{-5}$ M) with **1N** at molar ratios $r$ = [compound] / [polynucleotide] (pH = 7.0, buffer sodium cacodylate, $I$ = 0.05 M).

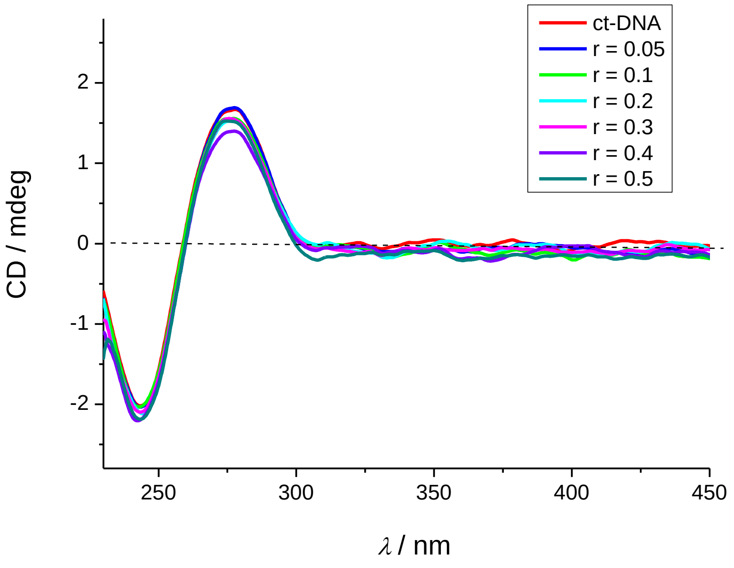


**Figure S45**. CD titration of ct-DNA ($c$ = 2×10$^{-5}$ M) with $\mathbf{1^{2+}}$ at molar ratios $r$ = [compound] / [polynucleotide] (pH = 7.0, buffer sodium cacodylate, $I$ = 0.05 M).

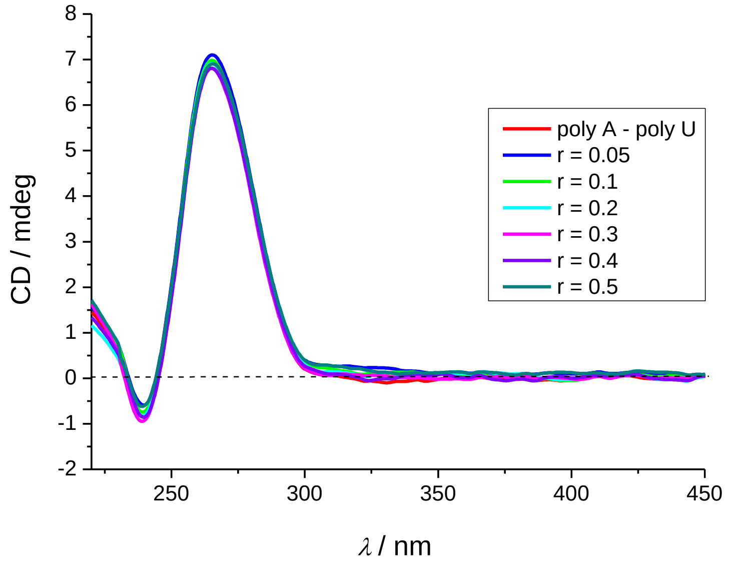


**Figure S46**. CD titration of poly A – poly U ($c$ = 2×10$^{-5}$ M) with $\mathbf{1^{2+}}$ at molar ratios $r$ = [compound] / [polynucleotide] (pH = 7.0, buffer sodium cacodylate, $I$ = 0.05 M).

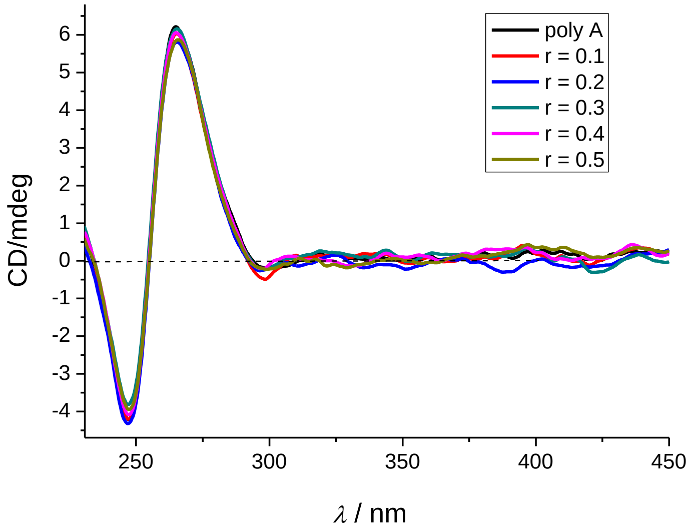


**Figure S47**. CD titration of poly A ($c$ = 2×10$^{-5}$ M) with $\mathbf{1}^{2+}$ at molar ratios $r$ = [compound] / [polynucleotide] (pH = 7.0, buffer sodium cacodylate, $I$ = 0.05 M).

**Raman and SERS measurements**

Raman and SERS spectra were acquired using the portable Raman system with a PD-LD (Necsel) BlueBox laser source (excitation wavelength 785 nm) equipped with a B&W-Tek fiber optic Raman BAC102 probe and coupled with Maya2000Pro (OceanOptics) spectrometer. Laser radiation of 480 mW and 425 mW power for Raman and SERS measurements, respectively, was focused on a sample in a quartz cuvette. The spectra were measured in the 4000–400 $cm^{-1}$ range at the spectral resolution of 4 $cm^{-1}$. For each spectrum 50 scans were accumulated using an exposure time of 12 s (Raman) and 8 s (SERS).

The stock solutions of $\mathbf{2}^{4+}$ ($1\times10^{-4}$ M and $1\times10^{-5}$ M) were prepared in water and aqueous buffer solution (pH = 7.0, $I$ = 0.05 M, sodium cacodylate/HCl buffer). In addition, by diluting the appropriate volume of the stock solution ($1\times10^{-4}$ M) with the buffer, a buffered solution of $5\times10^{-5}$ M $\mathbf{2}^{4+}$ was prepared for the Raman measurement.

Surface-enhanced Raman scattering was measured in the silver colloidal suspension, prepared by reduction of silver nitrate with trisodium citrate according to the modified Lee and Meisel method.[15] It contained silver nanospheres of around 100 nm diameter, stabilized by the surface citrate anions. The resulting colloidal suspension was gray colored, characterized by a maximum at 417 nm in the UV/Vis spectrum, pointing to the typical silver plasmon resonance frequency. The pH value of the silver colloid was 8.7.

The samples for concentration dependent SERS measurements, $c(\mathbf{2}^{4+}) = 5\times10^{-8}$ M, $1\times10^{-7}$ M, $5\times10^{-7}$ M, $1\times10^{-6}$ M and $5\times10^{-6}$ M, were prepared by diluting appropriate volume of $\mathbf{2}^{4+}$ solution ($1\times10^{-5}$ M) in Milli-Q water, followed by addition of the silver colloid. In case of the buffered working samples, $c(\mathbf{2}^{4+}) = 5\times10^{-8}$ M and $1\times10^{-6}$ M, an appropriate volume of the buffered $\mathbf{2}^{4+}$ stock solution ($1\times10^{-5}$ M) was diluted with Na-cacodylate buffer and then the silver colloid was added. The samples $\mathbf{2}^{4+}$/ct-DNA and $\mathbf{2}^{4+}$/BSA complexes were prepared in molar ratios of 1, 0.2 and 0.1, by mixing appropriate volumes of the respective buffered stock solutions, subsequently adding the silver colloid. The samples of the complexes with the nucleic acid were of final $\mathbf{2}^{4+}$ concentrations $5\times10^{-8}$ M and $1\times10^{-6}$ M, while in the samples containing protein the final $\mathbf{2}^{4+}$ concentration was $1\times10^{-6}$ M. All the samples for the SERS measurements contained the same volume ratio of the silver colloid, 80% v/v.

**Table S4**. Assignment of the bands in the Raman and SERS spectra of **2**$^{4+}$, $c$ = 1×10$^{-4}$ M (Raman) and $c$ = 1×10$^{-6}$ M (SERS), in the absence and presence of Na-cacodylate buffer, pH = 7.0. SERS spectra were measured in a silver colloid, with an excitation wavelength 785 nm.

| **Wavenumber / cm$^{-1}$** | | | | **Assignment** |
|---|---|---|---|---|
| **Raman** | | **SERS** | | |
| **1×10$^{-4}$ M water** | **1×10$^{-4}$ M buffer** | **1×10$^{-6}$ M water** | **1×10$^{-6}$ M buffer** | |
| 3224 | 3224 | 3221 | 3192 | $\nu$ H–O–H |
| | 2934 | 2930 | 2930 | $\nu_{as}$ C–H ($CH_3$) |
| 2221 | 2218 | 2215 | 2215 | $\nu$ C≡C |
| 1637 | 1637 | | | $\delta$ $H_2O$ |
| 1601 | 1602 | 1596 | 1595 | $\nu$ CC (phenyl) |
| | 1414 | | 1453 | $\delta_{as}$ $CH_3$ |
| | | 1385 | 1384 | $\delta_{s}$ $CH_3$ |
| 1354 | 1350 | 1354 | 1353 | $\nu$ C≡C~~$\delta$ CNC (–N$(CH_3)_3$$^+$)~~ + $\nu$ CC (phenyl) |
| | | 1290 | 1286 | $\nu$ CC (phenyl) |
| | | | | $\nu_{s}$ –$CF_3$ ($CF_3SO_3^-$) |
| | | 1130 | | $\nu_{s}$ –$SO_3$ ($CF_3SO_3^-$) |
| 1073 | 1071 | 1070 | 1071 | $\nu$ B–$(C_{ar})_3$ |
| | | 1033 | 1033 | $\delta_{ip}$ CH (phenyl) |
| | | 995 | 995 | $\nu$ CC (phenyl “breathing”) |
| | | 784 | 784 | $\delta_{oop}$ CH (phenyl) |
| | | 730 | 724 | $\delta$ CC (phenyl) |
| | 605 | | | $\nu$ As=O |
| | | 576 | 576 | $\delta$ CC (phenyl) |
| | | | 231 | $\nu$ Ag–Cl |
| | | 216 | | $\nu$ Ag–N |

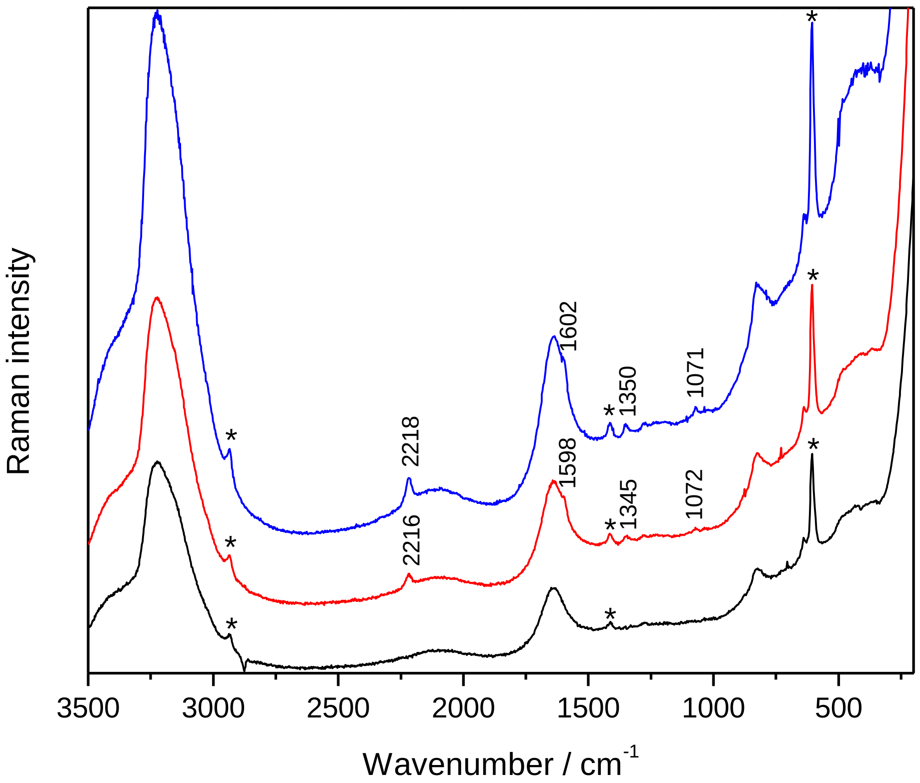


**Figure S48**. Raman spectra of Na-cacodylate buffer, pH = 7.0 (black line) and **2**$^{4+}$, $c$ = 1×10$^{-4}$ M (blue line) and $c$ = 5×10$^{-5}$ M (red line), in Na-cacodylate buffer, pH = 7.0. Excitation wavelength 785 nm. The bands labeled with asterisk originate from the buffer. The spectra are displaced for visual clarity.

**Biological Screening**

*MTT assay:*

The effect of **$2^{4+}$** on cell proliferation was evaluated by MTT test. The 5 x $10^3$ HeLa and HEK 293 cells (human cervical carcinoma and human embryonic kidney cell line, respectively) were incubated in 96-well plates, and after 24 h, the compound **$2^{4+}$** was added in a range of concentrations ($1x10^{-4}$ – $1x10^{-8}$ M). After 72 h incubation with the compound solutions, MTT dissolved in medium was added directly in the wells at final concentration of 0.5 mg/ml. Cells were incubated at 37°C for another 2h to allow the formation of insoluble formazane. The medium was discarded, cells were washed twice with PBS, and formazan was solubilized with 100μl of DMSO for 10 minutes with shaking. The results were collected on a 96 plate reader. The measurements were taken in quadruplicates.

*Confocal Laser Scanning Microscopy (CLSM):*

Leica SP8X FLIM was used for fluorescence experiments in live-cell imaging (HeLa cells). The images were processed in LAS X Leica Microsystems software package. The cells were seeded into 4-chamber 35 mm glass bottom petri dishes (Cellvis, Mountain View, USA) and incubated overnight allowing them to attach to the glass bottom of the petri dish. The concentration of the compound **$2^{4+}$** was $1x10^{-6}$ M and the compound was added 2 h prior to the imaging. The excitation was set to $\lambda_{ex}$ = 360-400 nm; the fluorescence emission was detected at $\lambda_{em}$ = 415-471 nm.

**Theoretical Studies**

All calculations (DFT and TD-DFT) were carried out with the program package Gaussian 09 (9.E.01)[16] and were performed on a parallel cluster system. GaussView (6.0.16) was used to visualize the results, to measure calculated structural parameters, and to plot orbital surfaces (isovalue: ± 0.020 [e $a_0^{-3}$]$^{1/2}$). Processing and evaluation of the calculations was carried out with multiwfn.[17] The ground-state geometries were optimized using the B3LYP functional[18] in combination with the 6-31+g(d) basis set.[19] The polarizable continuum model (PCM)[20] was used to include solvent effects for the ground state structure. Frequency calculation were performed on the optimized structures to confirm them to be local minima showing no negative (imaginary) frequencies. Based on these optimized structures, the lowest-energy vertical transitions (gas-phase and solvent correction using the polarizable continuum model) were calculated (singlets, 25 states) by TD-DFT, using the Coulomb attenuated functional CAM-B3LYP.[21] The CAM-B3LYP has been shown to more accurately describe ICT systems in comparison to B3LYP The ultrafine integration grid and no symmetry constraints were used for all molecules.

## TD-DFT calculations

## Compound $2^{4+}$

<table>
<tr><th>Calculated absorption spectra</th><th>Orbitals relevant to the $S_1 \leftarrow S_o$ transition</th><th>Orbitals relevant to the $S_4 \leftarrow S_o$ and $S_5 \leftarrow S_o$ transition</th></tr>
<tr><td>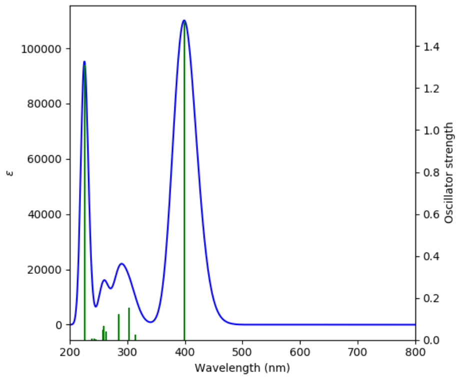

TD-DFT CAM-B3LYP/6-31+G(d), gas phase</td><td>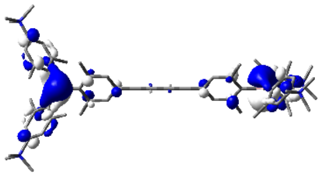
LUMO+1<br>–7.299 eV (gas phase, CAM-B3LYP)<br>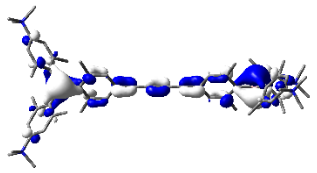
LUMO<br>–7.510 eV (gas phase, CAM-B3LYP)<br>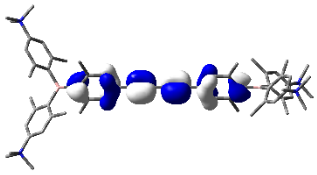
HOMO<br>–12.629 eV (gas phase, CAM-B3LYP)<br>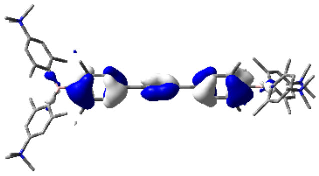
HOMO-4<br>–14.030 eV (gas phase, CAM-B3LYP)</td><td>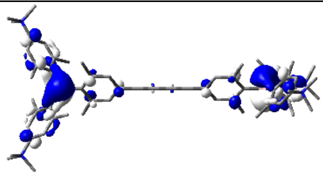
LUMO+1<br>–7.299 eV (gas phase, CAM-B3LYP)<br>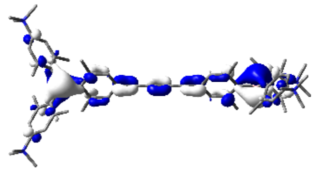
LUMO<br>–7.510 eV (gas phase, CAM-B3LYP)<br>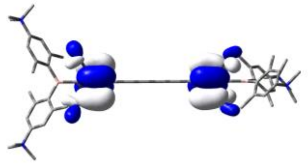
HOMO-2<br>–13.903 eV (gas phase, CAM-B3LYP)<br>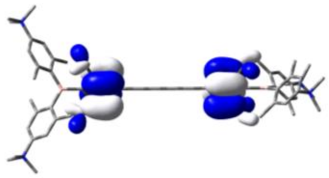
HOMO-3<br>–13.904 eV (gas phase, CAM-B3LYP)</td></tr>
</table>

| Orbitals relevant to the $S_6 \leftarrow S_o$ transition |
| --- |
| 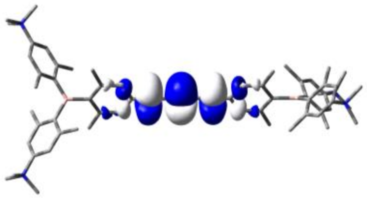<br>**LUMO+10**<br>**–4.631 eV (gas phase, CAM-B3LYP)** |
| 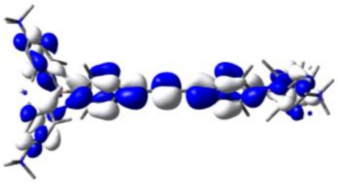<br>**LUMO+2**<br>**–6.319 eV (gas phase, CAM-B3LYP)** |
| 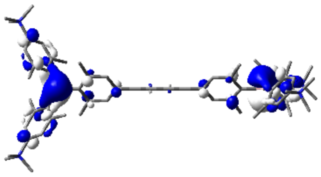<br>**LUMO+1**<br>**–7.299 eV (gas phase, CAM-B3LYP)** |
| 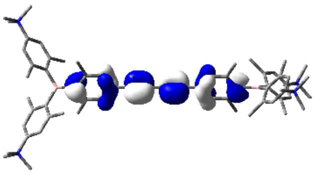<br>**HOMO**<br>**–12.629 eV (gas phase, CAM-B3LYP)** |
| 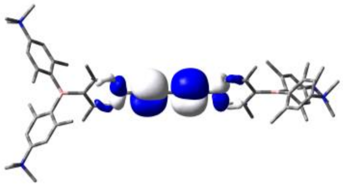<br>**HOMO-1**<br>**–13.632 eV (gas phase, CAM-B3LYP)** |
| 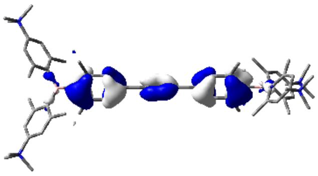<br>**HOMO-4**<br>**–14.030 eV (gas phase, CAM-B3LYP)** |

**Table S5:** Lowest energy singlet electronic transition of $\mathbf{2}^{4+}$ (TD-DFT CAM-B3LYP/6-31+G(d), gas phase)

| State | E [eV] | λ [nm] | *f* | Major contributions |
|---|---|---|---|---|
| 1 | 3.11 | 398.97 | 1.5196 | H-4->L+1 (10%), HOMO->LUMO (77%) |
| 2 | 3.51 | 353.02 | 0.0000 | H-4->LUMO (21%), HOMO->L+1 (67%) |
| 3 | 3.64 | 340.32 | 0.0000 | H-1->LUMO (52%), H-1->L+2 (35%) |
| 4 | 3.94 | 314.79 | 0.0010 | H-3->LUMO (55%), H-2->L+1 (35%) |
| 5 | 3.94 | 314.72 | 0.0270 | H-3->L+1 (35%), H-2->LUMO (55%) |
| 6 | 4.09 | 303.01 | 0.1549 | H-4->L+1 (16%), H-1->L+10 (10%), HOMO->L+2 (49%) |
| 7 | 4.34 | 285.82 | 0.1141 | H-6->L+1 (41%), H-5->LUMO (44%) |
| 8 | 4.34 | 285.82 | 0.1245 | H-6->LUMO (44%), H-5->L+1 (41%) |

## Calculated Raman Spectrum

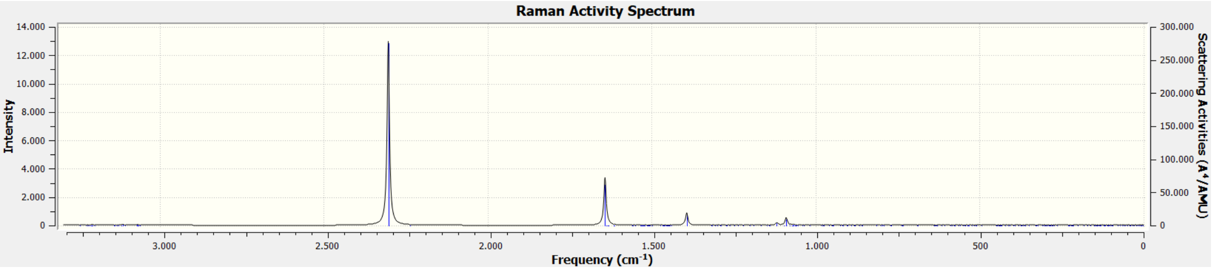


**Figure S49.** Calculated Raman spectrum of $\mathbf{2}^{4+}$ with significant signals at 2304 $cm^{-1}$, 1642 $cm^{-1}$, 1393 $cm^{-1}$ and 1090 $cm^{-1}$. For the combination of functional and basis set used, a correction factor of 0.96 should be applied.[21]

## Theoretical calculation: Cartesian coordinates

## Compound $2^{4+}$

DFT B3LYP/6-31G+g(d), gas phase, $S_0$

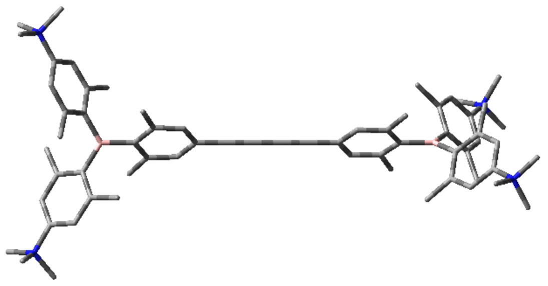

Point group: $C_2$

Total energy: - 1,728,884.69 kcal $mol^{-1}$

Dipole moment: 0 D

Immaginary frequencies: 0

| | | | |
|---|---|---|---|
| C | -0.000003 | 3.322365 | 0.000055 |
| C | -0.043726 | 4.044361 | -1.205805 |
| C | -0.063563 | 5.438417 | -1.222267 |
| C | -0.000003 | 6.176841 | 0.000013 |
| C | 0.063559 | 5.438453 | 1.222314 |
| C | 0.043721 | 4.044397 | 1.205894 |
| B | -0.000001 | 7.750446 | -0.000010 |
| C | 1.039915 | 8.567935 | 0.904448 |
| C | -1.039934 | 8.567910 | -0.904470 |
| C | 0.617308 | 9.582575 | 1.799579 |
| C | 1.548449 | 10.280977 | 2.585849 |
| C | 2.904532 | 10.003054 | 2.476382 |
| C | 3.345279 | 9.021292 | 1.592700 |
| C | 2.433255 | 8.293589 | 0.818861 |
| C | -0.617350 | 9.582555 | -1.799605 |
| C | -1.548506 | 10.280930 | -2.585882 |
| C | -2.904583 | 10.002976 | -2.476415 |
| C | -3.345308 | 9.021209 | -1.592727 |
| C | -2.433267 | 8.293531 | -0.818884 |
| C | 3.007138 | 7.226553 | -0.092144 |
| C | -0.840370 | 9.956572 | 1.979555 |
| C | 0.840321 | 9.956583 | -1.979580 |
| C | -3.007124 | 7.226482 | 0.092123 |
| C | 0.114345 | 6.099031 | 2.587970 |
| C | -0.114344 | 6.098953 | -2.587944 |
| N | 3.934208 | 10.744394 | 3.303947 |
| N | -3.934277 | 10.744288 | -3.303983 |
| C | -4.906472 | 11.465456 | -2.388158 |
| C | -3.320518 | 11.778994 | -4.218656 |
| C | 4.906389 | 11.465578 | 2.388119 |
| C | 3.320426 | 11.779093 | 4.218612 |
| C | -0.000002 | 1.902100 | 0.000075 |
| C | -0.000002 | 0.679503 | 0.000093 |
| C | 0.000003 | -3.322365 | 0.000055 |
| C | 0.043726 | -4.044361 | -1.205805 |
| C | 0.063563 | -5.438417 | -1.222267 |
| C | 0.000003 | -6.176841 | 0.000013 |
| C | -0.063559 | -5.438453 | 1.222314 |
| C | -0.043721 | -4.044397 | 1.205894 |
| B | 0.000001 | -7.750446 | -0.000010 |
| C | -1.039915 | -8.567935 | 0.904448 |
| C | 1.039934 | -8.567910 | -0.904470 |
| C | -0.617308 | -9.582575 | 1.799579 |

| | | | |
|---|---|---|---|
| C | -1.548449 | -10.280977 | 2.585849 |
| C | -2.904532 | -10.003054 | 2.476382 |
| C | -3.345279 | -9.021292 | 1.592700 |
| C | -2.433255 | -8.293589 | 0.818861 |
| C | 0.617350 | -9.582555 | -1.799605 |
| C | 1.548506 | -10.280930 | -2.585882 |
| C | 2.904583 | -10.002976 | -2.476415 |
| C | 3.345308 | -9.021209 | -1.592727 |
| C | 2.433267 | -8.293531 | -0.818884 |
| C | -3.007138 | -7.226553 | -0.092144 |
| C | 0.840370 | -9.956572 | 1.979555 |
| C | -0.840321 | -9.956583 | -1.979580 |
| C | 3.007124 | -7.226482 | 0.092123 |
| C | -0.114345 | -6.099031 | 2.587970 |
| C | 0.114344 | -6.098953 | -2.587944 |
| N | -3.934208 | -10.744394 | 3.303947 |
| N | 3.934277 | -10.744288 | -3.303983 |
| C | 4.906472 | -11.465456 | -2.388158 |
| C | 3.320518 | -11.778994 | -4.218656 |
| C | -4.906389 | -11.465578 | 2.388119 |
| C | -3.320426 | -11.779093 | 4.218612 |
| C | 0.000002 | -1.902100 | 0.000075 |
| C | 0.000002 | -0.679503 | 0.000093 |
| H | -0.065308 | 3.496173 | -2.142582 |
| H | 0.065305 | 3.496236 | 2.142686 |
| H | 1.171883 | 11.033543 | 3.266207 |
| H | 4.400160 | 8.789645 | 1.488219 |
| H | -1.171958 | 11.033501 | -3.266243 |
| H | -4.400184 | 8.789537 | -1.488247 |
| H | 2.797206 | 6.222957 | 0.291435 |

| | | | |
|---|---|---|---|
| H | 2.593009 | 7.271525 | -1.103586 |
| H | 4.092502 | 7.330086 | -0.179393 |
| H | -1.475034 | 9.079799 | 2.141917 |
| H | -0.974236 | 10.614628 | 2.842644 |
| H | -1.229527 | 10.480485 | 1.100501 |
| H | 1.475001 | 9.079822 | -2.141945 |
| H | 0.974172 | 10.614644 | -2.842667 |
| H | 1.229467 | 10.480501 | -1.100525 |
| H | -2.797161 | 6.222890 | -0.291450 |
| H | -2.593000 | 7.271471 | 1.103568 |
| H | -4.092492 | 7.329985 | 0.179368 |
| H | -0.625620 | 6.898033 | 2.700726 |
| H | -0.084357 | 5.364739 | 3.373418 |
| H | 1.097347 | 6.537054 | 2.792240 |
| H | 0.084354 | 5.364636 | -3.373368 |
| H | -1.097343 | 6.536976 | -2.792228 |
| H | 0.625626 | 6.897948 | -2.700723 |
| H | -5.416657 | 10.739729 | -1.757860 |
| H | -4.342210 | 12.165067 | -1.771142 |
| H | -5.632271 | 11.999284 | -3.003745 |
| H | -2.792813 | 12.521859 | -3.621499 |
| H | -2.641479 | 11.288624 | -4.915341 |
| H | -4.129715 | 12.258206 | -4.770000 |
| H | 5.416593 | 10.739859 | 1.757828 |
| H | 4.342113 | 12.165172 | 1.771096 |
| H | 5.632174 | 11.999428 | 3.003704 |
| H | 2.792700 | 12.521939 | 3.621450 |
| H | 2.641400 | 11.288711 | 4.915303 |
| H | 4.129611 | 12.258330 | 4.769951 |
| H | 0.065308 | -3.496173 | -2.142582 |

| | | | |
|---|---|---|---|
| H | -0.065305 | -3.496236 | 2.142686 |
| H | -1.171883 | -11.033543 | 3.266207 |
| H | -4.400160 | -8.789645 | 1.488219 |
| H | 1.171958 | -11.033501 | -3.266243 |
| H | 4.400184 | -8.789537 | -1.488247 |
| H | -2.797206 | -6.222957 | 0.291435 |
| H | -2.593009 | -7.271525 | -1.103586 |
| H | -4.092502 | -7.330086 | -0.179393 |
| H | 1.475034 | -9.079799 | 2.141917 |
| H | 0.974236 | -10.614628 | 2.842644 |
| H | 1.229527 | -10.480485 | 1.100501 |
| H | -1.475001 | -9.079822 | -2.141945 |
| H | -0.974172 | -10.614644 | -2.842667 |
| H | -1.229467 | -10.480501 | -1.100525 |
| H | 2.797161 | -6.222890 | -0.291450 |
| H | 2.593000 | -7.271471 | 1.103568 |
| H | 4.092492 | -7.329985 | 0.179368 |
| H | 0.625620 | -6.898033 | 2.700726 |
| H | 0.084357 | -5.364739 | 3.373418 |
| H | -1.097347 | -6.537054 | 2.792240 |
| H | -0.084354 | -5.364636 | -3.373368 |
| H | 1.097343 | -6.536976 | -2.792228 |
| H | -0.625626 | -6.897948 | -2.700723 |
| H | 4.342210 | -12.165067 | -1.771142 |
| H | 5.632271 | -11.999284 | -3.003745 |
| H | 5.416657 | -10.739729 | -1.757860 |
| H | 2.641479 | -11.288624 | -4.915341 |
| H | 4.129715 | -12.258206 | -4.770000 |
| H | 2.792813 | -12.521859 | -3.621499 |
| H | -5.416593 | -10.739859 | 1.757828 |
| H | -4.342113 | -12.165172 | 1.771096 |
| H | -5.632174 | -11.999428 | 3.003704 |
| H | -2.792700 | -12.521939 | 3.621450 |
| H | -2.641400 | -11.288711 | 4.915303 |
| H | -4.129611 | -12.258330 | 4.769951 |
| C | 4.694723 | -9.760825 | -4.175024 |
| H | 3.980127 | -9.252503 | -4.822755 |
| H | 5.205395 | -9.037211 | -3.542641 |
| H | 5.422574 | -10.315107 | -4.769809 |
| C | -4.694692 | -9.761018 | 4.174960 |
| H | -3.980110 | -9.252694 | 4.822707 |
| H | -5.205374 | -9.037402 | 3.542589 |
| H | -5.422534 | -10.315328 | 4.769729 |
| C | -4.694723 | 9.760825 | -4.175024 |
| H | -3.980127 | 9.252503 | -4.822755 |
| H | -5.205395 | 9.037211 | -3.542641 |
| H | -5.422574 | 10.315107 | -4.769809 |
| C | 4.694692 | 9.761018 | 4.174960 |
| H | 3.980110 | 9.252694 | 4.822707 |
| H | 5.205374 | 9.037402 | 3.542589 |
| H | 5.422534 | 10.315328 | 4.769729 |

## References


[1] C.-W. Chiu, F. P. Gabbai, *Organometallics* **2008**, *27*, 1657-1659.
[2] S.-F. Liu, Q. Wu, H. L. Schmider, H. Aziz, N.-X. Hu, Z. Popović, S. Wang, *J. Am. Chem. Soc.* **2000**, *122*, 3671-3678.
[3] T. Itoh, K. Matsuda, H. Iwamura, K. Hori, *J. Am. Chem. Soc.* **2000**, *122*, 2567-2576.
[4] T. Fujihara, Y. Tomike, T. Ohtake, J. Terao, Y. Tsuji, *Chem. Commun.* **2011**, *47*, 9699-9701.
[5] G. Sheldrick, *Acta Crystalogr.* **2015**, *A71*, 3-8.
[6] G. Sheldrick, *Acta Crystalogr.* **2008**, *A64*, 112-122.
[7] K. Brandenburg, *DIAMOND*, Crystal Impact Gbr, Bonn, Germany, **2007**.
[8] J. B. Chaires, N. Dattagupta, D. M. Crothers, *Biochemistry* **1982**, *21*, 3933.
[9] L. M. Tumir, I. Piantanida, I. J. Cindric, T. Hrenar, Z. Meic, M. Zinic, *J. Phys. Org. Chem.* **2003**, *16*, 891-899.
[10] W. Saenger, *Principles of Nucleic Acid Structure*, Springer-Verlag: New York, **1983**, 226.
[11] C. R. Cantor, P. R. Schimmel, *Biophys. Chem.,* **1980**, *3*, 1109-1181.
[12] J. L. Mergny, L. Lacroix, *Oligonucleotides* **2003**, *13*, 515-537.
[13] a) G. Scatchard, *Ann. N.Y. Acad. Sci.* **1949,** *51*, 660–672; b) J. D. McGhee and P. H. V. Hippel, Journal of Molecular Biology 1974, 86, 469-489.
[14] A. Rozman, I. Crnolatac, T. Deligeorgiev and I. Piantanida, *J. Lumin.* **2019**, *205*, 87-96.
[15] C. H. Munro, W. E. Smith, M. Garner, J. Clarkson, P. C. White, *Langmuir* **1995**, *11*, 3712–3720.
[16] Gaussian 09, Revision A.02, M. J. Frisch, G. W. Trucks, H. B. Schlegel, G. E. Scuseria, M. A. Robb, J. R. Cheeseman, G. Scalmani, V. Barone, G. A. Petersson, H. Nakatsuji, X. Li, M. Caricato, A. Marenich, J. Bloino, B. G. Janesko, R. Gomperts, B. Mennucci, H. P. Hratchian, J. V. Ortiz, A. F. Izmaylov, J. L. Sonnenberg, D. Williams-Young, F. Ding, F. Lipparini, F. Egidi, J. Goings, B. Peng, A. Petrone, T. Henderson, D. Ranasinghe, V. G. Zakrzewski, J. Gao, N. Rega, G. Zheng, W. Liang, M. Hada, M. Ehara, K. Toyota, R. Fukuda, J. Hasegawa, M. Ishida, T. Nakajima, Y. Honda, O. Kitao, H. Nakai, T. Vreven, K. Throssell, J. A. Montgomery, Jr., J. E. Peralta, F. Ogliaro, M. Bearpark, J. J. Heyd, E. Brothers, K. N. Kudin, V. N. Staroverov, T. Keith, R. Kobayashi, J. Normand, K. Raghavachari, A. Rendell, J. C. Burant, S. S. Iyengar, J. Tomasi, M. Cossi, J. M. Millam, M. Klene, C. Adamo, R. Cammi, J. W. Ochterski, R. L. Martin, K. Morokuma, O. Farkas, J. B. Foresman, and D. J. Fox, Gaussian, Inc., Wallingford CT, **2016**.
[17] T. Lu, F. Chen, *J. Comput. Chem.* **2012**, *33*, 580-592.
[18] A. D. Becke, *J. Chem. Phys.* **1993**, *98*, 5648-5652.
[19] a) G. A. Petersson, M. A. Al-Laham, *J. Chem. Phys.* A **1991**, *94*, 6081-6090; b) G. A. Petersson, A. Bennett, T. G. Tensfeldt, M. A. Al-Laham, W. A.Shirley, *J. Chem. Phys.* **1988**, *89*, 2193-2218.
[20] J. Tomasi, B. Mennucci, R. Cammi, *Chem. Rev.* **2005**, *105*, 2999-3093.
[21] T. Yanai, D. P. Tew, N. C. Handy, *Chem. Phys. Lett.* **2004**, *393*, 51-57.
[21] A. P. Scott, L. Radom, *J. Phys. Chem.* **1996**, *100*, 16502-16513.
